# The Concise Guide to PHARMACOLOGY 2023/24: Ion channels

**DOI:** 10.1111/bph.16178

**Published:** 2023-10

**Authors:** Stephen P. H. Alexander, Alistair A. Mathie, John A. Peters, Emma L. Veale, Jörg Striessnig, Eamonn Kelly, Jane F. Armstrong, Elena Faccenda, Simon D. Harding, Jamie A. Davies, Richard W. Aldrich, Bernard Attali, Austin M. Baggetta, Elvir Becirovic, Martin Biel, Roslyn M. Bill, Ana I. Caceres, William A. Catterall, Alex C. Conner, Paul Davies, Katrien De Clerq, Markus Delling, Francesco Di Virgilio, Simonetta Falzoni, Stefanie Fenske, Anna Fortuny-Gomez, Samuel Fountain, Chandy George, Steve A. N. Goldstein, Christian Grimm, Stephan Grissmer, Kotdaji Ha, Verena Hammelmann, Israel Hanukoglu, Meiqin Hu, Ad P. Ijzerman, Sairam V. Jabba, Mike Jarvis, Anders A. Jensen, Sven E. Jordt, Leonard K. Kaczmarek, Stephan Kellenberger, Charles Kennedy, Brian King, Philip Kitchen, Qiang Liu, Joseph W. Lynch, Jessica Meades, Verena Mehlfeld, Annette Nicke, Stefan Offermanns, Edward Perez-Reyes, Leigh D. Plant, Lachlan Rash, Dejian Ren, Mootaz M. Salman, Werner Sieghart, Lucia G. Sivilotti, Trevor G. Smart, Terrance P. Snutch, Jinbin Tian, James S. Trimmer, Charlotte Van den Eynde, Joris Vriens, Aguan D. Wei, Brenda T. Winn, Heike Wulff, Haoxing Xu, Fan Yang, Wei Fang, Lixia Yue, Xiaoli Zhang, Michael Zhu

**Affiliations:** 1 School of Life Sciences, University of Nottingham Medical School, Nottingham, NG7 2UH, UK; 2 School of Engineering, Arts, Science and Technology, University of Suffolk, Ipswich, IP4 1QJ, UK; 3 Neuroscience Division, Medical Education Institute, Ninewells Hospital and Medical School, University of Dundee, Dundee, DD1 9SY, UK; 4 Medway School of Pharmacy, The Universities of Greenwich and Kent at Medway, Anson Building, Central Avenue, Chatham Maritime, Chatham, Kent, ME4 4TB, UK; 5 Pharmacology and Toxicology, Institute of Pharmacy, University of Innsbruck, A-6020, Innsbruck, Austria; 6 School of Physiology, Pharmacology and Neuroscience, University of Bristol, Bristol, BS8 1TD, UK; 7 Centre for Discovery Brain Sciences, University of Edinburgh, Edinburgh, EH8 9XD, UK; 8 Stanford University, Austin, USA; 9 Tel Aviv University, Tel Aviv, Israel; 10 Northeastern University, Boston, USA; 11 Ludwig Maximilian University of Munich, Munich, Germany; 12 Aston University, Birmingham, UK; 13 Duke University, Durham, USA; 14 University of Washington, Seattle, USA; 15 University of Birmingham, Birmingham, UK; 16 Tufts University School of Medicine, Boston, USA; 17 Catholic University of Leuven, Leuven, Belgium; 18 University of California San Francisco, San Francisco, USA; 19 University of Ferrara, Ferrara, Italy; 20 University of East Anglia, Norwich, UK; 21 Nanyang Technological University, Singapore, Singapore; 22 University of California Irvine, Irvine, USA; 23 Ulm University, Ulm, Germany; 24 Ariel University, Ariel, Israel; 25 Hangzhou, China; 26 Leiden University, Leiden, Netherlands; 27 University of Illinois, Chicago, USA; 28 University of Copenhagen, Copenhagen, Denmark; 29 Yale University, New Haven, USA; 30 University of Lausanne, Lausanne, Switzerland; 31 Strathclyde University, Glasgow, UK; 32 University College London, London, UK; 33 University of Queensland, Brisbane, Australia; 34 Max Planck Institute for Heart and Lung Research/JW Goethe University, Bad Nauheim/Frankfurt, Germany; 35 University of Virginia, Charlottesville, USA; 36 University of Queensland, St. Lucia, Australia; 37 University of Pennsylvania, Philadelphia, USA; 38 University of Oxford, Oxford, UK; 39 Medical University of Vienna, Vienna, Austria; 40 University of British Columbia, Vancouver, Canada; 41 University of Texas at Houston, Houston, USA; 42 University of California Davis, Davis, USA; 43 University of Michigan, Ann Arbor, USA; 44 University of Connecticut, Farmington, USA

## Abstract

The Concise Guide to PHARMACOLOGY 2023/24 is the sixth in this series of biennial publications. The Concise Guide provides concise overviews, mostly in tabular format, of the key properties of approximately 1800 drug targets, and over 6000 interactions with about 3900 ligands. There is an emphasis on selective pharmacology (where available), plus links to the open access knowledgebase source of drug targets and their ligands (https://www.guidetopharmacology.org/), which provides more detailed views of target and ligand properties. Although the Concise Guide constitutes almost 500 pages, the material presented is substantially reduced compared to information and links presented on the website. It provides a permanent, citable, point-in-time record that will survive database updates. The full contents of this section can be found at http://onlinelibrary.wiley.com/doi/10.1111/bph.16178. Ion channels are one of the six major pharmacological targets into which the Guide is divided, with the others being: G protein-coupled receptors, nuclear hormone receptors, catalytic receptors, enzymes and transporters. These are presented with nomenclature guidance and summary information on the best available pharmacological tools, alongside key references and suggestions for further reading. The landscape format of the Concise Guide is designed to facilitate comparison of related targets from material contemporary to mid-2023, and supersedes data presented in the 2021/22, 2019/20, 2017/18, 2015/16 and 2013/14 Concise Guides and previous Guides to Receptors and Channels. It is produced in close conjunction with the Nomenclature and Standards Committee of the International Union of Basic and Clinical Pharmacology (NC-IUPHAR), therefore, providing official IUPHAR classification and nomenclature for human drug targets, where appropriate.

## Graphical Abstract

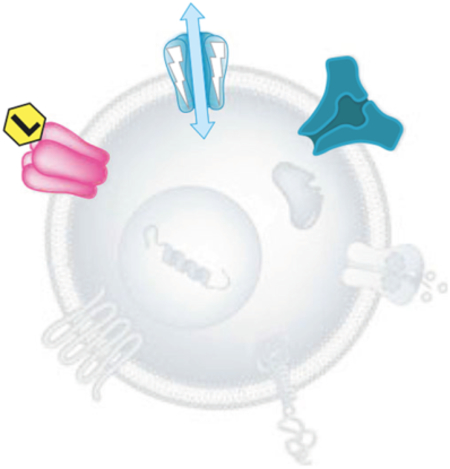

## Overview:

Ion channels are pore-forming proteins that allow the flow of ions acrossp[aouxww membranes, either plasma membranes, or the membranes of intracellular organelles [463]. Many ion channels (such as most Na, K, Ca and some Cl channels) are gated by voltage but others (such as certain K and Cl channels, TRP channels, ryanodine receptors and IP_3_ receptors) are relatively voltage-insensitive and are gated by second messengers and other intracellular and/or extracellular mediators. As such, there is some blurring of the boundaries between "ion channels" and "ligand-gated channels" which are compiled separately in the Guide. Resolution of ion channel structures, beginning with K channels [295] then Cl channels [313] and most recently Na channels [944] has greatly improved understanding of the structural basis behind ion channel function. Many ion channels (*e.g.,* K, Na, Ca, HCN and TRP channels) share several structural similarities. These channels are thought to have evolved from a common ancestor and have been classfied together as the "voltage-gated-like (VGL) ion channel chanome" (see [1380]). Other ion channels, however, such as Cl channels, aquaporins and connexins, have completely different structural properties to the VGL channels, having evolved quite separately.

Currently, ion channels (including ligand-gated ion channels) represent the second largest target for existing drugs after G protein-coupled receptors [921]. However, the advent of novel, faster screening techniques for compounds acting on ion channels [305] suggests that these proteins represent promising targets for the development of additional, novel therapeutic agents for the near future.

## Ligand-gated ion channels


Ion channels→Ligand-gated ion channels

### Overview:

Ligand-gated ion channels (LGICs) are integral membrane proteins that contain a pore which allows the regulated flow of selected ions across the plasma membrane. Ion flux is passive and driven by the electrochemical gradient for the permeant ions. These channels are open, or gated, by the binding of a neurotransmitter to an orthosteric site(s) that triggers a conformational change that results in the conducting state. Modulation of gating can occur by the binding of endogenous, or exogenous, modulators to allosteric sites. LGICs mediate fast synaptic transmission, on a millisecond time scale, in the nervous system and at the somatic neuromuscular junction. Such transmission involves the release of a neurotransmitter from a pre-synaptic neurone and the subsequent activation of post-synaptically located receptors that mediate a rapid, phasic, electrical signal (the excitatory, or inhibitory, post-synaptic potential). However, in addition to their traditional role in phasic neurotransmission, it is now established that some LGICs mediate a tonic form of neuronal regulation that results from the activation of extrasynaptic receptors by ambient levels of neurotransmitter. The expression of some LGICs by non-excitable cells is suggestive of additional functions.

By convention, the LGICs comprise the excitatory, cation-selective, nicotinic acetylcholine [170, 817], 5-HT_3_ [68, 1277], ionotropic glutamate [728, 1225] and P2X receptors [529, 1169] and the inhibitory, anion-selective, GABA_A_ [91, 908] and glycine receptors [747, 1371]. The nicotinic acetylcholine, 5-HT_3_, GABA_A_ and glycine receptors (and an additional zinc-activated channel) are pentameric structures and are frequently referred to as the Cys-loop receptors due to the presence of a defining loop of residues formed by a disulphide bond in the extracellular domain of their constituent subunits [821, 1199]. However, the prokaryotic ancestors of these receptors contain no such loop and the term pentameric ligand-gated ion channel (pLGIC) is gaining acceptance in the literature [459]. The ionotropic glutamate and P2X receptors are tetrameric and trimeric structures, respectively. Multiple genes encode the subunits of LGICs and the majority of these receptors are heteromultimers. Such combinational diversity results, within each class of LGIC, in a wide range of receptors with differing pharmacological and biophysical properties and varying patterns of expression within the nervous system and other tissues. The LGICs thus present attractive targets for new therapeutic agents with improved discrimination between receptor isoforms and a reduced propensity for off-target effects. The development of novel, faster screening techniques for compounds acting on LGICs [305] will greatly aid in the development of such agents.

## 5-HT_3_ receptors


$$\text{Ion channels}\to \text{Ligand-gated ion channels}\to 5\text{-}{\text{HT}}_{3}\;\text{receptors}$$

### Overview:

The 5-HT_3_ receptor (**nomenclature as agreed by the NC-IUPHAR Subcommittee on 5-Hydroxytryptamine (serotonin) receptors** [487]) is a ligand-gated ion channel of the Cys-loop family that includes the zinc-activated channels, nicotinic acetylcholine, GABA_A_ and strychnine-sensitive glycine receptors. The receptor exists as a pentamer of 4 transmembrane (TM) subunits that form an intrinsic cation selective channel [68]. Five human 5-HT_3_ receptor subunits have been cloned and homo-oligomeric assemblies of 5-HT_3_A and hetero-oligomeric assemblies of 5-HT_3_A and 5-HT_3_B subunits have been characterised in detail. The 5-HT_3_C (*HTR3C*, Q8WXA8), 5-HT_3_D (*HTR3D,* Q70Z44) and 5-HT_3_E (*HTR3E,* A5X5Y0) subunits [574, 873], like the 5-HT_3_B subunit, do not form functional homomers, but are reported to assemble with the 5-HT_3_A subunit to influence its functional expression rather than pharmacological profile [475, 875, 1276]. 5-HT_3_A, -C, -D, and -E subunits also interact with the chaperone RIC-3 which predominantly enhances the surface expression of homomeric 5-HT_3_A receptor [1276]. The co-expression of 5-HT_3_A and 5-HT_3_C-E subunits has been demonstrated in human colon [568]. A recombinant hetero-oligomeric 5-HT_3_AB receptor has been reported to contain two copies of the 5-HT_3_A subunit and three copies of the 5-HT_3_B subunit in the order B-B-A-B-A [73], but this is inconsistent with recent reports which show at least one A-A interface [727, 1203]. The 5-HT_3_B subunit imparts distinctive biophysical properties upon hetero-oligomeric 5-HT_3_AB versus homo-oligomeric 5-HT_3_A recombinant receptors [241, 302, 428, 531, 585, 963, 1142], influences the potency of channel blockers, but generally has only a modest effect upon the apparent affinity of agonists, or the affinity of antagonists ([126], but see [238, 258, 302]) which may be explained by the orthosteric binding site residing at an interface formed between 5-HT_3_A subunits [727, 1203]. However, 5-HT_3_A and 5-HT_3_AB receptors differ in their allosteric regulation by some general anaesthetic agents, small alcohols and indoles [490, 1046, 1133]. The potential diversity of 5-HT_3_ receptors is increased by alternative splicing of the genes *HTR3A* and *HTR3E* [136, 480, 872, 874, 875]. In addition, the use of tissue-specific promoters driving expression from different transcriptional start sites has been reported for the *HTR3A, HTR3B, HTR3D* and *HTR3E* genes, which could result in 5-HT_3_ subunits harbouring different N-termini [531, 872, 1231]. To date, inclusion of the 5-HT_3_A subunit appears imperative for 5-HT_3_ receptor function.

**Table:** Complexes

Nomenclature	5-HT_3_AB	5-HT_3_A
Subunits	5-HT_3_A, 5-HT_3_B	5-HT_3_A
Agonists	CSTI-300 (Partial agonist) [1025]	CSTI-300 (Partial agonist) [1025]
Selective agonists	–	meta-chlorphenylbiguanide [90, 241, 659, 828, 829], 2-methyl-5-HT [90, 241, 659, 828], SR57227A [316] – Rat, 1-phenylbiguanide [90]
Antagonists	–	vortioxetine (pK_i_ 8.4) [63], metoclopramide (pK_i_ 6–6.4) [126, 481]
Selective antagonists	–	palonosetron (pK_i_ 10.5) [840], alosetron (pK_i_ 9.5) [466], (S)-zacopride (pK_i_ 9) [126], granisetron (pK_i_ ~8.6–8.8) [481, 828], tropisetron (pK_i_ 8.5–8.8) [659, 828], ondansetron (pK_i_ ~7.8–8.3) [126, 481, 828]
Channel blockers	picrotoxinin (pIC_50_ 4.2) [1198], bilobalide (pIC_50_ 2.5) [1198], ginkgolide B (pIC_50_ 2.4) [1198]	picrotoxinin (pIC_50_ 5) [1197], TMB-8 (pIC_50_ 4.9) [1163], diltiazem (pIC_50_ 4.7) [1197], bilobalide (pIC_50_ 3.3) [1197], ginkgolide B (pIC_50_ 3.1) [1197]
Labelled ligands	–	[^3^H]ramosetron (Antagonist) (pK_d_ 9.8) [828], [^3^H]GR65630 (Antagonist) (pK_d_ 8.6–9.3) [466, 659], [^3^H]granisetron (Antagonist) (pK_d_ 8.9) [126, 481], [^3^H](S)-zacopride (Antagonist) (pK_d_ 8.7) [934], [^3^H]LY278584 (Antagonist) (pK_d_ 8.5) [3]
Functional Characteristics	*γ* = 0.4-0.8 pS [+ 5-HT_3_B, *γ* = 16 pS]; inwardly rectifying current [+ 5-HT_3_B, rectification reduced]; n_H_ 2-3 [+ 5-HT_3_B 1-2]; relative permeability to divalent cations reduced by co-expression of the 5-HT_3_B subunit	*γ* = 0.4-0.8 pS [+ 5-HT_3_B, *γ* = 16 pS]; inwardly rectifying current [+ 5-HT_3_B, rectification reduced]; n_H_ 2-3 [+ 5-HT_3_B 1-2]; relative permeability to divalent cations reduced by co-expression of the 5-HT_3_B subunit

**Table:** Subunits

Nomenclature	5-HT_3_A	5-HT_3_B	5-HT_3_C	5-HT_3_D	5-HT_3_E
HGNC, UniProt	*HTR3A,* P46098	*HTR3B,* O95264	*HTR3C,* Q8WXA8	*HTR3D,* Q70Z44	*HTR3E,* A5X5Y0
Functional Characteristics	*γ* = 0.4-0.8 pS [+ 5-HT_3_B, *γ* = 16 pS]; inwardly rectifying current [+ 5-HT_3_B, rectification reduced]; n_H_ 2-3 [+ 5-HT_3_B 1-2]; relative permeability to divalent cations reduced by co-expression of the 5-HT_3_B subunit	*γ* = 0.4-0.8 pS [+ 5-HT_3_B, *γ* = 16 pS]; inwardly rectifying current [+ 5-HT_3_B, rectification reduced]; n_H_ 2-3 [+ 5-HT_3_B 1-2]; relative permeability to divalent cations reduced by co-expression of the 5-HT_3_B subunit	–	–	–

### Comments:

Quantitative data in the table refer to homo-oligomeric assemblies of the human 5-HT_3_A subunit, or the receptor native to human tissues. Significant changes introduced by co-expression of the 5-HT_3_B subunit are indicated in parenthesis. Although not a selective antagonist, methadone displays multimodal and subunit-dependent antagonism of 5-HT_3_receptors [258]. Similarly, TMB-8, diltiazem, picrotoxin, bilobalide and ginkgolide B are not selective for 5-HT_3_ receptors (*e.g.*[1198]). The anti-malarial drugs mefloquine and quinine exert a modestly more potent block of 5-HT_3_A versus 5-HT_3_AB receptor-mediated responses [1200]. Known better as a partial agonist of nicotinic acetylcholine *α*4*β*2 receptors, varenicline is also an agonist of the 5-HT_3_A receptor [743]. Human [90, 828], rat [510], mouse [780], guinea-pig [659] ferret [830] and canine [534] orthologues of the 5-HT_3_A receptor subunit have been cloned that exhibit intraspecies variations in receptor pharmacology. Notably, most ligands display significantly reduced affinities at the guinea-pig 5-HT_3_ receptor in comparison with other species. In addition to the agents listed in the table, native and recombinant 5-HT_3_ receptors are subject to allosteric modulation by extracellular divalent cations, alcohols, several general anaesthetics and 5-hydroxy- and halide-substituted indoles (see reviews [25, 26, 935, 1201, 1202, 1277]).

## Acid-sensing (proton-gated) ion channels (ASICs)


Ion channels→Ligand-gated ion channels→Acid-sensing (proton-gated) ion channels (ASICs)

### Overview:

Acid-sensing ion channels (ASICs, **nomenclature as agreed by NC-IUPHAR** [25, 26, 584]) are members of a Na^+^ channel superfamily that includes the epithelial Na^+^ channel (ENaC), the FMRF-amide activated channel (FaNaC) of invertebrates, the degenerins (DEG) of *Caenorhabitis elegans,* channels in *Drosophila melanogaster* and‘orphan’ channels that include BLINaC [1053] and INaC [1071] that have also been named BASICS, for bile acid-activated ion channels [1318].

ASIC subunits contain 2 TM domains and assemble as homo- or hetero-trimers [53, 392, 530, 1162, 1374, 1375] to form protongated, voltage-insensitive, Na^+^ permeable, channels that are activated by levels of acidosis occurring in both physiological and pathophysiological conditions with ASIC3 also playing a role in mechanosensation (reviewed in [198, 410, 584, 1032, 1314]). Splice variants of ASIC1 [termed ASIC1a (ASIC, ASIC*α*, BNaC2*α*) [1269], ASIC1b (ASIC*β*, BNaC2*β*) [181] and ASIC1b2 (ASIC*β*2) [1233]; note that ASIC1a is also permeable to Ca^2+^], ASIC2 [termed ASIC2a (MDEG1, BNaC1*α*, BNC1*α*) [372, 990, 1271] and ASIC2b (MDEG2, BNaC1*β*) [711]] differ in the first third of the protein. Unlike ASIC2a (listed in table), heterologous expression of ASIC2b alone does not support H^+^-gated currents. A third member, ASIC3 (DRASIC, TNaC1) [1268] is one of the most pH-sensitive isoforms (along with ASIC1a) and has the fastest activation and desensitisation kinetics, however can also carry small sustained currents. ASIC4 (SPASIC) evolved as a proton-sensitive channel but seems to have lost this function in mammals [745]. Mammalian ASIC4 does not support a proton-gated channel in heterologous expression systems but is reported to downregulate the expression of ASIC1a and ASIC3 [16, 290, 409, 707]. ASICs channels are primarily expressed in central (ASIC1a, -2a, 2b and -4) and peripheral neurons including nociceptors (ASIC1-3) where they participate in neuronal sensitivity to acidosis. Humans express, in contrast to rodents, ASIC3 also in the brain [261]. ASICs have also been detected in taste receptor cells (ASIC1-3)), photoreceptors and retinal cells (ASIC1-3), cochlear hair cells (ASIC1b), testis (hASIC3), pituitary gland (ASIC4), lung epithelial cells (ASIC1a and -3), urothelial cells, adipose cells (ASIC3), vascular smooth muscle cells (ASIC1-3), immune cells (ASIC1,-3 and -4) and bone (ASIC1-3) (ASIC distribution is reviewed in [269, 408, 708]). A neurotransmitter-like function of protons has been suggested, involving postsynaptically located ASICs of the CNS in functions such as learning and fear perception [299, 640, 1425], responses to focal ischemia [1342] and to axonal degeneration in autoimmune inflammation in a mouse model of multiple sclerosis [362], as well as seizures [1426] and pain [116, 270, 271, 283, 1314]. Heterologously expressed heteromultimers form ion channels with differences in kinetics, ion selectivity, pH- sensitivity and sensitivity to blockers that resemble some of the native proton activated currents recorded from neurones [51, 72, 329, 711]. In general, the known small molecule inhibitors of ASICs are non-selective or partially selective, whereas the venom peptide inhibitors have substantially higher selectivity and potency. Several clinically used drugs are known to inhibit ASICs, however they are generally more potent at other targets (*e.g.* amiloride at ENaCs, ibuprofen at COX enzymes) [916, 1016]. The information in the tables below are for the effects of inhibitors on homomeric channels, for information of known effects on heteromeric channels see the comments below.

**Table:** 

Nomenclature	ASIC1	ASIC2
HGNC, UniProt	*ASIC1,* P78348	*ASIC2,* Q16515
Endogenous activators	Extracellular H^+^ (ASIC1a) (pEC_50_ ~6.2–6.8), Extracellular H^+^ (ASIC1b) (pEC_50_ ~5.1–6.2)	Extracellular H^+^ (pEC_50_ ~4.1–5)
Channel blockers	Pi-hexatoxin-Hi1a (ASIC1a) (pIC_50_ ~9.3) [135], psalmotoxin 1 (ASIC1a) (pIC_50_ 9) [329], Pi-theraphotoxin-Hm3a (ASIC1a) (pIC_50_ ~8.5) [323], Zn^2+^ (ASIC1a) (pIC_50_ ~8.2) [204], JNJ-799760 (pIC_50_ 7.6) [724], JNJ-67869386 (pIC_50_ 7.5) [724], mambalgin-1 (ASIC1a) (pIC_50_ ~7.3) [283], compound 5b (pIC_50_ 7.2) [142], ASC06-IgC1 (Inhibition) (pIC_50_ ~7.1) [999], mambalgin-1 (ASIC1b) (pIC_50_ ~7) [69], diminazene (ASIC1a & ASIC1b) (pIC_50_ ~6.5) [637, 670, 1077], NS383 (pIC_50_ 6.4) [845], Pb^2+^ (ASIC1b) (pIC_50_ ~5.8), A-317567 (ASIC1a) (pIC_50_ ~5.7) [301] – Rat, Pb^2+^ (ASIC1a) (pIC_50_ ~5.4) [1292], compound 5b (pIC_50_ 5.2) [142], amiloride (ASIC1a) (pIC_50_ 5), benzamil (ASIC1a) (pIC_50_ 5) [1269], ethylisopropylamiloride (ASIC1a) (pIC_50_ 5) [1269], nafamostat (ASIC1a) (pIC_50_ ~4.9) [1232], amiloride (ASIC1b) (pIC_50_ 4.6–4.7) [1269], flurbiprofen (ASIC1a) (pIC_50_ 3.5) [1258] – Rat, ibuprofen (ASIC1a) (pIC_50_ ~3.5) [746, 1258]	diminazene (pIC_50_ ~6.1) [670], amiloride (pIC_50_ 4.6) [1271], A-317567 (pIC_50_ ~4.5) [301], nafamostat (pIC_50_ ~4.2) [1232], Cd^2+^ (Partial inhibition) (pIC_50_ ~3)[1141]
Labelled ligands	[^125^I]psalmotoxin 1 (ASIC1a) (pK_d_ 9.7) [1058]	–
Functional Characteristics	ASIC1a: *γ* =14pS P_Na_/P_K_ = 5-13, P_Na_/P_Ca_ =2.5 rapid activation rate (5.8-13.7 ms), rapid inactivation rate (1.2-4 s) @ pH 6.0, slow recovery (5.3-13s) @ pH 7.4 ASIC1b: *γ* =19 pS P_Na_/P_K_ =14.0, P_Na_ ≫ P_Ca_ rapid activation rate (9.9 ms), rapid inactivation rate (0.9-1.7 s) @ pH 6.0, slow recovery (4.4-77 s) @ pH 7.4	*γ*=10.4-13.4 pS P_Na_/P_K_ =10, P_Na_/P_Ca_ = 20 rapid activation rate, moderate inactivation rate (3.3-5.5 s) @ pH 5
Comments	ASIC1a and ASIC1b are activated by the heteromeric Texas coral snake toxin MitTx, with pEC_50_ values of ~8 and ~7.6 respectively [116].	ASIC2 is also blocked by other diarylamidines [190].

**Table:** 

Nomenclature	ASIC3
HGNC, UniProt	*ASIC3,* Q9UHC3
Endogenous activators	Extracellular H^+^ (transient component) (pEC_50_ ~6.2–6.7), lysophosphatidylcholine (Partial agonist) (pEC_50_ 5.4) [782], Extracellular H^+^ (sustained component) (pEC_50_ ~3.5–4.3)
Activators	GMQ (largly non-desensitizing; at pH 7.4) (pEC_50_ ~3) [1387], arcaine (at pH 7.4) (pEC_50_ ~2.9) [696], agmatine (at pH 7.4) (pEC_50_ ~2) [696]
Channel blockers	APETx2 (transient component only) (pIC_50_ 7.2) [282], diminazene (pIC_50_ ~6.5) [670], A-317567 (pIC_50_ 6) [645], NS383 (pIC_50_ 5.7) [845], nafamostat (transient component) (pIC_50_ ~5.6) [1232], Ugr 9-1 (transient component) (pIC_50_ 5) [917], amiloride (transient component only - sustained component enhanced by 200*μ*M amiloride at pH 4) (pIC_50_ 4.2–4.8) [1268], Gd^3+^ (pIC_50_ 4.4) [51], Zn^2+^ (pIC_50_ 4.2) [540], aspirin (sustained component) (pIC_50_ 4) [1258], diclofenac (sustained component) (pIC_50_ 4) [1258], salicylic acid (sustained component) (pIC_50_ 3.6) [1258]
Functional Characteristics	*γ*=13-15 pS; biphasic response consisting of rapidly inactivating transient and sustained components; very rapid activation (<5 ms) and inactivation (0.4 s); fast recovery (0.4-0.6 s) @ pH 7.4, transient component partially inactivated at pH 7.2
Comments	ASIC3 is activated by Mit-Toxin (pEC_50_ ~6.1) [116].

### Comments:

Psalmotoxin 1 (PcTx1) inhibits ASIC1a by increasing the affinity to H+ and promoting channel desensitization [188, 329]. PcTx1 has little effect on ASIC2a, ASIC3 or ASIC1a expressed as a heteromultimer with ASIC3 but does inhibit ASIC1a expressed as a heteromultimer with ASIC2a [545] or ASIC2b [1102]. PcTx1 and *π*-Hm3a potentiate ASIC1b currents [189, 323]. ASIC1-containing homo- and heteromers are inhibited by Mambalgins, toxins contained in the black mamba venom, which induce in ASIC1a an acidic shift of the pH dependence of activation [283]. *π*-Hi1a is selective for ASIC1a with mild potentiating activity at ASIC1b. It inhibits channel activation and is very slowly reversible [172]. APETx2 most potently blocks homomeric ASIC3 channels, but also ASIC2b+ASIC3, ASIC1b+ASIC3, and ASIC1a+ASIC3 heteromeric channels with IC_50_ values of 117 nM, 900 nM and 2 *μ*M, respectively. APETx2 has no effect on ASIC1a or ASIC2a+ASIC3, however, it does potentiate ASIC1b and ASIC2a homomers in the low micromolar range (1-10 *μ*M) [282, 284, 670]. APETx2 however also inhibits voltage-gated Na_+_ channels [108, 953]. The antibody ASC06-IgG1 binds to the structurally intact channel in the upper part of the extracellular domain with substantial contact on the finger domain and is highly selective for ASIC1a over other subtypes [999]. IC_50_ value for A-317567 was determined using high throughput electrophysiology on human ASIC3 expressed in HEK293 cells [645]. For some of the newer small molecule inhibitors it is not known whether they inhibit ion channels in addition to ASICs [142, 724, 845]. The effects of several compounds are pH-dependent, displaying higher potencies at more alkaline pH [142, 724, 845].

The pEC_50_ values for proton activation of ASIC channels are influenced by numerous factors including extracellular di- and poly-valent ions, Zn^2+^, protein kinase C and serine proteases (reviewed in [584, 1314]). Rapid acidification is required for activation of ASIC1 and ASIC3 due to fast inactivation/desensitization. pEC_50_ values for H^+^-activation of either transient, or sustained, currents mediated by ASIC3 vary in the literature and may reflect species and/or methodological differences [52, 247, 1268]. The transient ASIC current component is Na^+^-selective (PNa/PK of about 10) [1268, 1362] whereas the sustained current component that is observed with ASIC3 and some ASIC heteromers is non-selective between Na^+^ and K^+^ [247]. The reducing agents dithiothreitol (DTT) and glutathione (GSH) increase ASIC1a currents expressed in CHO cells and ASIC-like currents in sensory ganglia and central neurons [37, 203] whereas oxidation, through the formation of intersubunit disulphide bonds, reduces currents mediated by ASIC1a [1399]. ASIC1a is also irreversibly modulated by extracellular serine proteases, such as trypsin, through proteolytic cleavage [1263]. Non-steroidal anti-inflammatory drugs (NSAIDs) are direct inhibitors of ASIC currents (reviewed in [70]). Extracellular Zn^2+^ potentiates proton activation of homomeric and heteromeric channels incorporating ASIC2a, but not homomeric ASIC1a or ASIC3 channels [71]. However, removal of contaminating Zn^2+^ by chelation reveals a high affinity block of homomeric ASIC1a and heteromeric ASIC1a+ASIC2 channels by Zn^2+^ indicating complex biphasic actions of the divalent [204]. Nitric oxide potentiates submaximal currents activated by H^+^ mediated by ASIC1a, ASIC1b, ASIC2a and ASIC3 [145]. The positive modulation of homomeric, heteromeric and native ASIC channels by the peptide FMRFamide and related substances, such as neuropeptides FF and SF, is reviewed in detail in [1247]. Inflammatory conditions and particular pro-inflammatory mediators such as arachidonic acid induce overexpression of ASIC-encoding genes and enhance ASIC currents [271, 776, 1122]. The sustained current component mediated by ASIC3 is potentiated by hypertonic solutions in a manner that is synergistic with the effect of arachidonic acid [271]. ASIC3 is partially activated by the lipids lysophosphatidylcholine (LPC) and arachidonic acid [782]. Mit-Toxin, which is contained in the venom of the Texas coral snake, activates several ASIC subtypes [116]. Selective activation of ASIC3 by GMQ, likely by binding to the central vestibule, is potentiated by mild acidosis and reduced extracellular Ca^2+^ [1387].

#### Additional notes on the channels:

Until recently they were thought to be vertebrate specific channels, however are now known to have evolved over 600 million years ago and appear to be conserved throughout the superphylum of animals known as deuterostomes (which includes vertebrates, tunicates, starfish, sea urchins, sea cucumbers and acorn worms) [745]. Recently an ion-conducting-independent signaling mechanism has been revealed for ASIC1a whereby the acidosis-activated channel recruits RIPK1 to its C-terminus resulting in RIPK1 phosphorylation and activation of necroptosis. This pathways is suggested to be the primary cause of ASIC-mediated neuronal cell death in ischemic stroke [1286, 1300].

## Epithelial sodium channel (ENaC)


Ion channels→Ligand-gated ion channels→Epithelial sodium channel (ENaC)

### Overview:

The epithelial sodium channels (ENaC) are located on the apical membrane of epithelial cells in the kidney tubules, lung, respiratory tract, male and female reproductive tracts, sweat and salivary glands, placenta, colon, and some other organs [147, 303, 433, 434, 1095]. In these epithelia, Na^+^ ions flow from the extracellular fluid into the cytoplasm of epithelial cells *via* ENaC and are then pumped out of the cytoplasm into the interstitial fluid by the Na^+^/K^+^ ATPase located on the basolateral membrane [977]. As Na^+^ is one of the major electrolytes in the extracellular fluid (ECF), osmolarity change initiated by the Na^+^ flow is accompanied by a flow of water [123]. Thus, ENaC has a central role in regulating ECF volume and blood pressure, primarily *via* its function in the kidney [1035]. The expression of ENaC subunits, hence its activity, is regulated by the renin-angiotensin-aldosterone system, and other factors involved in electrolyte homeostasis [614, 1035].

The genetics of the hereditary systemic pseudohypoaldosteronism type-I revealed that the activity of ENaC is dependent on three subunits encoded by three genes [169, 434]. Within the protein superfamily that includes ENaC, the crystal structure of ASIC was determined first, revealing a trimeric structure with a large extracellular domain anchored in the membrane with a bundle of six TM helices (two TM helices/subunit) [53, 530]. The first 3D structure of human ENaC was determined by single-particle cryo-electron microscopy at a resolution of 3.7 Ç [892]. A recent study improved the resolution to 3 Ç [893]. These structures confirmed that ENaC has a 3D quaternary structure similar to ASIC. ENaC is assembled as a hetero-trimer with a clockwise order of *α-γ-β* subunit viewed from the top, as shown previously [219]. In contrast to ASIC1 which can assemble into a functional homotrimer, ENaC activity can be reconstituted fully only as a heterotrimer with an *αβγ* or a *δβγ* composition [584].

In the respiratory tract and female reproductive tract, large segments of the epithelia are composed of multi-ciliated cells. In these cells, ENaC is located along the entire length of the cilia that cover the cell surface [322]. Cilial location greatly increases ENaC density per cell surface and allows ENaC to serve as a sensitive regulator of osmolarity of the periciliary fluid throughout the whole depth of the fluid bathing the cilia [322]. In contrast to ENaC, CFTR (ion transporter defective in cystic fibrosis) is located on the non-cilial cell surface [322]. In the *vas deferens* segment of the male reproductive tract, the luminal surface is covered by microvilli and stereocilia projections with backbones composed of actin filament bundles [1095]. In these cells, both ENaC and the water channel aquaporin AQP9 are localized on these projections and also in the basal and smooth muscle layers [1095]. Thus, ENaC function regulates the volume of fluid lining epithelia essential for mucociliary clearance of respiratory airways, transport of germ cells, fertilization, implantation, and cell migration [322, 434, 788].

### Genes and Phylogeny

In the human genome, there are four homologous genes (*SCNN1A, SCNN1B, SCNN1D,* and *SCNN1G*) that encode four proteins, *α-, β-, γ-,* and *δ*-ENaC that may be involved in the assembly of ENaC [148, 712, 1069, 1270]. These four subunits share 23-34% sequence identity and <20% identity with ASIC subunits [434]. The genes coding for all four ENaC subunits are present in all bony vertebrates with the exception of ray-finned fish genomes that have lost all ENaC genes. The mouse genome has lost the gene *SCNN1D* that codes for *δ*-ENaC [388, 434, 434]. The *α-, β-,* and *γ*-ENaC genes are also present in jawless vertebrates (*e.g.,* lampreys) and cartilaginous fishes (*e.g.,* sharks) [434]. Examination of the methylation patterns of the 5′-flanking region of *SCNN1A, SCNN1B,* and *SCNN1G* genes in human cells showed an inverse correlation between gene expression and DNA methylation, suggesting epigenetic transcriptional control of ENaC genes [970].

### Channel biogenesis, assembly and function

The expression of ENaC subunits is regulated primarily by aldosterone and many additional extracellular and intracellular factors [611, 926, 1035]. Most of the studies indicate that the expression of the three subunits is not coordinated [143]. However, the transport of the subunits to the membrane is dependent on three intact subunits. Even a missense mutation in one subunit reduces the concentration of assembled channels on the cell surface [314].

ENaC is a constitutively active channel, *i.e.,* the flow of Na^+^ ions is not dependent on an activating factor. Hence, heterologous cells expressing ENaC (*e.g., Xenopus* oocytes), must be maintained in a solution that contains amiloride to keep ENaC inhibited. To measure ENaC activity, the bath solution is switched to a solution without amiloride. ENaC has two major states: 1) Open, and 2) Closed. The probability of ENaC being in the open state is called ENaC open probability (Po). ENaC activity is regulated by a diverse array of factors that exert their effects by modifying, directly or indirectly, two major parameters: 1) The density of ENaC in the membrane; and 2) The channel open probability [576, 584]. The Po of ENaC is greatly decreased by external Na^+^ and this response is called Na^+^ self-inhibition [107, 483, 1099].

An important aspect of ENaC regulation is that the *α* and the *γ* subunits have conserved serine protease cleavage sites in the extracellular segment [434]. Cleavage of these subunits by proteases such as furin and plasmin leads to the activation of ENaC [29, 610, 1036].

### Diseases associated with ENaC mutations

Mutations in any of the three genes (*SCNN1A, SCNN1B,* and *SCNN1G*) may cause partial or complete loss of ENaC activity, depending on the mutation [169, 431]. Such loss-of-function mutations are associated with a syndrome named "systemic" or "multi-system" autosomal recessive pseudohypoaldosteronism type I (PHA1B) [169, 322, 430, 434, 1068, 1397]. So far, no mutation has been found in the *SCNN1D* gene that causes PHA. PHA patients suffer from severe salt loss from all aldosterone target organs expressing ENaC, including kidney, sweat and salivary glands and respiratory tract. During infancy and early childhood, the severe electrolyte disturbances, dehydration and acidosis may require recurrent hospitalizations. The severity and frequency of salt-wasting episodes improve with age [432]. PHA1B is also associated with a dysfunctional female reproductive system [114, 322].

The carboxy-terminal of ENaC includes a short consensus sequence called the PY motif. Mutations in this motif in *SCNN1B* and *SCNN1G* are associated with Liddle syndrome, which is characterized by early-onset hypertension [113, 1104]. The PY motif is recognized by Nedd4-2 that is a ubiquitin ligase. Thus, mutations in the PY motif reduce ubiquitylation of ENaC leading to the accumulation of ENaC in the membrane, consequently enhance the activity of ENaC [1037].

### ENaC expression in tumors

The observation that [Na^+^] is higher in many cancerous cells as compared to non-cancerous cells has led to the suggestion that enhanced expression of ENaC may be responsible for increased metastasis [684]. However, analysis of RNA sequencing data of ENaC-encoding genes, and clinical data of cervical cancer patients from The Cancer Genome Atlas showed a negative correlation with histologic grades of tumor [1134]. Similarly, studies on breast cancer cells that altered *α*-ENaC levels by over-expression or siRNA-mediated knockdown showed that increased *α*-ENaC expression was associated with decreased breast cancer cell proliferation [1302]. In contrast, analysis of RNA sequencing data from The Cancer Genome Atlas showed that high expression of *SCNN1A* was correlated with poor prognosis in patients with ovarian cancer [734]. These findings indicate that the association of ENaC levels with tumorigenesis varies depending on the tissue.

### COVID-19

The surface of SARS-CoV-2 virions that cause COVID-19 is covered by many glycosylated S (spike) proteins. These S proteins bind to the membrane-bound angiotensin-converting enzyme 2 (ACE2) as a first step in the entry of the virion into the host cell. Viral entry into the cell is dependent on the cleavage of the S protein (at Arg-667/Ser-668) by a serine-protease. Anand *et al.* showed that this cleavage site has a sequence motif that is homologous to the furin cleavage site in *α*-ENaC [30]. A comprehensive review on the pathological consequences of COVID-19 suggests a role for ENaC in the early phases of COVID-19 infection in the respiratory tract epithelia [380].

**Table:** Complexes

Nomenclature	ENaC*αβγ*
Subunits	ENaC *α*, ENaC *β*, ENaC *γ*
Activators	S3969 (pEC_50_ 5.9) [740]
Channel blockers	P552-02 (pIC_50_ 8.1), benzamil (pIC_50_ ~8), amiloride (pIC_50_ 6.7–7), triamterene (pIC_50_ ~5.3) [148, 583]

**Table:** Subunits

Nomenclature	ENaC *α*	ENaC *β*	ENaC *γ*	ENaC *δ*
HGNC, UniProt	*SCNN1A,* P37088	*SCNN1B,* P51168	*SCNN1G,* P51170	*SCNN1D,* P51172

## GABA_A_ receptors


$$\text{Ion channels}\to \text{Ligand-gated ion channels}\to {\text{GaBA}}_{A}\;\text{receptors}$$

### Overview:

The GABA_A_ receptor is a ligand-gated ion channel of the Cys-loop family that includes the nicotinic acetylcholine, 5-HT_3_ and strychnine-sensitive glycine receptors. GABA_A_ receptor-mediated inhibition within the CNS occurs by fast synaptic transmission, sustained tonic inhibition and temporally intermediate events that have been termed ‘GABA_A_, slow’ [156]. GABA_A_ receptors exist as pentamers of 4TM subunits that form an intrinsic anion selective channel. Sequences of six *α*, three *β*, three *γ*, one *δ*, three *ρ*, one *ϵ*, one *π* and one *θ* GABA_A_ receptor subunits have been reported in mammals [908, 909, 1107, 1111]. The *π*-subunit is restricted to reproductive tissue. Alternatively spliced versions of many subunits exist (e.g. *α* 4- and *α*6- (both not functional) *α*5-, *β*2-, *β*3- and *γ*2), along with RNA editing of the *α*3 subunit [237]. The three *ρ*-subunits, (*ρ*1-3) function as either homo- or hetero-oligomeric assemblies [179, 1400]. Receptors formed from *ρ*-subunits, because of their distinctive pharmacology that includes insensitivity to bicuculline, benzodiazepines and barbiturates, have sometimes been termed GABA_C_ receptors [1400], **but they are classified as GABA_A_ receptors by NC-IUPHAR on the basis of structural and functional criteria** [67, 908, 909].

Many GABA_A_ receptor subtypes contain *α-, β-* and *γ*-subunits with the likely stoichiometry 2*α*.2*β*.1*γ* [627, 908]. It is thought that the majority of GABA_A_ receptors harbour a single type of *α* and *β* -subunit variant. The *α*1*β*2*γ*2 hetero-oligomer constitutes the largest population of GABA_A_ receptors in the CNS, followed by the *α*2*β*3*γ*2 and *α*3*β*3*γ*2 isoforms. Receptors that incorporate the *α*4- *α*5-or *α*6-subunit, or the *β*1-, *γ*1-, *γ*3-, *δ*-, *ϵ*- and *θ*-subunits, are less numerous, but they may nonetheless serve important functions. For example, extrasynaptically located receptors that contain *α*6- and *δ*-subunits in cerebellar granule cells, or an *α*4- and *δ*-subunit in dentate gyrus granule cells and thalamic neurones, mediate a tonic current that is important for neuronal excitability in response to ambient concentrations of GABA [91, 341, 831, 1088, 1128]. GABA binding occurs at the *β*+*α*- subunit interface and the homologous *γ*+/*α*- subunits interface creates the benzodiazepine site. A second site for benzodiazepine binding has recently been postulated to occur at the *α*+*β*- interface ([1009]; reviewed by [1110]). The particular *α*-and *γ*-subunit isoforms exhibit marked effects on recognition and/or efficacy at the benzodiazepine site. Thus, receptors incorporating either *α*4- or *α*6-subunits are not recognised by ‘classical’ benzodiazepines, such as flunitrazepam (but see [1376]). The trafficking, cell surface expression, internalisation and function of GABA_A_ receptors and their subunits are discussed in detail in several recent reviews [195, 518, 744, 1252] but one point worthy of note is that receptors incorporating the *γ*2 subunit (except when associated with *α*5) cluster at the postsynaptic membrane (but may distribute dynamically between synaptic and extrasynaptic locations), whereas those incorporating the *δ* subunit appear to be exclusively extrasynaptic.

**NC-IUPHAR** [25, 26, 67, 908] class the GABA_A_ receptors according to their subunit structure, pharmacology and receptor function. Currently, eleven native GABA_A_ receptors are classed as conclusively identified (*i.e., α*1*β*2*γ*2, *α*2*βγ*2, *α*3*βγ*2, *α*4*βγ*2, *α*4*β* 2*δ*, *α*4*β*3*δ*, *α*5*βγ*2, *α*6*βγ*2, *α*6*β*2*δ*, *α*6*β*3*δ* and *ρ*) with further receptor isoforms occurring with high probability, or only tentatively [908, 909]. It is beyond the scope of this Guide to discuss the pharmacology of individual GABA_A_ receptor isoforms in detail; such information can be gleaned in the reviews [45, 46, 67, 364, 547, 627, 642, 832, 908, 909, 1107, 1109]. Agents that discriminate between *α*-subunit isoforms are noted in the table and additional agents that demonstrate selectivity between receptor isoforms, for example *via β*-subunit selectivity, are indicated in the text below. The distinctive agonist and antagonist pharmacology of *ρ* receptors is summarised in the table and additional aspects are reviewed in [179, 548, 869, 1400].

Several high-resolution cryo-electron microscopy structures have been described in which the full-length human *α*1*β*3*γ*2L GABA_A_ receptor in lipid nanodiscs is bound to the channel-blocker picrotoxin, the competitive antagonist bicuculline, the agonist GABA (*γ*-aminobutyric acid), and the classical benzodiazepines alprazolam and diazepam [787].

**Table:** 

Nomenclature	GABA_A_ receptor *α*1 subunit	GABA_A_ receptor *α*2 subunit
HGNC, UniProt	*GABRA1,* P14867	*GABRA2,* P47869
Agonists	gaboxadol [GABA site], isoguvacine [GABA site], isonipecotic acid [GABA site], muscimol [GABA site], piperidine-4-sulphonic acid [GABA site]	gaboxadol [GABA site], isoguvacine [GABA site], isonipecotic acid [GABA site], muscimol [GABA site], piperidine-4-sulphonic acid [GABA site]
Antagonists	bicuculline [GABA site], gabazine [GABA site]	bicuculline [GABA site], gabazine [GABA site]
Channel blockers	TBPS, picrotoxin	TBPS, picrotoxin
Allosteric modulators	flumazenil [benzodiazepine site] (Antagonist) (pK_i_ 9.1) [1109], *α*3IA [benzodiazepine site] (Inverse agonist) [1109], *α*5IA [benzodiazepine site] (Inverse agonist) [1109], DMCM [benzodiazepine site] (Inverse agonist) [1008]	flumazenil [benzodiazepine site] (Antagonist at *α*1 receptors, but allosteric modulator at other subtypes.) (pK_i_ 9.1) [1109], *α*3IA [benzodiazepine site] (Inverse agonist) [1109], *α*5IA [benzodiazepine site] (Inverse agonist) [1109], DMCM [benzodiazepine site] (Inverse agonist) [1008]
Endogenous allosteric modulators	5*α*-pregnan-3*α*-ol-20-one (Potentiation), Zn^2+^ (Inhibition), tetrahydrodeoxycorticosterone (Potentiation)	5*α*-pregnan-3*α*-ol-20-one (Potentiation), Zn^2+^ (Inhibition), tetrahydrodeoxycorticosterone (Potentiation)
Allosteric modulators (Positive)	clonazepam [benzodiazepine site] (pK_i_ 8.9) [991], flunitrazepam [benzodiazepine site] (pK_i_ 8.3) [421], diazepam [benzodiazepine site] (pK_i_ 7.8) [991], alprazolam [benzodiazepine site] (pEC_50_ 7.4) [20]	clonazepam [benzodiazepine site] (pK_i_ 8.8) [991], flunitrazepam [benzodiazepine site] (pK_i_ 8.3) [421], alprazolam [benzodiazepine site] (pEC_50_ 7.9) [20], diazepam [benzodiazepine site] (pK_i_ 7.8) [991]
Selective allosteric modulators	zolpidem (Positive) (pK_i_ 7.4–77) [422, 1080, 1109], L838417 [benzodiazepine site] (Antagonist) [1109]	L838417 [benzodiazepine site] (Partial agonist) [1109], TPA023 [benzodiazepine site] (Partial agonist) [1109]
Labelled ligands	[^11^C]flumazenil [benzodiazepine site] (Allosteric modulator, Antagonist), [^18^F]fluoroethylflumazenil [benzodiazepine site] (Allosteric modulator), [^35^S]TBPS [anion channel] (Channel blocker), [^3^H]CGS8216 [benzodiazepine site] (Allosteric modulator, Mixed), [^3^H]flunitrazepam [benzodiazepine site] (Allosteric modulator), [^3^H]gabazine [GABA site] (Antagonist), [^3^H]muscimol [GABA site] (Agonist), [^3^H]zolpidem [benzodiazepine site] (Allosteric modulator)	[^11^C]flumazenil [benzodiazepine site] (Allosteric modulator), [^18^F]fluoroethylflumazenil [benzodiazepine site] (Allosteric modulator), [^35^S]TBPS [anion channel] (Channel blocker), [^3^H]CGS8216 [benzodiazepine site] (Allosteric modulator, Mixed), [^3^H] flunitrazepam [benzodiazepine site] (Allosteric modulator, Full agonist), [^3^H]gabazine [GABA site] (Antagonist), [^3^H]muscimol [GABA site] (Agonist)
Comments	Zn^2+^ is an endogenous allosteric regulator and causes potent inhibition of receptors formed from binary combinations of *α* and *β* subunits, incorporation of a *δ* or *γ* subunit causes a modest, or pronounced, reduction in inhibitory potency, respectively [641]	Zn^2+^ is an endogenous allosteric regulator and causes potent inhibition of receptors formed from binary combinations of *α* and *β* subunits, incorporation of a *δ* or *γ* subunit causes a modest, or pronounced, reduction in inhibitory potency, respectively [641]

**Table:** 

Nomenclature	GABA_A_ receptor *α*3 subunit	GABA_A_ receptor *α*4 subunit
HGNC, UniProt	*GABRA3,* P34903	*GABRA4,* P48169
Agonists	gaboxadol [GABA site], isoguvacine [GABA site], isonipecotic acid [GABA site], muscimol [GABA site], piperidine-4-sulphonic acid [GABA site]	gaboxadol [GABA site], isoguvacine [GABA site], muscimol [GABA site], piperidine-4-sulphonic acid [GABA site] (low efficacy)
Selective agonists	–	isonipecotic acid [GABA site] (relatively high efficacy, partially selective)
Antagonists	bicuculline [GABA site], gabazine [GABA site]	bicuculline [GABA site], gabazine [GABA site]
Channel blockers	TBPS, picrotoxin	TBPS, picrotoxin
Allosteric modulators	flumazenil [benzodiazepine site] (Antagonist at *α*1 receptors, but allosteric modulator at other subtypes.) (pK_i_ 9) [1008, 1109], *α*5IA [benzodiazepine site] (Inverse agonist) [1109], DMCM [benzodiazepine site] (Inverse agonist) [1008]	bretazenil [benzodiazepine site] (Full agonist) [1008]
Endogenous allosteric modulators	5*α*-pregnan-3*α*-ol-20-one (Potentiation), Zn^2+^ (Inhibition), tetrahydrodeoxycorticosterone (Potentiation)	5*α*-pregnan-3*α*-ol-20-one (Potentiation), Zn^2+^ (Inhibition), tetrahydrodeoxycorticosterone (Potentiation)
Allosteric modulators (Positive)	clonazepam [benzodiazepine site] (pK_i_ 8.7) [991], flunitrazepam [benzodiazepine site] (pK_i_ 7.8) [421], diazepam [benzodiazepine site] (pK_i_ 7.8) [991], alprazolam [benzodiazepine site] (pEC_50_ 7.2) [20]	
Selective allosteric modulators	*α*3IA [benzodiazepine site] (higher affinity, partially selective) [1109], L838417 [benzodiazepine site] (Partial agonist) [1109], Ro19-4603 [benzodiazepine site] (Inverse agonist), TP003 [benzodiazepine site] (Partial agonist) [1109], TPA023 [benzodiazepine site] (Partial agonist) [1109]	Ro15-4513 [benzodiazepine site] (Full agonist)
Labelled ligands	[^11^C]flumazenil [benzodiazepine site] (Allosteric modulator), [^18^F]fluoroethylflumazenil [benzodiazepine site] (Allosteric modulator), [^35^S]TBPS [anion channel] (Channel blocker), [^3^H]CGS8216 [benzodiazepine site] (Allosteric modulator, Mixed), [^3^H]flunitrazepam [benzodiazepine site] (Allosteric modulator, Full agonist), [^3^H]gabazine [GABA site] (Antagonist), [^3^H]muscimol [GABA site] (Agonist)	[^11^C]flumazenil [benzodiazepine site] (Allosteric modulator, Partial agonist), [^18^F]fluoroethylflumazenil [benzodiazepine site] (Allosteric modulator), [^35^S]TBPS [anion channel] (Channel blocker), [^3^H]CGS8216 [benzodiazepine site] (Allosteric modulator, Mixed), [^3^H]Ro154513 [benzodiazepine site] (Allosteric modulator, Full agonist), [^3^H]gabazine [GABA site] (Antagonist), [^3^H]muscimol [GABA site] (Agonist)
Comments	Zn^2+^ is an endogenous allosteric regulator and causes potent inhibition of receptors formed from binary combinations of *α* and *β* subunits, incorporation of a *δ* or *γ* subunit causes a modest, or pronounced, reduction in inhibitory potency, respectively [641]	Diazepam and flunitrazepam are not active at this subunit. Zn^2+^ is an endogenous allosteric regulator and causes potent inhibition of receptors formed from binary combinations of *α* and *β* subunits, incorporation of a *δ* or *α* subunit causes amodest, or pronounced, reduction in inhibitory potency, respectively [641]. [^3^H]Ro154513 labels *α*4*βγ*2 and *α*6*βγ*2 receptors in the presence of a saturating concentration of a ‘classical’ benzodiazepine (*e.g.* diazepam).

**Table:** 

Nomenclature	GABA_A_ receptor *α*5 subunit	GABA_A_ receptor *α*6 subunit
HGNC, UniProt	*GABRA5,* P31644	*GABRA6,* Q16445
Agonists	gaboxadol [GABA site], isoguvacine [GABA site], isonipecotic acid [GABA site], muscimol [GABA site], piperidine-4-sulphonic acid [GABA site]	gaboxadol [GABA site], isoguvacine [GABA site], muscimol [GABA site], piperidine-4-sulphonic acid [GABA site] (low efficacy)
Selective agonists	–	isonipecotic acid [GABA site] (relatively high efficacy, relatively selective)
Antagonists	bicuculline [GABA site], gabazine [GABA site]	bicuculline [GABA site], gabazine [GABA site]
Channel blockers	TBPS, picrotoxin	TBPS, picrotoxin
Allosteric modulators	flumazenil [benzodiazepine site] (Antagonist at *α*1 receptors, but allosteric modulator at other subtypes.) (pK_i_ 9.2) [1109], *α*3IA [benzodiazepine site] (Inverse agonist) [1109], DMCM [benzodiazepine site] (Inverse agonist) [1008]	flumazenil [benzodiazepine site] (Partial agonist) (pK_i_ 6.8) [1109], bretazenil [benzodiazepine site] (Full agonist) [1008]
Endogenous allosteric modulators	5*α*-pregnan-3*α*-ol-20-one (Potentiation), Zn^2+^ (Inhibition), tetrahydrodeoxycorticosterone (Potentiation)	5*α*-pregnan-3*α*-ol-20-one (Potentiation), Zn^2+^ (Inhibition), tetrahydrodeoxycorticosterone (Potentiation)
Allosteric modulators (Positive)	flunitrazepam [benzodiazepine site] (pK_i_ 8.3) [421], alprazolam [benzodiazepine site] (pEC_50_ 8) [20]	–
Selective allosteric modulators	*α*5IA [benzodiazepine site] (Inverse agonist) [1109], L655708 [benzodiazepine site] (Inverse agonist) [1008], L838417 [benzodiazepine site] (Partial agonist) [1109], MRK016 [benzodiazepine site] (Inverse agonist) [1109], RO4938581 [benzodiazepine site] (Inverse agonist) [1109], RY024 [benzodiazepine site] (Inverse agonist) [1109]	LAU159 (Full agonist) [1108, 1109], LAU463 (Full agonist) [1108, 1109], PZ-II-029 (Full agonist) [1108, 1109], Ro15-4513 [benzodiazepine site] (Full agonist), amiloride (Antagonist) [1109], furosemide (Antagonist) [1109]
Labelled ligands	[^3^H]RY80 [benzodiazepine site] (Selective Binding) (pK_d_ 9.2) [1117] – Rat, [^11^C] flumazenil [benzodiazepine site] (Allosteric modulator), [^18^F]fluoroethylflumazenil [benzodiazepine site] (Allosteric modulator), [^35^S]TBPS [anion channel] (Channel blocker), [^3^H]CGS8216 [benzodiazepine site] (Allosteric modulator, Mixed), [^3^H]L655708 [benzodiazepine site] (Allosteric modulator, Inverse agonist), [^3^H] flunitrazepam [benzodiazepine site] (Allosteric modulator, Full agonist), [^3^H] gabazine [GABA site] (Antagonist), [^3^H]muscimol [GABA site] (Agonist)	[^11^C]flumazenil [benzodiazepine site] (Allosteric modulator, Partial agonist), [^18^F]fluoroethylflumazenil [benzodiazepine site] (Allosteric modulator), [^35^S]TBPS [anion channel] (Channel blocker), [^3^H]CGS8216 [benzodiazepine site] (Allosteric modulator, Mixed), [^3^H] Ro154513 [benzodiazepine site] (Allosteric modulator, Full agonist), [^3^H]gabazine [GABA site] (Antagonist), [^3^H]muscimol [GABA site] (Agonist)
Comments	Zn^2+^ is an endogenous allosteric regulator and causes potent inhibition of receptors formed from binary combinations of *α* and *β* subunits, incorporation of a *δ* or *γ* subunit causes a modest, or pronounced, reduction in inhibitory potency, respectively [641]	Diazepam and flunitrazepam are not active at channels containing this subunit. Zn^2+^ is an endogenous allosteric regulator and causes potent inhibition of receptors formed from binary combinations of *α* and *β* subunits, incorporation of a *δ* or *γ* subunit causes amodest, or pronounced, reduction in inhibitory potency, respectively [641]. [^3^H]Ro154513 selectively labels *α*6-subunit and *α*4-subunit-containing receptors in the presence of a saturating concentration of a ‘classical’ benzodiazepine (*e.g.* diazepam). Sieghart *et al.* (2022) provides a review of the pharmacology of *α*6-containing GABA_A_ receptors.

**Table:** 

Nomenclature	GABA_A_ receptor *β*1 subunit	GABA_A_ receptor *β*2 subunit	GABA_A_ receptor *β*3 subunit
HGNC, UniProt	*GABRB1,* P18505	*GABRB2,* P47870	*GABRB3,* P28472
Channel blockers	TBPS, picrotoxin	TBPS, picrotoxin	TBPS, picrotoxin
Allosteric modulators	–	–	etazolate (Binding) (pIC_50_ 5.5) [1398]
Comments	Zn^2+^ is an endogenous allosteric regulator and causes potent inhibition of receptors formed from binary combinations of *α* and *β* subunits, incorporation of a *δ* or *γ* subunit causes a modest, or pronounced, reduction in inhibitory potency, respectively [641]	Zn^2+^ is an endogenous allosteric regulator and causes potent inhibition of receptors formed from binary combinations of *α* and *β* subunits, incorporation of a *δ* or *γ* subunit causes a modest, or pronounced, reduction in inhibitory potency, respectively [641]	Zn^2+^ is an endogenous allosteric regulator and causes potent inhibition of receptors formed from binary combinations of *α* and *β* subunits, incorporation of a *δ* or *γ* subunit causes a modest, or pronounced, reduction in inhibitory potency, respectively [641]

**Table:** 

Nomenclature	GABA_A_ receptor *γ*1 subunit	GABA_A_ receptor *γ*2 subunit	GABA_A_ receptor *γ*3 subunit
HGNC, UniProt	*GABRG1,* Q8N1C3	*GABRG2,* P18507	*GABRG3*, Q99928
Channel blockers	TBPS, picrotoxin	TBPS, picrotoxin	TBPS, picrotoxin
Comments	Zn^2+^ is an endogenous allosteric regulator and causes potent inhibition of receptors formed from binary combinations of *α* and *β* subunits, incorporation of a *δ* or *γ* subunit causes a modest, or pronounced, reduction in inhibitory potency, respectively [641], So far, the only publication investigating the pharmacology of more than three or four ligands at *γ*1 receptors was Khom *et al.* (2006) [592].	Zn^2+^ is an endogenous allosteric regulator and causes potent inhibition of receptors formed from binary combinations of *α* and *β* subunits, incorporation of a *δ* or *γ* subunit causes a modest, or pronounced, reduction in inhibitory potency, respectively [641]	Zn^2+^ is an endogenous allosteric regulator and causes potent inhibition of receptors formed from binary combinations of *α* and *β* subunits, incorporation of a *δ* or *γ* subunit causes a modest, or pronounced, reduction in inhibitory potency, respectively [641]

**Table:** 

Nomenclature	GABA_A_ receptor *δ* subunit	GABA_A_ receptor *ϵ* subunit	GABA_A_ receptor *θ* subunit
HGNC, UniProt	*GABRD,* O14764	*GABRE*, P78334	*GABRQ*, Q9UN88
Selective agonists	DS2 [1109], gaboxadol [GABA site], tracazolate [1109]	–	–
Channel blockers	TBPS, picrotoxin	TBPS, picrotoxin	TBPS, picrotoxin
Comments	Zn^2+^ is an endogenous allosteric regulator and causes potent inhibition of receptors formed from binary combinations of *α* and *β* subunits, incorporation of a *δ* or *γ* subunit causes a modest, or pronounced, reduction in inhibitory potency, respectively	–	–

**Table:** 

Nomenclature	GABA_A_ receptor *π* subunit	GABA_A_ receptor *ρ*1 subunit	GABA_A_ receptor *ρ*2 subunit	GABA_A_ receptor *ρ*3 subunit
HGNC, UniProt	*GABRP,* O00591	*GABRR1,* P24046	*GABRR2,* P28476	*GABRR3,* A8MPY1
Agonists	–	isoguvacine [GABA site] (Partial agonist), muscimol [GABA site] (Partial agonist)	isoguvacine [GABA site] (Partial agonist), muscimol [GABA site] (Partial agonist)	isoguvacine [GABA site] (Partial agonist), muscimol [GABA site] (Partial agonist)
Selective agonists	–	(±)-cis-2-CAMP [GABA site], 5-Me-IAA [GABA site]	(±)-cis-2-CAMP [GABA site], 5-Me-IAA [GABA site]	(±)-cis-2-CAMP [GABA site], 5-Me-IAA [GABA site]
Antagonists	–	gaboxadol [GABA site], isonipecotic acid [GABA site], piperidine-4-sulphonic acid [GABA site]	gaboxadol [GABA site], isonipecotic acid [GABA site], piperidine-4-sulphonic acid [GABA site]	gaboxadol [GABA site], isonipecotic acid [GABA site], piperidine-4-sulphonic acid [GABA site]
Selective antagonists	–	cis-3-ACPBPA [GABA site], trans-3-ACPBPA [GABA site], TPMPA [GABA site], aza-THIP [GABA site]	cis-3-ACPBPA [GABA site], trans-3-ACPBPA [GABA site], TPMPA [GABA site], aza-THIP [GABA site]	cis-3-ACPBPA [GABA site], trans-3-ACPBPA [GABA site], TPMPA [GABA site], aza-THIP [GABA site]
Channel blockers	TBPS, picrotoxin	TBPS, picrotoxin	TBPS, picrotoxin	TBPS, picrotoxin
Comments	–	Bicuculline is not active at this subunit	Bicuculline is not active at this subunit	Bicuculline is not active at this subunit

### Comments:

The potency and efficacy of many GABA agonists vary between GABA_A_ receptor isoforms [364, 573, 642]. For example, gaboxadol is a partial agonist at receptors with the sub-unit composition *α*4*β*3*γ*2, but elicits currents in excess of those evoked by GABA at the *α*4*β*3*δ* receptor where GABA itself is a low efficacy agonist [101, 134]. The antagonists bicuculline and gabazine differ in their ability to suppress spontaneous openings of the GABA_A_ receptor, the former being more effective [1206]. The presence of the *γ* subunit within the heterotrimeric complex reduces the potency and efficacy of agonists [1150]. The GABA_A_ receptor contains multiple allosteric binding sites. Most drugs modulating GABA_A_ receptors can bind to several different sites [336]. Distinct allosteric sites bind barbiturates and endogenous (*e.g.,* 5*α*-pregnan-3*α*-ol-20-one) and synthetic (*e.g.,* alphaxalone) neuroactive steroids in a diastereo- or enantio-selective manner [92, 452, 484, 1242]. Picrotoxinin and TBPS act at an allosteric site within the chloride channel pore to negatively regulate channel activity; negative allosteric regulation by *γ*-butyrolactone derivatives also involves the picrotoxinin site, whereas positive allosteric regulation by such compounds is proposed to occur at a distinct locus. Many intravenous (*e.g.,* etomidate, propofol) and inhalational (*e.g.,* halothane, isoflurane) anaesthetics and alcohols also exert a regulatory influence upon GABA_A_ receptor activity [120, 907]. Specific amino acid residues within GABA_A_ receptor *α*- and *β*-subunits that influence allosteric regulation by anaesthetic and non-anaesthetic compounds have been identified [449, 484]. Photoaffinity labelling of distinct amino acid residues within purified GABA_A_receptors by the etomidate derivative, [^3^H]azietomidate, has also been demonstrated [691], and this binding is subject to positive allosteric regulation by neurosteroids [690An array of natural products including flavonoid and terpenoid compounds exert varied actions at GABA_A_receptors (reviewed in detail in [547]).

In addition to the agents listed in the table, modulators of GABA_A_ receptor activity that exhibit subunit dependent activity include: salicylidene salicylhydrazide (negative allosteric modulator selective for *β*1- versus *β*2-, or *β*3-subunit-containing receptors [1207]); fragrant dioxane derivatives (positive allosteric modulators selective for *β*1- versus *β*2-, or *β*3-subunit-containing receptors [1091]); loreclezole, etomidate, tracazolate, mefenamic acid, etifoxine, stiripentol, valerenic acid amide (positive allosteric modulators with selectivity for *β*2/*β*3- over *β*1-subunit-containing receptors [353, 593, 627]); tracazolate (intrinsic efficacy, *i.e.,* potentiation, or inhibition, is dependent upon the identity of the *γ*1-3-, *δ*-, or *ϵ*-subunit co-assembed with *α*1- and *β*1-subunits [1205]); amiloride (selective blockade of receptors containing an *α*6-subunit [354]); furosemide (selective blockade of receptors containing an *α*6-subunit co-assembled with *β*2/*β*3-, but not *β*1-subunit [627]); La^3+^ (potentiates responses mediated by *α*1*β*3*γ*2L receptors, weakly inhibits *α*6*β*3*γ*2L receptors, and strongly blocks *α*6*β*3*δ* and *α*4*β*3*δ* receptors [134, 1070]); ethanol (selectively potentiates responses mediated by *α*4*β*3*δ* and *α*6*β*3*δ* receptors versus receptors in which *β*2 replaces *β*3, or *γ* replaces *δ* [1274], but see also [626]); DS1 and DS2 (selectively potentiate responses mediated by *δ*-subunit-containing receptors [1265]). It should be noted that the apparent selectivity of some positive allosteric modulators (*e.g.,* neurosteroids such as 5*α*-pregnan-3*α*-ol-20-one for *δ*-subunit-containing receptors (*e.g., α*1*β*3*δ*) may be a consequence of the unusually low efficacy of GABA at this receptor isoform [91, 101].

## Glycine receptors


Ion channels→Ligand-gated ion channels→Glycine receptors

### Overview:

The inhibitory glycine receptor (**nomenclature as agreed by the NC-IUPHAR Subcommittee on Glycine Receptors**) is a member of the Cys-loop superfamily of transmitter-gated ion channels that includes the GABA_A_, nicotinic acetylcholine and 5-HT_3_ receptors and Zn^2+^- activated channels. The glycine receptor is expressed either as a homo-pentamer of *α* subunits, or a complex of 4*α* and 1*β* subunits [1422], that contains an intrinsic anion channel. Four differentially expressed isoforms of the *α*-subunit (*α*1-*α*4) and one variant of the *β*-subunit (*β*1, *GLRB,* P48167) have been identified by genomic and cDNA cloning. Further diversity originates from alternative splicing of the primary gene transcripts for *α*1 (*α*1^INS^ and *α*1^del^), *α*2 (*α*2A and *α*2B), *α*3 (*α*3S and *α*3L) and *β* (*βΔ*7) subunits and by mRNA editing of the *α*2 and *α*3 subunit [318, 804, 901]. Both *α*2 splicing and *α*3 mRNA editing can produce subunits (*i.e., α*2B and *α*3P185L) with enhanced agonist sensitivity. Predominantly, the adult form of the receptor contains *α*1 (or *α*3) and *β* subunits whereas the immature form is mostly composed of only *α*2 subunits [774]. The *α*4 subunit is a pseudogene in humans [663]. High resolution molecular structures are available for *α*1 homomeric, *α*3 homomeric, and *αβ* hteromeric receptors in a variety of ligand-induced conformations [298, 298, 492, 493, 494, 1381]. As in other Cys-loop receptors, the orthosteric binding site for agonists and the competitive antagonist strychnine is formed at the interfaces between the subunits’ extracellular domains. Inclusion of the *β*-subunit in the pentameric glycine receptor contributes to agonist binding, reduces single channel conductance and alters pharmacology. The *β*-subunit also anchors the receptor, *via* an amphipathic sequence within the large intracellular loop region, to gephyrin. This a cytoskeletal attachment protein that binds to a number of subsynaptic proteins involved in cytoskeletal structure and thus clusters and anchors hetero-oligomeric receptors to the synapse [600, 838]. G protein *βγ* subunits enhance the open state probability of native and recombinant glycine receptors by association with domains within the large intracellular loop [1369, 1370]. Intracellular chloride concentration modulates the kinetics of native and recombinant glycine receptors [978]. Intracellular Ca^2+^ appears to increase native and recombinant glycine receptor affinity, prolonging channel open events, by a mechanism that does not involve phosphorylation [365]. Extracellular Zn^2+^ potentiates GlyR function at nanomolar concentrations [820]. and causes inhibition at higher micromolar concentrations (17).

**Table:** 

Nomenclature	glycine receptor *α*1 subunit	glycine receptor *α*2 subunit
HGNC, UniProt	*GLRA1,* P23415	*GLRA2,* P23416
Selective agonists (potency order)	glycine > *β*-alanine > taurine	glycine > *β*-alanine > taurine
Selective antagonists	ginkgolide X (pIC_50_ 6.1), pregnenolone sulphate (pK_i_ 5.7), nifedipine (pIC_50_ 5.5), bilobalide (pIC_50_ 4.7), tropisetron (pK_i_ 4.1), colchicine (pIC_50_ 3.5), PMBA, onternabez (weak inhibition), strychnine	HU-210 (pIC_50_ 7), WIN55212-2 (pIC_50_ 6.7), onternabez (pIC_50_ 6), ginkgolide X (pIC_50_ 5.6), pregnenolone sulphate (pK_i_ 5.3), bilobalide (pIC_50_ 5.1), tropisetron (pK_i_ 4.9), colchicine (pIC_50_ 4.2), 5,7-dichlorokynurenic acid (pIC_50_ 3.7), PMBA, strychnine
Channel blockers	ginkgolide B (pIC_50_ 5.1–6.2), cyanotriphenylborate (pIC_50_ 5.9) [1043], picrotin (pIC_50_ 5.3), picrotoxinin (pIC_50_ 5.3), picrotoxin (pIC_50_ 5.2)	picrotoxinin (pIC_50_ 6.4), picrotoxin (pIC_50_ 5.6), ginkgolide B (pIC_50_ 4.9–5.4), picrotin (pIC_50_ 4.9), cyanotriphenylborate (pIC_50_ >4.7) [1043]
Endogenous allosteric modulators	Zn^2+^ (Potentiation) (pEC_50_ 7.4), Cu^2+^ (Inhibition) (pIC_50_ 4.8–5.4), Zn^2+^ (Inhibition) (pIC_50_ 4.8), Extracellular H^+^ (Inhibition)	Zn^2+^ (Potentiation) (pEC_50_ 6.3), Cu^2+^ (Inhibition) (pIC_50_ 4.8), Zn^2+^ (Inhibition) (pIC_50_ 3.4)
Selective allosteric modulators	anandamide (Potentiation) (pEC_50_ 7.4), HU-210 (Potentiation) (pEC_50_ 6.6), Δ^9^-tetrahydrocannabinol (Potentiation) (pEC_50_ ~5.5)	Δ^9^-tetrahydrocannabinol (Potentiation) (pEC_50_ ~6)
Labelled ligands	[^3^H]strychnine (Antagonist)	[^3^H]strychnine (Antagonist)
Functional Characteristics	*γ* = 86 pS (main state); (+ *β* = 44 pS)	*γ* = 111 pS (main state); (+ *β* = 54 pS)

**Table:** 

Nomenclature	glycine receptor *α*3 subunit	glycine receptor *α*4 subunit (pseudogene in humans)	glycine receptor *β* subunit
HGNC, UniProt	*GLRA3,* O75311	*GLRA4,* Q5JXX5	*GLRB,* P48167
Selective agonists (potency order)	glycine > *β*-alanine > taurine	–	–
Selective antagonists	HU-210 (pIC_50_ 7.3), WIN55212-2 (pIC_50_ 7), onternabez (pIC_50_ 7), (12E,20Z,18S)-8-hydroxyvariabilin (pIC_50_ 5.2), nifedipine (pIC_50_ 4.5), strychnine		nifedipine (when co-expressed with the *α*1 subunit) (pIC_50_ 5.9), pregnenolone sulphate (when co-expressed with the *α*1 subunit) (pK_i_ 5.6), tropisetron (when co-expressed with the *α*2 subunit) (pK_i_ 5.3), pregnenolone sulphate (when co-expressed with the *α*2 subunit) (pK_i_ 5), nifedipine (when co-expressed with the *α*3 subunit) (pIC_50_ 4.9), bilobalide (when co-expressed with the *α*2 subunit) (pIC_50_ 4.3), bilobalide (when co-expressed with the *α*1 subunit) (pIC_50_ 3.7), ginkgolide X (when co-expressed with the *α*1 subunit) (pIC_50_ >3.5), ginkgolide X (when co-expressed with the *α*2 subunit) (pIC_50_ >3.5)
Channel blockers	picrotoxinin (pIC_50_ 6.4), ginkgolide B (pIC_50_ 5.7), picrotin (pIC_50_ 5.2), picrotoxin (block is weaker when *β* subunit is co-expressed)	–	ginkgolide B (when co-expressed with the *α*2 subunit) (pIC_50_ 6.1–6.9), ginkgolide B (when co-expressed with the *α*1 subunit) (pIC_50_ 5.6–6.7), ginkgolide B (when co-expressed with the *α*3 subunit) (pIC_50_ 6.3), cyanotriphenylborate (when co-expressed with the human *α*1 subunit) (pIC_50_ 5.6) [1043] – Rat, cyanotriphenylborate (when co-expressed with the human *α*2 subunit) (pIC_50_ 5.1) [1043] – Rat, picrotoxinin (when co-expressed with the *α*3 subunit) (pIC_50_ 5.1), picrotin (when co-expressed with the *α*1 subunit) (pIC_50_ 4.6), picrotin (when co-expressed with the *α*3 subunit) (pIC_50_ 4.6), picrotoxinin (when co-expressed with the *α*1 subunit) (pIC_50_ 4.6), picrotoxin (when co-expressed with the *α*2 subunit) (pIC_50_ 4.5), picrotoxin (when co-expressed with the *α*1 subunit) (pIC_50_ 3.7)
Endogenous allosteric modulators	Cu^2+^ (Inhibition) (pIC_50_ 5), Zn^2+^ (Inhibition) (pIC_50_ 3.8)	–	Zn^2+^ (Inhibition) (pIC_50_ 4.9), Zn^2+^ (Inhibition) (pIC_50_ 3.7)
Selective allosteric modulators	Δ^9^-tetrahydrocannabinol (Potentiation) (pEC_50_ ~5.3)	–	–
Labelled ligands	[^3^H]strychnine (Antagonist)	–	–
Functional Characteristics	*γ* = 105 pS (main state); (+ *β* = 48 pS)	–	–
Comments	–	–	Ligand interaction data for hetero-oligomer receptors containing the *β* subunit are also listed under the *α* subunit

### Comments:

Data in the table refer to homo-oligomeric assemblies of the *α*-subunit, significant changes introduced by co-expression of the *β*1 subunit are indicated in parenthesis. Not all glycine receptor ligands are listed within the table, but some that may be useful in distinguishing between glycine receptor isoforms are indicated (see detailed view pages for each subunit: *α*1, *α*2, *α*3, *α*4, *β* ). Pregnenolone sulphate, tropisetron and colchicine, for example, although not selective antagonists of glycine receptors, are included for this purpose. Strychnine is a potent and selective competitive glycine receptor antagonist with affinities in the range 5-15 nM. RU5135 demonstrates comparable potency, but additionally blocks GABA_A_ receptors. There are conflicting reports concerning the ability of cannabinoids to inhibit [736], or potentiate and at high concentrations activate [12, 267, 446, 1341, 1365] glycine receptors. Nonetheless, cannabinoid analogues may hold promise in distinguishing between glycine receptor subtypes [1365]. In addition, potentiation of glycine receptor activity by cannabinoids has been claimed to contribute to Cannabis-induced analgesia relying on Ser296/307 (*α*1/*α*3) in M3 [1341]. Several analogues of muscimol and piperidine act as agonists and antagonists of both glycine and GABA_A_receptors. Picrotoxin acts as an allosteric inhibitor that appears to bind within the pore, and shows strong selectivity towards homomeric receptors. While its components, picrotoxinin and picrotin, have equal potencies at *α*1 receptors, their potencies at *α*2 and *α*3 receptors differ modestly and may allow some distinction between different receptor types [1366]. Binding of picrotoxin within the pore has been demonstrated in the crystal structure of the related *C. elegans* GluCl Cys-loop receptor [457]. In addition to the compounds listed in the table, numerous agents act as allosteric regulators of glycine receptors (comprehensively reviewed in [661, 748, 1309, 1371]). Zn^2+^ acts through distinct binding sites of high- and low-affinity to allosterically enhance channel function at low (<10 *μ*M) concentrations and inhibits responses at higher concentrations in a subunit selective manner [819]. The effect of Zn^2+^ is somewhat mimicked by Ni^2+^. Endogenous Zn^2+^ is essential for normal glycinergic neurotransmission mediated by *α*1 subunit-containing receptors [467]. Elevation of intracellular Ca^2+^ produces fast potentiation of glycine receptor-mediated responses. Dideoxyforskolin (4 *μ*M) and tamoxifen (0.2–5 *μ*M) both potentiate responses to low glycine concentrations (15 *μ*M), but act as inhibitors at higher glycine concentrations (100 *μ*M). Additional modulatory agents that enhance glycine receptor function include inhalational, and several intravenous general anaesthetics (*e.g.* minaxolone, propofol and pentobarbitone) and certain neurosteroids. Ethanol and higher order n-alcohols also enhance glycine receptor function although whether this occurs by a direct allosteric action at the receptor [786], or through *βγ* subunits [1368] is debated. Recent crystal structures of the bacterial homologue, GLIC, have identified transmembrane binding pockets for both anaesthetics [896] and alcohols [486]. Solvents inhaled as drugs of abuse (*e.g.* toluene, 1-1-1-trichloroethane) may act at sites that overlap with those recognising alcohols and volatile anaesthetics to produce potentiation of glycine receptor function. The function of glycine receptors formed as homomeric complexes of *α*1 or *α*2 subunits, or hetero-oligomers of *α*1/*β* or *α*2/*β* subunits, is differentially affected by the 5-HT_3_ receptor antagonist tropisetron (ICS 205-930) which may evoke potentiation (which may occur within the femtomolar range at the homomeric glycine *α*1 receptor), or inhibition, depending upon the subunit composition of the receptor and the concentrations of the modulator and glycine employed. Potentiation and inhibition by tropeines involves different binding modes [770]. Additional tropeines, including atropine, modulate glycine receptor activity.

## Ionotropic glutamate receptors


Ion channels→Ligand-gated ion channels→Ionotropic glutamate receptors

### Overview:

The ionotropic glutamate receptors comprise members of the NMDA (N-methyl-D-aspartate), AMPA (*α*-amino-3-hydroxy-5-methyl-4-isoxazoleproprionic acid) and kainate receptor classes, named originally according to their preferred, synthetic, agonist [280, 728, 1225]. Receptor heterogeneity within each class arises from the homo-oligomeric, or hetero-oligomeric, assembly of distinct subunits into cation-selective tetramers. Each subunit of the tetrameric complex comprises an extracellular amino terminal domain (ATD), an extracellular ligand binding domain (LBD), 3 TM domains (M1, M3 and M4), a channel lining re-entrant’p-loop’ (M2) located between M1 and M3 and an intracellular carboxy- terminal domain (CTD) [557, 647, 794, 857, 1225]. The X-ray structure of a homomeric ionotropic glutamate receptor (GluA2- see below) has recently been solved at 3.6 Å resolution [1130] and although providing the most complete structural information current available may not be representative of the subunit arrangement of, for example, the heteromeric NMDA receptors [570]. It is beyond the scope of this supplement to discuss the pharmacology of individual ion-otropic glutamate receptor isoforms in detail; such information can be gleaned from [184, 227, 280, 325, 525, 526, 588, 927, 928, 929, 1225, 1337]. Agents that discriminate between subunit isoforms are, where appropriate, noted in the tables and additional compounds that distinguish between receptor isoforms are indicated in the text below.

### The classification of glutamate receptor subunits has been re-addressed by NC-IUPHAR [220].

The scheme developed recommends a nomenclature for ionotropic glutamate receptor subunits that is adopted here.

### NMDA receptors

NMDA receptors assemble as obligate heteromers that may be drawn from GluN1, GluN2A, GluN2B, GluN2C, GluN2D, GluN3A and GluN3B subunits. Alternative splicing can generate eight isoforms of GluN1 with differing pharmacological properties. Various splice variants of GluN2B, 2C, 2D and GluN3A have also been reported. Activation of NMDA receptors containing GluN1 and GluN2 subunits requires the binding of two agonists, glutamate to the S1 and S2 regions of the GluN2 subunit and glycine to S1 and S2 regions of the GluN1 subunit [185, 324]. The minimal requirement for efficient functional expression of NMDA receptors *in vitro* is a di-heteromeric assembly of GluN1 and at least one GluN2 subunit variant, as a dimer of heterodimers arrangement in the extracellular domain [367, 570, 794]. However, more complex tri-heteromeric assemblies, incorporating multiple subtypes of GluN2 subunit, or GluN3 subunits, can be generated *in vitro* and occur *in vivo*. The NMDA receptor channel commonly has a high relative permeability to Ca^2+^ and is blocked, in a voltage-dependent manner, by Mg^2+^ such that at resting potentials the response is substantially inhibited.

### AMPA and Kainate receptors

AMPA receptors assemble as homomers, or heteromers, that may be drawn from GluA1, GluA2, GluA3 and GluA4 subunits. Transmembrane AMPA receptor regulatory proteins (TARPs) of class I (i.e. *γ*2, *γ*3, *γ*4 and *γ*8) act, with variable stoichiometry, as auxiliary subunits to AMPA receptors and influence their trafficking, single channel conductance gating and pharmacology (reviewed in [330, 517, 822, 1216]). Functional kainate receptors can be expressed as homomers of GluK1, GluK2 or GluK3 subunits. GluK1-3 subunits are also capable of assembling into heterotetramers (*e.g.* GluK1/K2; [681, 960, 974]). Two additional kainate receptor subunits, GluK4 and GluK5, when expressed individually, form high affinity binding sites for kainate, but lack function, but can form heteromers when expressed with GluK1-3 subunits (*e.g.* GluK2/K5; reviewed in [525, 960, 974]). Kainate receptors may also exhibit‘metabotropic’ functions [681, 1030]. As found for AMPA receptors, kainate receptors are modulated by auxiliary subunits (Neto proteins, [682, 960]). An important function difference between AMPA and kainate receptors is that the latter require extracellular Na^+^ and Cl^−^ for their activation [125, 985]. RNA encoding the GluA2 subunit undergoes extensive RNA editing in which the codon encoding a p-loop glutamine residue (Q) is converted to one encoding arginine (R). This Q/R site strongly influences the biophysical properties of the receptor. Recombinant AMPA receptors lacking RNA edited GluA2 subunits are: (1) permeable to Ca^2+^; (2) blocked by intracellular polyamines at depolarized potentials causing inward rectification (the latter being reduced by TARPs); (3) blocked by extracellular argiotoxin and joro spider toxins and (4) demonstrate higher channel conductances than receptors containing the edited form of GluA2 [508, 1087]. GluK1 and GluK2, but not other kainate receptor subunits, are similarly edited and broadly similar functional characteristics apply to kainate receptors lacking either an RNA edited GluK1, or GluK2, subunit [681, 960]. Native AMPA and kainate receptors displaying differential channel conductances, Ca^2+^ permeabilites and sensitivity to block by intracellular polyamines have been identified [226, 508, 721]. GluA1-4 can exist as two variants generated by alternative splicing (termed ‘flip’ and ‘flop’) that differ in their desensitization kinetics and their desensitization in the presence of cyclothiazide which stabilises the nondesensitized state. TARPs also stabilise the non-desensitized conformation of AMPA receptors and facilitate the action of cyclothiazide [822]. Splice variants of GluK1-3 also exist which affects their trafficking [681, 960].

**Table:** 

Nomenclature	GluA1	GluA2	GluA3	GluA4
HGNC, UniProt	*GRIA1*, P42261	*GRIA2,* P42262	*GRIA3,* P42263	*GRIA4,* P48058
Agonists	(S)-5-fluorowillardiine, AMPA	(S)-5-fluorowillardiine, AMPA	(S)-5-fluorowillardiine, AMPA	(S)-5-fluorowillardiine, AMPA
Selective antagonists	ATPO, GYKI53655, GYKI53784 (active isomer, non-competitive), NBQX, tezampanel			
Channel blockers	extracellular argiotoxin, extracellular joro spider toxin (selective for channels lacking GluA2)	extracellular argiotoxin	extracellular argiotoxin, extracellular joro spider toxin (selective for channels lacking GluA2)	extracellular argiotoxin, extracellular joro spider toxin (selective for channels lacking GluA2)
Allosteric modulators (Positive)	LY392098 (pEC_50_ 5.8) [827], LY404187 (pEC_50_ 5.2) [827], cyclothiazide (pEC_50_ 4.7) [827], CX516, CX546, IDRA-21, LY503430, S18986, aniracetam, piracetam	LY404187 (pEC_50_ 6.8) [827], LY392098 (pEC_50_ 6.7) [827], cyclothiazide (pEC_50_ 5.7) [827], CX516, CX546, IDRA-21, LY503430, S18986, aniracetam, piracetam	LY404187 (pEC_50_ 5.8) [827], LY392098 (pEC_50_ 5.7) [827], cyclothiazide (pEC_50_ 4.9) [827], CX516, CX546, IDRA-21, LY503430, S18986, aniracetam, piracetam	LY392098 (pEC_50_ 6.7) [827], LY404187 (pEC_50_ 6.7) [827], cyclothiazide (pEC_50_ 5.4) [827], CX516, CX546, IDRA-21, LY503430, S18986, aniracetam, piracetam
Labelled ligands	[^3^H]AMPA (Agonist), [^3^H]CNQX (Antagonist)	[^3^H]AMPA (Agonist), [^3^H]CNQX (Antagonist)	[^3^H]AMPA, [^3^H]CNQX	[^3^H]AMPA (Agonist), [^3^H]CNQX
Comments	Piracetam and aniracetam are examples of pyrrolidinones. Cyclothiazide, S18986, and IDRA-21 are examples of benzothiadiazides. CX516 and CX546 are examples of benzylpiperidines. LY392098, LY404187 and LY503430 are examples of biarylpropylsulfonamides. Also blocked by intracellular polyamines.			

**Table:** 

Nomenclature	GluD1	GluD2
HGNC, UniProt	*GRID1*, Q9ULKO	*GRID2,* O43424

**Table:** 

Nomenclature	GluK1	GluK2	GluK3	GluK4	GluK5
HGNC, UniProt	*GRIK1,* P39086	*GRIK2,* Q13002	*GRIK3,* Q13003	*GRIK4,* Q16099	*GRIK5,* Q16478
Agonists	dysiherbaine [1054] – Rat, SYM2081 [951], kainate [1119], (S)-4-AHCP, (S)-5-iodowillardiine, 8-deoxy-neodysiherbaine, ATPA, domoic acid	dysiherbaine [1054] – Rat, domoic acid [152], SYM2081 [1417] – Rat, kainate [152, 1119]	SYM2081 [1052] – Rat, kainate (low potency) [1052] – Rat, dysiherbaine	SYM2081, domoic acid, dysiherbaine, kainate	SYM2081, domoic acid, dysiherbaine, kainate
Selective agonists	LY339434 [1119]	–	–	–	–
Selective antagonists	2,4-epi-neodysiherbaine, ACET, LY382884, LY466195, MSVIII-19, NS3763 (non-competitive), UBP302, UBP310	2,4-epi-neodysiherbaine	–	–	–
Allosteric modulators (Positive)	concanavalin A	concanavalin A	–	–	–
Labelled ligands	[^3^H]UBP310 (Antagonist) (pK_d_ 7.7) [47], [^3^H]SYM2081 (Agonist), [^3^H]kainate (Agonist)	[^3^H]kainate (Agonist) [1417]–Rat, [^3^H]SYM2081 (Agonist)	[^3^H]UBP310 (Antagonist) (pK_d_ 6.3) [47], [^3^H]SYM2081 (Agonist), [^3^H]kainate (Agonist)	[^3^H]SYM2081 (Agonist), [^3^H]kainate (Agonist)	[^3^H]SYM2081 (Agonist), [^3^H]kainate (Agonist)
Comments	–	Intracellular polyamines are subtype selective channel blockers (GluK3 ≫ GluK2)	Domoic acid and concanavalin A are inactive at the GluK3 subunit. Intracellular polyamines are subtype selective channel blockers (GluK3 ≫ GluK2)	–	–

**Table:** 

Nomenclature	GluN1
HGNC, UniProt	*GRIN1,* Q05586
Endogenous agonists	D-aspartic acid [glutamate site], D-serine [glycine site], L-aspartic acid [glutamate site], glycine [glycine site]
Agonists	(+)-HA966 [glycine site] (Partial agonist), (RS)-(tetrazol-5-yl)glycine [glutamate site], NMDA [glutamate site], homoquinolinic acid [glutamate site] (Partial agonist)
Selective antagonists	L701324 [glycine site] (pIC_50_ 8.7) [646] – Rat, GV196771A [glycine site] (pK_i_ 8.1–8.4) [201] – Rat, L689560 [glycine site] (pIC_50_ 8.1) [675] – Rat, 5,7-dichlorokynurenic acid [glycine site]
Labelled ligands	[^3^H]MDL105519 [glycine site] (Antagonist) (pK_d_ ~8.5) [178] – Rat, [^3^H]CGP39653 [glutamate site] (Selective Antagonist), [^3^H]CGP61594 [glycine site] (Antagonist), [^3^H]CGS19755 [glutamate site] (Antagonist), [^3^H]CPP [glutamate site] (Selective Antagonist), [^3^H]L689560 [glycine site] (Antagonist), [^3^H]dizocilpine [cation channel] (Antagonist), [^3^H]glycine [glycine site] (Agonist)

**Table:** 

Nomenclature	GluN2A	GluN2B	GluN2C	GluN2D
HGNC, UniProt	*GRIN2A,* Q12879	*GRIN2B,* Q13224	*GRIN2C,* Q14957	*GRIN2D,* O15399
Endogenous agonists	D-aspartic acid [glutamate site] (GluN2D > GluN2C = GluN2B > GluN2A), D-serine [glycine site] (GluN2D > GluN2C > GluN2B > GluN2A), L-aspartic acid [glutamate site] (GluN2D = GluN2B > GluN2C = GluN2A), glycine [glycine site] (GluN2D > GluN2C > GluN2B > GluN2A)			
Agonists	(+)-HA966 [glycine site] (Partial agonist), (RS)-(tetrazol-5-yl)glycine [glutamate site] (GluN2D > GluN2C = GluN2B > GluN2A), NMDA [glutamate site] (GluN2D > GluN2C > GluN2B > GluN2A), homoquinolinic acid [glutamate site] (GluN2B ≥ GluN2A ≥ GluN2D > GluN2C; partial agonist at GluN2A and GluN2C)		(RS)-(tetrazol-5-yl)glycine [glutamate site](GluN2D > GluN2C = GluN2B > GluN2A), NMDA [glutamate site] (GluN2D > GluN2C > GluN2B > GluN2A), homoquinolinic acid [glutamate site] (GluN2B ≥ GluN2A ≥ GluN2D > GluN2C; partial agonist at GluN2A and GluN2C)	
Selective antagonists	5,7-dichlorokynurenic acid [glycine site], CGP37849 [glutamate site], GV196771A [glycine site], L689560 [glycine site], L701324 [glycine site], LY233053 [glutamate site], NVP-AAM077 [glutamate site] (GluN2A > GluN2B (human), but weakly selective for rat GluN2A versus GluN2B) [49, 345, 363, 868], UBP141 [glutamate site] (GluN2D ≥ GluN2C > GluN2A ≥ GluN2B) [837], conantokin-G [glutamate site] (GluN2B > GluN2D = GluN2C = GluN2A), d-AP5 [glutamate site], d-CCPene [glutamate site] (GluN2A = GluN2B > GluN2C = GluN2D), selfotel [glutamate site]		5,7-dichlorokynurenic acid [glycine site], CGP37849 [glutamate site], GV196771A [glycine site], L689560 [glycine site], L701324 [glycine site], LY233053 [glutamate site], UBP141 [glutamate site] (GluN2D ≥ GluN2C > GluN2A ≥ GluN2B) [837], conantokin-G [glutamate site] (GluN2B > GluN2D = GluN2C = GluN2A), d-AP5 [glutamate site], d-CCPene [glutamate site] (GluN2A = GluN2B > GluN2C = GluN2D), selfotel [glutamate site]	
Channel blockers	Mg^2+^ (GluN2A = GluN2B > GluN2C = GluN2D), N^1^-dansyl-spermine (GluN2A = GluN2B ≫ GluN2C = GluN2D), amantadine (GluN2C = GluN2D ≥ GluN2B ≥ GluN2A), dizocilpine, ketamine, phencyclidine		phencyclidine (pIC_50_ 7.1) [296], ketamine (pIC_50_ 6.2) [296], amantadine (GluN2C = GluN2D ≥ GluN2B ≥ GluN2A) (pIC_50_ 4.7) [296], Mg^2+^ (GluN2A = GluN2B > GluN2C = GluN2D), N^1^-dansyl-spermine (GluN2A = GluN2B ≫ GluN2C = GluN2D), dizocilpine	Mg^2+^ (GluN2A = GluN2B > GluN2C = GluN2D), N^1^-dansyl-spermine (GluN2A = GluN2B ≫ GluN2C = GluN2D), amantadine (GluN2C = GluN2D ≥ GluN2B ≥ GluN2A), dizocilpine, ketamine, phencyclidine
Labelled ligands	[^3^H]CGP39653 [glutamate site] (Antagonist), [^3^H]CGP61594 [glycine site] (Antagonist), [^3^H]CGS19755 [glutamate site] (Antagonist), [^3^H]CPP [glutamate site] (Antagonist), [^3^H]L689560 [glycine site] (Antagonist), [^3^H]MDL105519 [glycine site] (Antagonist), [^3^H]dizocilpine [cation channel] (Channel blocker), [^3^H]glycine [glycine site] (Agonist)			

**Table:** 

Nomenclature	GluN3A	GluN3B
HGNC, UniProt	*GRIN3A,* Q8TCU5	*GRIN3B,* O60391
Comments	See the main comments section below for information on the pharmacology of GluN3A and GluN3B subunits	

### Comments: NMDA receptors

Potency orders unreferenced in the table are from [184, 296, 325, 648, 929, 1225]. In addition to the glutamate and glycine binding sites documented in the table, physiologically important inhibitory modulatory sites exist for Mg^2+^, Zn^2+^, and protons [227, 280, 1225]. Voltage-independent inhibition by Zn^2+^ binding with high affinity within the ATD is highly subunit selective (GluN2A ≫ GluN2B > GluN2C ≥ GluN2D; [929, 1225]). The receptor is also allosterically modulated, in both positive and negative directions, by endogenous neuroactive steroids in a subunit dependent manner [482, 771]. Tonic proton blockade of NMDA receptor function is alleviated by polyamines and the inclusion of exon 5 within GluN1 subunit splice variants, whereas the non-competitive antagonists ifenprodil and traxoprodil increase the fraction of receptors blocked by protons at ambient concentration. Inclusion of exon 5 also abolishes potentiation by polyamines and inhibition by Zn^2+^ that occurs through binding in the ATD [1224]. Ifenprodil, traxoprodil, haloperidol, felbamate and Ro 8-4304 discriminate between recombinant NMDA receptors assembled from GluN1 and either GluN2A, or GluN2B, subunits by acting as selective, non-competitive, antagonists of heterooligomers incorporating GluN2B through a binding site at the ATD GluN1/GluN2B subunit interface [570]. LY233536 is a competitive antagonist that also displays selectivity for GluN2B over GluN2A subunit-containing receptors. Similarly, CGP61594 is a photoaffinity label that interacts selectively with receptors incorporating GluN2B versus GluN2A, GluN2D and, to a lesser extent, GluN2C subunits. TCN-201 and TCN-213 have been shown to block GluN2A NMDA receptors selectively by a mechanism that involves allosteric inhibition of glycine binding to the GluN1 site [97, 315, 429, 799]. In addition to influencing the pharmacological profile of the NMDA receptor, the identity of the GluN2 subunit co-assembled with GluN1 is an important determinant of biophysical properties that include sensitivity to block by Mg^2+^, single-channel conductance and maximal open probability and channel deactivation time [227, 324, 386]. Incorporation of the GluN3A subunit into tri-heteromers containing GluN1 and GluN2 subunits is associated with decreased single-channel conductance, reduced permeability to Ca^2+^ and decreased susceptibility to block by Mg^2+^ [164, 451]. Reduced permeability to Ca^2+^ has also been observed following the inclusion of GluN3B in tri-heteromers. The expression of GluN3A, or GluN3B, with GluN1 alone forms, in *Xenopus laevis* oocytes, a cation channel with unique properties that include activation by glycine (but not NMDA), lack of permeation by Ca^2+^ and resistance to blockade by Mg^2+^ and NMDA receptor antagonists [173]. The function of heteromers composed of GluN1 and GluN3A is enhanced by Zn^2+^, or glycine site antagonists, binding to the GluN1 subunit [758]. Zn^2+^ also directly activates such complexes. The co-expression of GluN1, GluN3A and GluN3B appears to be required to form glycine-activated receptors in mammalian cell hosts [1129].

### AMPA and Kainate receptors

All AMPA receptors are additionally activated by kainate (and domoic acid) with relatively low potency, (EC_50_ ~ 100 *μ*M). Inclusion of TARPs within the receptor complex increases the potency and maximal effect of kainate [517, 822]. AMPA is weak partial agonist at GluK1 and at heteromeric assemblies of GluK1/GluK2, GluK1/GluK5 and GluK2/GluK5 [525]. Quinoxalinediones such as CNQX and NBQX show limited selectivity between AMPA and kainate receptors. Tezampanel also has kainate (GluK1) receptor activity as has GYKI53655 (GluK3 and GluK2/GluK3) [525]. ATPO is a potent competitive antagonist of AMPA receptors, has a weaker antagonist action at kainate receptors comprising GluK1 subunits, but is devoid of activity at kainate receptors formed from GluK2 or GluK2/GluK5 subunits. The pharmacological activity of ATPO resides with the (S)-enantiomer. ACET and UBP310 may block GluK3, in addition to GluK1 [47, 959]. (2S,4R)-4-methylglutamate (SYM2081) is equipotent in activating (and desensitising) GluK1 and GluK2 receptor isoforms and, *via* the induction of desensitisation at low concentrations, has been used as a functional antagonist of kainate receptors. Both (2*S*,4*R*)-4-methylglutamate and LY339434 have agonist activity at NMDA receptors. (2S,4R)-4-methylglutamate is also an inhibitor of the glutamate transporters EAAT1 and EAAT2.

### Delta subunits

GluD1 and GluD2 comprise, on the basis of sequence homology, an ‘orphan’ class of ionotropic glutamate receptor subunit. They do not form a functional receptor when expressed solely, or in combination with other ionotropic glutamate receptor subunits, in transfected cells [1390]. However, GluD2 subunits bind D-serine and glycine and GluD2 subunits carrying the mutation A654T form a spontaneously open channel that is closed by D-serine [861].

## IP_3_ receptors


$$\text{Ion channels}\to \text{Ligand-gated ion channels}\to {\text{IP}}_{3}\;\text{receptors}$$

### Overview:

The inositol 1,4,5-trisphosphate receptors (IP_3_R) are ligand-gated Ca^2+^-release channels on intracellular Ca^2+^ store sites (such as the endoplasmic reticulum). They are responsible for the mobilization of intracellular Ca^2+^ stores and play an important role in intracellular Ca^2+^ signalling in a wide variety of cell types. Three different gene products (types I-III) have been isolated, which assemble as large tetrameric structures. IP_3_Rs are closely associated with certain proteins: calmodulin (*CALM1 CALM2 CALM3,* P62158) and FKBP (and calcineurin *via* FKBP). They are phosphorylated by PKA, PKC, PKG and CaMKII.

**Table:** 

Nomenclature	IP_3_R1	IP_3_R2	IP_3_R3
HGNC, UniProt	*ITPR1,* Q14643	*ITPR2,* Q14571	*ITPR3,* Q14573
Endogenous activators	cytosolic ATP (< mM range), cytosolic Ca^2+^ Concentration range: <7.5×10^−4^M, IP_3_ (endogenous; nM - *μ*M range)	cytosolic Ca^2+^ (nM range), IP_3_ (endogenous; nM - *μ*M range)	cytosolic Ca^2+^ (nM range), IP_3_ (endogenous; nM - *μ* M range)
Activators	adenophostin A (pharmacological; nM range), inositol 2,4,5-trisphosphate (pharmacological; also activated by other InsP_3_ analogues)	adenophostin A (pharmacological; nM range), inositol 2,4,5-trisphosphate (pharmacological; also activated by other InsP_3_ analogues)	–
Antagonists	PIP_2_ (*μ*M range), caffeine (mM range), decavanadate (*μ*M range), xestospongin C (*μ*M range)	decavanadate (*μ*M range)	decavanadate (*μ*M range)
Functional Characteristics	Ca^2+^: (P_Ba_/P_K_ ~6) single-channel conductance ~70 pS (50 mM Ca^2+^)	Ca^2+^: single-channel conductance ~70 pS (50 mM Ca^2+^) ~390 pS (220 mM Cs^+^)	Ca^2+^: single-channel conductance ~88 pS (55 mM Ba^2+^)
Comments	IP_3_ R1 is also antagonised by calmodulin at high cytosolic Ca^2+^ concentrations	–	–

### Comments:

The absence of a modulator of a particular isoform of receptor indicates that the action of that modulator has not been determined, not that it is without effect.

## Nicotinic acetylcholine receptors (nACh)


Ion channels→Ligand-gated ion channels→Nicotinic acetylcholine receptors (nACh)

### Overview:

Nicotinic acetylcholine (ACh) receptors are members of the Cys-loop family of transmitter-gated ion channels that includes the GABA_A_, strychnine-sensitive glycine and 5-HT_3_ receptors [21, 817, 1113, 1185, 1334]. All nicotinic receptors are pentamers in which each of the five subunits contains 4 TM domains. Genes encoding a total of 17 subunits (*α*1-10, *β* 1-4, *γ*, *δ* and *ϵ*) have been identified [560]. All subunits with the exception of *α*8 (present in avian species) have been identified in mammals. All *α* subunits possess two tandem cysteine residues near to the site involved in acetylcholine binding, and subunits not named *α* lack these residues [817]. The orthosteric ligand binding site is formed by residues within at least three peptide domains on the *α* subunit (principal component), and three on the adjacent subunit (complementary component). Nicotinic ACh receptors contain several allosteric modulatory sites. One such site, for positive allosteric modulators (PAMs) and allosteric agonists, has been proposed to reside within an intrasubunit cavity between the 4 TM domains [387, 1377]; see also [457]). The high resolution crystal structure of the molluscan ACh binding protein, a structural homologue of the extracellular binding domain of a nicotinic receptor pentamer, in complex with several nicotinic receptor ligands (*e.g.*[166]) and the crystal structure of the extracellular domain of the *α*1 subunit bound to *α*-bungarotoxin at 1.94 Â resolution [262], has revealed the orthosteric binding site in detail (reviewed in [171, 560, 1041, 1113]). Nicotinic receptors at the somatic neuromuscular junction of adult animals have the stoichiometry (*α*1)_2*β*_1*δϵ*, whereas an extrajunctional (*α*1)_2*β*_1*γδ* receptor predominates in embryonic and denervated skeletal muscle and other pathological states. Other nicotinic receptors are assembled as combinations of *α* (2-6) and *β*(2-4) subunits. For *α*1, *α*3, *α*4 and *β*2 and *β*4 subunits, pairwise combinations of *α* and *β* (e.g. *α*3*β*4 and *α*4*β*2) are sufficient to form a functional receptor *in vitro,* but far more complex isoforms may exist *in vivo* (reviewed in [395, 396, 817]). There is strong evidence that the pairwise assembly of some *α* and *β* subunits can occur with variable stoichiometry [*e.g.* (*α*4)_2_(*β*2)_2_ or (*α*4)_3_(*β*2)_2_] which influences the biophysical and pharmacological properties of the receptor [817]. *α*5 and *β*3 subunits lack function when expressed alone, or pairwise, but participate in the formation of functional hetero-oligomeric receptors when expressed as a third subunit with another *α* and *β* pair [e.g. *α*4*α*5*αβ*2, *α*4*αβ*2*β*3, *α*5*α*6*β*2, see [817] for further examples]. The *α*6 subunit can form a functional receptor when co-expressed with *β*4 *in vitro*, but more efficient expression ensues from incorporation of a third partner, such as *β*3 [1361]. The *α*7, *α*8, and *α*9 subunits form functional homo-oligomers, but can also combine with a second subunit to constitute a hetero-oligomeric assembly (*e.g. α*7*β*2 and *α*9*α*10). For functional expression of the *α*10 subunit, co-assembly with *α*9 is necessary. The latter, along with the *α*10 subunit, appears to be largely confined to cochlear and vestibular hair cells. Comprehensive listings of nicotinic receptor subunit combinations identified from recombinant expression systems, or *in vivo,* are given in [817]. In addition, numerous proteins interact with nicotinic ACh receptors modifying their assembly, trafficking to and from the cell surface, and activation by ACh (reviewed by [39, 550, 816]).

The nicotinic receptor Subcommittee of **NC-IUPHAR** has recommended a nomenclature and classification scheme for nicotinic acetylcholine (nACh) receptors based on the subunit composition of known, naturally- and/or heterologously-expressed nACh receptor subtypes [742]. Headings for this table reflect abbreviations designating nACh receptor subtypes based on the predominant *α* subunit contained in that receptor subtype. An asterisk following the indicated *α* subunit denotes that other subunits are known to, or may, assemble with the indicated *α* subunit to form the designated nACh receptor subtype(s). Where subunit stoichiometries within a specific nACh receptor subtype are known, numbers of a particular subunit larger than 1 are indicated by a subscript following the subunit (enclosed in parentheses- see also [220]).

**Table:** 

Nomenclature	nicotinic acetylcholine receptor *α*1 subunit	nicotinic acetylcholine receptor *α*2 subunit	nicotinic acetylcholine receptor *α*3 subunit	nicotinic acetylcholine receptor *α*4 subunit
HGNC, UniProt	*CHRNA1,* P02708	*CHRNA2,* Q15822	*CHRNA3,* P32297	*CHRNA4,* P43681
Commonly used antagonists	(*α*1)_2*β*_1*γδ* and (*α*1)_2*β*_1*δϵ*: *α*-bungarotoxin > pancuronium > vecuronium > rocuronium > tubocurarine (IC_50_ = 43-82 nM)	*α*2*β*2: DH*β*E (*K_B_* = 0.9 *μ*M), tubocurarine (*K_B_* = 1.4 *μ*M); *α*2*β*4: DH*β*E (*K_B_* = 3.6 *μ*M), tubocurarine (*K_B_* = 4.2 *μ*M)	*α*3*β*2: DH*β*E (*K_B_* = 1.6 *μ*M, IC_50_ = 2.0 *μ*M), tubocurarine (*K_B_* = 2.4 *μ*M); *α*3*β*4: DH*β*E (*K_B_* = 19 *μ*M, IC_50_ = 26 *μ*M), tubocurarine (*K_B_* = 2.2 *μ*M)	*α*4*β*2: DH*β*E (*K*_*B*_ = 0.1 *μ*M; IC_50_ = 0.08-0.9 *μ*M), tubocurarine (*K_B_* = 3.2 *μ*M, IC_50_ = 34 *μ*M); *α*4*β*4: DH*β*E (*K_B_* = 0.01 *μM*, IC_50_ = 0.19-1.2 *μ*M), tubocurarine (*K_B_* = 0.2 *μM*, IC_50_ = 50 *μ*M)
Selective agonists	succinylcholine (selective for (*α*1)_2*β*_1*γδ*)	–	–	varenicline [215], rivanicline [286], TC-2559 (*α*4*β*2) [193]
Selective antagonists	*α*-bungarotoxin, *α*-conotoxin GI, *α*-conotoxin MI, pancuronium, waglerin-1 (selective for (*α*1)_2*β*_1*δϵ*)	–	*α*-conotoxin AuIB (*α*3*β*4), *α*-conotoxin MII (*α*3*β*2), *α*-conotoxin PnIA (*α*3*β*2), *α*-conotoxin TxIA (*α*3*β*2), *α*-conotoxin-GIC (*α*3*β*2)	–
Channel blockers	gallamine ((*α*1)_2*β*_1*γδ* and (*α*1)_2*β*_1*δϵ*) (pIC_50_ ~6), mecamylamine ((*α*1)_2*β*_1*δϵ*)(pIC_50_ ~5.8)	hexamethonium, mecamylamine	mecamylamine (*α*3*β*4) (pIC_50_ 6.4), mecamylamine (*α*3*β*2) (pIC_50_ 5.1), A-867744 (*α*3*β*4) [775], NS1738 (*α*3*β*4) [1210], hexamethonium (*α*3*β*4), hexamethonium (*α*3*β*2)	mecamylamine (*α*4*β*4) (pIC_50_ 5.3–6.5), mecamylamine (*α*4*β*2) (pIC_50_ 5.4–5.4), hexamethonium (*α*4*β*2) (pIC_50_ 4.5–5.2), hexamethonium (*α*4*β*4) (pIC_50_ 4), A-867744 (*α*4*β*2) [775], NS1738 (*α*4*β*2) [1210]
Allosteric modulators (Positive)	–	LY2087101 [129]	–	LY2087101 (potentiates *α*4*β*2 and *α*4*β*4) [129]
Selective allosteric modulators	–	–	–	NS9283 (Positive) [667]
Labelled ligands	[^125^I]*α*-bungarotoxin (Selective Antagonist), [^3^H]*α*-bungarotoxin (Selective Antagonist)	[^125^I]epibatidine (Agonist), [^3^H] epibatidine (Agonist), [^3^H]cytisine (Agonist)	[^125^I]epibatidine (Agonist), [^3^H]epibatidine (Agonist), [^125^I]epibatidine (Agonist), [^3^H]epibatidine (Agonist), [^3^H]cytisine (Agonist)	[^125^I]epibatidine (Agonist), [^3^H]epibatidine (Agonist), [^3^H]cytisine (Agonist), [^125^I]epibatidine (Agonist), [^3^H]epibatidine (Agonist), [^3^H]epibatidine (Agonist) – Rat, [^3^H]cytisine (Agonist)
Functional Characteristics	(*α*1)_2*βγδ*_: P_Ca_/P_Na_ = 0.16-0.2, *P*_f_ = 2.1-2.9%; (*α*1)_2*βδϵ*_: P_Ca_/P_Na_ = 0.65-1.38, *P*_f_ = 4.1-7.2%	*α*2*β*2: P_Ca_/P_Na_ ~ 1.5	*α*3*β*2: P_Ca_/P_Na_ = 1.5; *α*3*β*4: P_Ca_/P_Na_ = 0.78-1.1, *P*_f_ = 2.7-4.6%	*α*4*β*2: P_Ca_/P_Na_ = 1.65, *P*_f_ = 2.6-2.9%; *α*4*β*4: *P*_f_ = 1.5-3.0%

**Table:** 

Nomenclature	nicotinic acetylcholine receptor *α*5 subunit	nicotinic acetylcholine receptor *α*6 subunit	nicotinic acetylcholine receptor *α*7 subunit
HGNC, UniProt	*CHRNA5,* P30532	*CHRNA6,* Q15825	*CHRNA7,* P36544
Commonly used antagonists	–	*α*6/*α*3*β*2*β*3 chimera: DH*β*E (IC_50_ = 1.1 *μ*M)	(*α*7)_5_: DH*β*E (IC_50_ = 8-20 *μ*M); (*α*7)_5_: tubocurarine (IC_50_ = 3.1 *μ*M)
Selective agonists	–	–	encenicline (Partial agonist) [795, 889], AQW051 ([125I]*α*- bungarotoxin binding assay) [501], 4BP-TQS (allosteric) [387], A-582941 ((*α*7)_5_) [105], PHA-543613 ((*α*7)_5_) [1324], PHA-709829 ((*α*7)_5_) [9], PNU-282987 ((*α*7)_5_) [112], bradanicline ((*α*7)_5_) [442]
Selective antagonists	*α*-conotoxin MII, *α*-conotoxin PnIA, *α*-conotoxin TxIA, *α*-conotoxin-GIC	*α*-conotoxin MII (*α*6*β*2*), *α*-conotoxin MII [H9A, L15A] (*α*6*β*2*β*3), *α*-conotoxin PIA (*α*6/*α*3*β*2*β*3 chimera)	*α*-bungarotoxin ((*α*7)_5_), *α*-conotoxin ArIB ((*α*7)_5_), *α*-conotoxin ImI ((*α*7)_5_), methyllycaconitine ((*α*7)_5_)
Channel blockers	–	mecamylamine (*α*6/*α*3*β*2*β*3 chimera) (pIC_50_ 5), hexamethonium (*α*6/*α*3*β*2*β*3 chimera) (pIC_50_ 4)	mecamylamine ((*α*7)_5_) (pIC_50_ 4.8)
Allosteric modulators (Positive)	–	–	A-867744 ((*α*7)_5_:Type 2; also blocks *α*3*β*4 and *α*4*β*2) [775], LY2087101 ((*α*7)_5_:Type 1) [129], NS1738 ((*α*7)_5_:Type 1; also blocks *α*3*β*4 and *α*4*β*2) [1210]
Selective allosteric modulators	–	–	BNC375 (Positive) (pEC_50_ 5.6) [1293], JNJ1930942 (Positive) [281], PNU-120596 (Positive) [500]
Labelled ligands	–	[^3^H]epibatidine (Agonist) – Chicken, [^125^I]*α*-conotoxin MII (Antagonist)	[^3^H]epibatidine (Agonist), [^3^H]A-585539 (Agonist) [31], [^3^H]AZ11637326 (Agonist) [394], [^125^I]*α*-bungarotoxin (Selective Antagonist) (pK_d_ 8.3–9.1), [^3^H]*α*-bungarotoxin (Selective Antagonist) (pK_d_ 8.3–9.1), [^3^H]methyllycaconitine (Antagonist) (pK_d_ 8.7) – Rat
Functional Characteristics	–	–	P_Ca_/^P^Na = 6.6-20, *P*_f_ = 8.8-11.4%

**Table:** 

Nomenclature	nicotinic acetylcholine receptor *α*8 subunit (avian)	nicotinic acetylcholine receptor *α*9 subunit	nicotinic acetylcholine receptor *α*10 subunit
HGNC, UniProt	–	*CHRNA9,* Q9UGM1	*CHRNA10,* Q9GZZ6
Commonly used antagonists	(*α*8)_5_: *α*-bungarotoxin > atropine ≥ tubocurarine ≥ strychnine	(*α*9)_5_: *α*-bungarotoxin > methyllycaconitine > strychnine ~ tropisetron > tubocurarine; *α*9*α*10: *α*-bungarotoxin > tropisetron = strychnine > tubocurarine	*α*9*α*10: *α*-bungarotoxin > tropisetron = strychnine > tubocurarine
Selective antagonists	–	*α*-bungarotoxin (*α*9*α*10), *α*-bungarotoxin ((*α*9)_5_), *α*-conotoxin RgIA (*α*9*α*10), muscarine (*α*9*α*10), muscarine ((*α*9)_5_), nicotine (*α*9*α*10), nicotine ((*α*9)_5_), strychnine ((*α*9)_5_), strychnine (*α*9*α*10)	*α*-bungarotoxin (*α*9*α*10), *α*-conotoxin RgIA (*α*9*α*10), muscarine (*α*9*α*10), nicotine (*α*9*α*10), strychnine (*α*9*α*10)
Labelled ligands	[^3^H]epibatidine ((*α*8)_5_) (pK_d_ 9.7), [^125^I]*α*-bungarotoxin (native *α*8*) (pK_d_ 8.3), [^3^H]*α*-bungarotoxin (native *α*8*) (pK_d_ 8.3)	[^3^H]methyllycaconitine (Antagonist) (pK_d_ 8.1), [^125^I]*α*-bungarotoxin (Antagonist), [^3^H]*α*-bungarotoxin (Antagonist)	[^3^H]methyllycaconitine (Antagonist) (pK_d_ 8.1)
Functional Characteristics	–	(*α*9)_5_: P_Ca_/P_Na_ = 9; *α*9*α*10: P_Ca_/P_Na_ = 9, *P*_f_ = 22%	*α*9*α*10: P_Ca_/P_Na_ = 9, *P*_f_ = 22%

**Table:** 

Nomenclature	nicotinic acetylcholine receptor *β*1 subunit	nicotinic acetylcholine receptor *β*2 subunit	nicotinic acetylcholine receptor *β*3 subunit	nicotinic acetylcholine receptor *β*4 subunit	nicotinic acetylcholine receptor *γ* subunit	nicotinic acetylcholine receptor *δ* subunit	nicotinic acetylcholine receptor *ϵ* subunit
HGNC, UniProt	*CHRNB1,* P11230	*CHRNB2,* P17787	*CHRNB3,* Q05901	*CHRNB4,* P30926	*CHRNG,* P07510	*CHRND,* Q07001	*CHRNE,* Q04844
Antagonists	–	–	–	–	–	PhTX-11 (pIC_50_ 6.2–6.3) [1156]	–
Comments	Ligand interaction data for hetero-oligomeric receptors containing these subunit are listed under the *α* subunits						

### Comments:

Commonly used agonists of nACh receptors that display limited discrimination in functional assays between receptor subtypes include A-85380, cytisine, DMPP, epibatidine, nicotine and the natural transmitter, acetylcholine. A summary of their profile across differing receptors is provided in [396] and quantitative data across numerous assay systems are summarized in [532]. Quantitative data presented in the table for commonly used antagonists and channel blockers for human receptors studied under voltage-clamp are from [141, 177, 931, 932, 942, 1333]. Type I PAMs increase peak agonist-evoked responses but have little, or no, effect on the rate of desensitization of *α*7 nicotinic ACh receptors whereas type II PAMs also cause a large reduction in desensitization (reviewed in [1320]).

## P2X receptors


Ion channels→Ligand-gated ion channels→P2Xreceptors

### Overview:

P2X receptors (**nomenclature as agreed by the NC-IUPHAR Subcommittee on P2X Receptors** [220, 590]) have a trimeric topology [503, 538, 581, 870] with two putative TM domains per P2X subunit, gating primarily Na^+^, K^+^ and Ca^2+^, exceptionally Cl^−^. The Nomenclature Subcommittee has recommended that for P2X receptors, structural criteria should be the initial basis for nomenclature where possible. X-ray crystallography indicates that functional P2X receptors are trimeric and three agonist molecules are required to bind to a single trimeric assembly in order to activate it [392, 441, 503, 581, 778]. Native receptors may occur as either homotrimers (*e.g.* P2X1 in smooth muscle) or heterotrimers (*e.g.* P2X2:P2X3 in the nodose ganglion [1264], P2X1:P2X5 in mouse cortical astrocytes [652], and P2X2:P2X5 in mouse dorsal root ganglion, spinal cord and mid pons [221, 1066]. P2X2, P2X4 and P2X7 receptor activation can lead to influx of large cationic molecules, such as NMDG^+^, Yo-Pro, ethidium or propidium iodide [954]. The permeability of the P2X7 receptor is modulated by the amount of cholesterol in the plasma membrane [846]. The hemi-channel pannexin-1 was initially implicated in the action of P2X7 [955], but not P2X2, receptors [175], but this interpretation is probably misleading [966]. Convincing evidence now supports the view that the activated P2X7 receptor is immediately permeable to large cationic molecules, but influx proceeds at a much slower pace than that of the small cations Na^+^, K^+^, and Ca^2+^ [274].

**Table:** 

Nomenclature	P2X1	P2X2	P2X3
HGNC, UniProt	*P2RX1,* P51575	*P2RX2,* Q9UBL9	*P2RX3,* P56373
Endogenous agonists	–	ATP [520] – Rat	ATP [521]
Agonists	*αβ*-meATP, BzATP, L-*βγ*-meATP	–	*αβ*-meATP, BzATP
Antagonists	TNP-ATP (pIC_50_ ~8.9) [1251], Ip_5_I (pIC_50_ ~8.5) [597], NF023 (pIC_50_ ~6.7) [1136], NF449 (pIC_50_ ~6.3) [577]	NF770 (pIC_50_ 7–8) [894], NF778 (pIC_50_ 7–8) [894], PSB-10211 (pIC_50_ ~7) [894]	TNP-ATP (pIC_50_ ~8.9) [1251], AF-906 (pIC_50_ 8.9) [523], gefapixant (pIC_50_ 8.5) [523], sivopixant (pIC_50_ 8.4) [559], eliapixant (pIC_50_ 8.1) [240], camlipixant (pIC_50_ 7.6) [371], A317491 (pIC_50_ ~7.5) [528]
Selective allosteric modulators	MRS 2219 (Positive) [522]	–	–

**Table:** 

Nomenclature	P2X4	P2X5	P2X6	P2X7
HGNC, UniProt	*P2RX4,* Q99571	*P2RX5,* Q93086	*P2RX6,* O15547	*P2RX7,* Q99572
Endogenous agonists	ATP [521]	ATP [521] – Rat	ATP [521] – Rat	ATP [521]
Agonists	*αβ*-meATP, BzATP	–	–	–
Antagonists	BAY-1797 (pIC_50_ 7) [1315], PSB-12054 (pIC_50_ 6.7) [453], 5-BDBD (pIC_50_ 5–6) [523, 894], BX-430 (pIC_50_ 5–6) [523, 894], PSB-12062 (pIC_50_ 5–6) [523, 894], paroxetine (pIC_50_ 5–6) [523, 894]	–	–	AZ10606120 (pK_d_ 8.9) [808], A804598 (pIC_0_ ~8), brilliant blue G (pIC_50_ ~8) [539], A839977 (pIC_50_ ~7.7) [291, 293, 478], A740003 (pIC_50_ 7.4) [479], decavanadate (pA_2_ = 7.4) (p*A*_2_ 7.4) [813], A438079 (pIC_50_ ~6.9) [291], AZ11657312 (salt free) (p*A*_2_ 6.1) [44]
Selective antagonists	–	–	–	JNJ-47965567 (pK_i_ 7.9) [99]
Allosteric modulators (Positive)	nimodipine [1074]	–	–	GW791343 [808, 810] – Rat, LL-37 (*CAMP,* P49913) [1213], clemastine [890], ivermectin [891], polymyxin B [348]
Allosteric modulators (Negative)	amlodipine [1074]	–	–	AZ10606120 [808], GW791343 [808, 810], KN62 [376, 1097]
Selective allosteric modulators	ivermectin (Positive) (pEC_50_ ~6.6) [591] – Rat	–	–	chelerythrine (Negative) (pIC_50_ 5.2) [1097], AZ11645373 (Negative) [811, 1149]
Comments	–	–	–	Ginsenoside compounds acts as positive allosteric modulators of P2X7 [979], however, the effects of allosteric regulators at P2X7 receptors are species-dependent.

### Comments:

A317491 and RO3 also block the P2X2:P2X3 heteromultimer [359, 528]. NF449, A317491 and RO3 are more than 10-fold selective for P2X1 and P2X3 receptors, respectively.

Agonists listed show selectivity within recombinant P2X receptors of *ca.* one order of magnitude. A804598, A839977, A740003 and A438079 are at least 10-fold selective for P2X7 receptors and show similar affinity across human and rodent receptors [291, 293, 478].

Several P2X receptors (particularly P2X1 and P2X3) may be inhibited by desensitisation using stable agonists (*e.g. αβ*-meATP); suramin and PPADS are non-selective antagonists at rat and human P2X1-3,5 and hP2X4, but not rP2X4,6,7 [140], and can also inhibit ATPase activity [223]. Ip_5_I is inactive at rP2X2, an antagonist at rP2X3 (pIC_50_ 5.6) and enhances agonist responses at rP2X4 [597]. Antagonist potency of NF023 at recombinant P2X2, P2X3 and P2X5 is two orders of magnitude lower than that at P2X1 receptors [1136]. The P2X7 receptor may be inhibited in a non-competitive manner by the protein kinase inhibitors KN62 and chelerythrine [1097], while the p38 MAP kinase inhibitor GTP*γ*S and the cyclic imide AZ11645373 show a species-dependent non-competitive action [292, 811, 812, 1149]. The pH-sensitive dye used in culture media, phenol red, is also reported to inhibit P2X1 and P2X3 containing channels [598]. Some recombinant P2X receptors expressed to high density bind [^35^S]ATP*γ*S and [^3^H]*αβ*-meATP, although the latter can also bind to 5’-nucleotidase [809]. [^3^H]A317491 and [^3^H]A804598 have been used as high affinity antagonist radioligands for P2X3 (and P2X2/3) and P2X7 receptors, respectively [293]. Several high affinity radioligands for the P2X7 receptor have been recently synthesized, some with very promising applications in the diagnosis of inflammatory diseases of the central nervous system [340, 733, 1078, 1193, 1410]. Several P2X3 antagonists have entered clinical trials for refractory chronic cough. In 2022, gefapixant was approved in Japan for the management of refractory or unexplained chronic cough [781]. Oxidized ATP covalently binds un-protonated lysine residues in the vicinity of the ATP-binding site and irreversibly inhibits the P2X7 receptor. Other plasma membrane receptors exposing available lysines may also be blocked by oATP [89, 273]. The cryoelectron microscopy structures of full-length rP2X7 receptor in apo and ATP-bound states have been resolved [796]. A proportion (<10%) of screened humans were found to possess full length P2X5 subunits (444 aa), which can assemble into a functional P2X5 receptor [596, 628]. An uncharged region at the N-terminus of P2X6 exerts an inhibitory effect on its assembly and export from the ER [914]. The P2X6 subunit also lacks nine residues in the left flipper, which is a key element in agonist docking at P2X receptors [906].

## ZAC


Ion channels→Ligand-gated ion channels→ZAC

### Overview:

The zinc-activated channel (ZAC, **nomenclature as agreed by the NC-IUPHAR Subcommittee for the Zinc Activated Channel**) is a member of the Cys-loop family that includes the nicotinic ACh, 5-HT_3_, GABA_A_ and strychnine-sensitive glycine receptors [242, 485, 1223]. The channel is likely to exist as a homopentamer of 4TM subunits that form an intrinsic cation selective channel equipermeable to Na^+^, K^+^ and Cs^+^, but impermeable to Ca^2+^ and Mg^2+^ [1223]. ZAC displays constitutive activity that can be blocked by tubocurarine, TTFB and high concentrations of Ca^2+^ [1223]. Although denoted ZAC, the channel is more potently activated by H^+^ and Cu^2+^, with greater and lesser efficacy than Zn^2+^, respectively [1223]. Orthologs of the human *ZACN* gene are present in a wide range of mammalian genomes, but notably not in the mouse or rat genomes. [242, 485].

**Table:** 

Nomenclature	ZAC
HGNC, UniProt	*ZACN,* Q401N2
Endogenous agonists	H^+^ [1223], Cu^2+^ [1223], Zn^2+^ [242, 1223]
Antagonists	tubocurarine (pIC_50_ 5.2) [242], Ca^2+^ (pIC_50_ 2) [1223]
Allosteric modulators	TTFB (Antagonist) (pIC_50_ 5.5) [757]
Functional Characteristics	Outwardly rectifying current (both constitutive and evoked by Zn^2+^)

### Comments:

A *ZACN* gene does not appear to exist in the mouse or rat genomes [242]. Although tabulated as an antagonist, it is possible that tubocurarine acts as a channel blocker. Antagonism by Ca^2+^ is voltage-independent. ZAC is not activated (at 1 mM) by transition metals including Fe^2+^, Co^2+^, Ni^2+^, Cd^2+^, or Al^3+^ [1223]. The concentration response relationship to Cu^2+^ is biphasic, with concentrations exceeding 30 *μ*M being associated with reduced activation [1223]. The N-(thiazol-2-yl)-benzamide analog TTFB has been identified as a moderately potent negative allosteric modulator of ZAC. TTFB displays negligible activity at representatives of the GABA_A_, glycine, 5-HT_3_ and nicotinic ACh receptors, and thus it constitutes a potential pharmacological tool for ZAC.

## Voltage-gated ion channels


Ion channels→Voltage-gated ion channels

### Overview:

The voltage-gated ion channels and their structural relatives comprise a superfamily encoded by at least 143 genes in the human genome and are therefore one of the largest superfamilies of signal transduction proteins, following the G protein-coupled receptors and the protein kinases in number [161]. In addition to their prominence in signal transduction, these ion channels are also among the most common drug targets. As for other large protein superfamilies, understanding the molecular relationships among family members, developing a unified, rational nomenclature for the ion channel families and subfamilies, and assigning physiological functions and pharmacological significance to each family member has been an important challenge. Some of the ion channels placed under the ‘Voltage-gated’ umbrella are not in fact gated by voltage, but for the reasons mentioned above it is useful to consider them within this superfamily. The inwardly rectifying potassium channels, two-pore domain potassium channels (K2P), ryanodine receptors (RyR) and transient receptor potential channels (TRP) are those that are NOT voltage-gated.

## CatSper and Two-Pore channels (TPC)


Ion channels→Voltage-gated ion channels→CatSper and Two-Pore channels (TPC)

### Overview:

CatSper channels (CatSper1-4, **nomenclature as agreed by NC-IUPHAR** [213]) are putative 6TM, voltage-gated, alkalinization-activated calcium permeant channels that are presumed to assemble as a tetramer of *α*-like subunits and mediate the current I_CatSper_ [599]. In mammals, CatSper subunits are structurally most closely related to individual domains of voltage-activated calcium channels (Ca_v_) [1018]. CatSper1 [1018], CatSper2 [1004] and CatSpers 3 and 4 [542, 726, 996], in common with a putative 2TM auxiliary CatSper*β* Protein [718] and two putative 1TM associated CatSper*γ* and CatSper*α* proteins [206, 1281], are restricted to the testis and localised to the principle piece of sperm tail. The novel cross-species CatSper channel inhibitor, RU1968, has been proposed as a useful tool to aid characterisation of native CatSper channels [1019].

Two-pore channels (TPCs) are structurally related to CatSpers, Ca_V_s and Na_V_s. TPCs have a 2x6TM structure with twice the number of TMs of CatSpers and half that of Ca_V_s. There are three animal TPCs (TPC1-TPC3). Humans have TPC1 and TPC2, but not TPC3. TPC1 and TPC2 are localized in endosomes and lysosomes [146]. TPC3 is also found on the plasma membrane and forms a voltage-activated, non-inactivating Na^+^ channel [149]. All the three TPCs are Na^+^-selective under whole-cell or whole-organelle patch clamp recording [150, 151, 1338]. The channels may also conduct Ca^2+^ [835].

**Table:** 

Nomenclature	CatSper1	CatSper2	CatSper3	CatSper4
HGNC, UniProt	*CATSPER1,* Q8NEC5	*CATSPER2,* Q96P56	*CATSPER3,* Q86XQ3	*CATSPER4,* Q7RTX7
Activators	CatSper1 is constitutively active, weakly facilitated by membrane depolarisation, strongly augmented by intracellular alkalinisation. In human, but not mouse, progesterone (EC_50_ ~ 8 nM) also potentiates the CatSper current (I_CatSper_). [714, 1158]	–	–	–
Channel blockers	ruthenium red (Inhibition) (pIC_50_ 5) [599] – Mouse, HC-056456 (pIC_50_ 4.7) [158], Cd^2+^ (Inhibition) (pIC_50_ 3.7) [599] – Mouse, Ni^2+^ (Inhibition) (pIC_50_ 3.5) [599] – Mouse	–	–	–
Selective channel blockers	NNC55-0396 (Inhibition) (pIC_50_ 5.7) [−80mV – 80mV] [714, 1158], mibefradil (Inhibition) (pIC_50_ 4.4–4.5) [1158]	–	–	–
Functional Characteristics	Calcium selective ion channel (Ba^2+>^Ca^2+3>^Mg^2+3>^Na^+^); quasilinear monovalent cation current in the absence of extracellular divalent cations; alkalinization shifts the voltage-dependence of activation towards negative potentials [V_1/2_ @ pH 6.0 = +87 mV (mouse); V_1/2_ @ pH 7.5 = +11mV (mouse) or pH 7.4 = +85 mV (human)]; required for I_CatSper_ and male fertility (mouse and human)	Required for I_CatSper_ and male fertility (mouse and human)	Required for I_CatSper_ and male fertility (mouse)	Required for I_CatSper_ and male fertility (mouse)

**Table:** 

Nomenclature	TPC1	TPC2
HGNC, UniProt	*TPCN1,* Q9ULQ1	*TPCN2,* Q8NHX9
Activators	phosphatidyl (3,5) inositol bisphosphate (pEC_50_ 6.5) [150]	phosphatidyl (3,5) inositol bisphosphate (pEC_50_ 6.4) [1294]
Selective activators	–	TPC2-A1-N (pEC_50_ 5.1) [381]
Channel blockers	verapamil (Inhibition) (pIC_50_ 4.6) [150], Cd^2+^ (Inhibition) (pIC_50_ 3.7) [150]	verapamil (Inhibition) (pIC_50_ 5) [1294]
Functional Characteristics	Organelle voltage-gated Na^+^-selective channel (Na^+≫^K^+≫^Ca^2+^); Required for the generation of action potential-like long depolarization in lysosomes. Voltage-dependence of activation is sensitive to luminal pH (determined from lysosomal recordings). *ψ_1/2_* @ pH4.6 = +91 mV; *ψ_1/2_* @ pH6.5 = +2.6 mV. Maximum activity requires PI(3,5)P2 and reduced [ATP], or depletion of extracellular amino acids.	Organelle voltage-independent Na^+^-selective channel (Na^+≫^K^+≫^Ca^2+^). Sensitive to the levels of PI(3,5)P2. Activated by decreases in [ATP] or depletion of extracellular amino acids

### Comments:

CatSper channel subunits expressed singly, or in combination, fail to functionally express in heterologous expression systems [1004, 1018]. The properties of CatSper1 tabulated above are derived from whole cell voltage-clamp recordings comparing currents endogenous to spermatozoa isolated from the *corpus epididymis* of wild-type and *Catsper1*^*(−/−)*^ mice [599] and also mature human sperm [714, 1158]. I_CatSper_ is also undetectable in the spermatozoa of *Catsper2*^*(−/−)*^, *Catsper3*^*(−/−)*^, *Catsper4*^*(−/−)*^, or CatSper*δ* ^(−/−)^ mice, and CatSper 1 associates with CatSper 2, 3, 4, *β, γ*, and *δ* [206, 718, 996]. Moreover, targeted disruption of *Catsper1, 2, 3, 4,* or *α* genes results in an identical phenotype in which spermatozoa fail to exhibit the hyperactive movement (whip-like flagellar beats) necessary for penetration of the egg *cumulus* and *zona pellucida* and subsequent fertilization. Such disruptions are associated with a deficit in alkalinization and depolarization-evoked Ca^2+^ entry into spermatozoa [159, 206, 996]. Thus, it is likely that the CatSper pore is formed by a heterotetramer of CatSpers1-4 [996] in association with the auxiliary subunits (*β, γ, δ*) that are also essential for function [206]. CatSper channels are required for the increase in intracellular Ca^2+^ concentration in sperm evoked by egg *zona pellucida* glycoproteins [1338]. Mouse and human sperm swim against the fluid flow and Ca^2+^ signaling through CatSper is required for the rheotaxis [815]. *In vivo,* CatSper1-null spermatozoa cannot ascend the female reproductive tracts efficiently [207, 469]. It has been shown that CatSper channels form four linear Ca^2+^ signaling domains along the flagella, which orchestrate capacitation-associated tyrosine phosphorylation [207]. The driving force for Ca^2+^ entry is principally determined by a mildly outwardly rectifying K^+^ channel (KSper) that, like CatSpers, is activated by intracellular alkalinization [862]. Mouse KSper is encoded by *mSlo3,* a protein detected only in testis [784, 862, 1396]. In human sperm, such alkalinization may result from the activation of H_v_1, a proton channel [715]. Mutations in CatSpers are associated with syndromic and non-syndromic male infertility [458]. In human ejaculated spermatozoa, progesterone (<50 nM) potentiates the CatSper current by a non-genomic mechanism and acts synergistically with intracellular alkalinisation [714, 1158]. Sperm cells from infertile patients with a deletion in CatSper2 gene lack I_CatSper_ and the progesterone response [1123]. In addition, certain prostaglandins (*e.g.* PGF_1_*α*, PGE_1_) also potentiate CatSper mediated currents [714, 1158].

In human sperm, CatSper channels are also activated by various small molecules including endocrine disrupting chemicals and proposed as a polymodal sensor [127, 127].

TPCs are the major Na^+^ conductance in lysosomes; knocking out TPC1 and TPC2 eliminates the Na^+^ conductance and renders the organelle’s membrane potential insensitive to changes in [Na^+^] (31). The channels are regulated by luminal pH [150], PI(3,5) P2 [1294], intracellular ATP and extracellular amino acids [151]. TPCs are also involved in the NAADP-activated Ca^2+^ release from lysosomal Ca^2+^ stores [146, 835]. Mice lacking TPCs are viable but have phenotypes including compromised lysosomal pH stability, reduced physical endurance [151], resistance to Ebola viral infection [1055] and fatty liver [402]. No major human disease-associated TPC mutation has been reported.

## Cyclic nucleotide-regulated channels (CNG)


Ion channels→Voltage-gated ion channels→Cyclic nucleotide-regulated channels (CNG)

### Overview:

**Cyclic nucleotide-gated (CNG) channels** are responsible for signalling in the primary sensory cells of the vertebrate visual and olfactory systems. CNG channels are voltage-independent cation channels formed as tetramers. Each subunit has 6TM, with the pore-forming domain between TM5 and TM6. CNG channels were first found in rod photoreceptors [350, 580], where light signals through rhodopsin and transducin to stimulate phosphodiesterase and reduce intracellular cyclic GMP level. This results in a closure of CNG channels and a reduced ‘dark current’. Similar channels were found in the cilia of olfactory neurons [858] and the pineal gland [297]. The cyclic nucleotides bind to a domain in the C terminus of the subunit protein: other channels directly binding cyclic nucleotides include hyperolarisation-activated, cyclic nucleotide-gated channels (HCN), ether-a-go-go and certain plant potassium channels.

The **HCN channels** are cation channels that are activated by hyperpolarisation at voltages negative to ~-50 mV. The cyclic nucleotides cyclic AMP and cyclic GMP directly bind to the cyclic nucleotide-binding domain of HCN channels and shift their activation curves to more positive voltages, thereby enhancing channel activity. HCN channels underlie pacemaker currents found in many excitable cells including cardiac cells and neurons [278, 930]. In native cells, these currents have a variety of names, such as *I*_h_, *I*_q_ and *I*_f_. The four known HCN channels have six transmembrane domains and form tetramers. It is believed that the channels can form heteromers with each other, as has been shown for HCN1 and HCN4 [27]. High resolution structural studies of CNG and HCN channels has provided insight into the the gating processes of these channels [665, 666, 693]. **A standardised nomenclature for CNG and HCN channels has been proposed by the NC-IUPHAR Subcommittee on voltage-gated ion channels** [472].

**Table:** 

Nomenclature	CNGA1	CNGA2	CNGA3	CNGB1	CNGB3
HGNC, UniProt	*CNGA1,* P29973	*CNGA2,* Q16280	*CNGA3,* Q16281	*CNGB1,* Q14028	*CNGB3,* Q9NQW8
Activators	cyclic GMP (EC_50_ ~ 30 *μ*M) ≫ cyclic AMP	cyclic GMP > cyclic AMP (EC_50_ ~ 1 *μ*M)	cyclic GMP (EC_50_ ~ 30 *μ*M) ≫ cyclic AMP	–	–
Channel blockers	dequalinium (Antagonist) (pIC_50_ 6.7) [0mV] [1034], L-(cis)-diltiazem (Antagonist) (pK_i_ 4) [−80mV – 80mV] [187]	dequalinium (Antagonist) (pIC_50_ 5.6) [0mV] [1033]	L-(cis)-diltiazem (high affinity binding requires presence of CNGB subunits)	–	L-(cis)-diltiazem (Antagonist) (pIC_50_ 5.5) [0mV] [382] – Mouse
Functional Characteristics	*γ* = 25-30 pS *P*_Ca_/*P*_Na_ = 3.1	*γ* = 35 pS *P*_Ca_/*P*_Na_ = 6.8	*γ* = 40 pS *P*_Ca_/*P*_Na_ = 10.9	–	–
Comments	–	–	–	L-(cis)-diltiazem acts as a channel blocker when CNGB1 is co-expressed with CNGA1.	–

**Table:** 

Nomenclature	HCN1	HCN2	HCN3	HCN4
HGNC, UniProt	*HCN1,* 060741	*HCN2,* Q9UL51	*HCN3,* Q9P1Z3	*HCN4,* Q9Y3Q4
Activators	cyclic AMP > cyclic GMP (both weak)	cyclic AMP > cyclic GMP	–	cyclic AMP > cyclic GMP
Channel blockers	MEL57A (pEC_50_ 6.5) [−80mV] [260] – Mouse, ivabradine (Antagonist) (pIC_50_ 5.7) [1145], ZD7288 (Antagonist) (pIC_50_ 4.7) [1144], EC18 (pEC_50_ 4.7) [260] – Mouse, Cs^+^ (Antagonist) (pIC_50_ 3.7) [−40mV] [1144]	ivabradine (Antagonist) (pIC_50_ 5.6) [1145] – Mouse, clonidine (Antagonist) (pIC_50_ 5.1) [−40mV] [613] – Mouse, MEL57A (pEC_50_ 4.9) [260] – Mouse, EC18 (pEC_50_ 4.7) [260] – Mouse, ZD7288 (Antagonist) (pIC_50_ 4.4) [1144], Cs^+^ (Antagonist) (pIC_50_ 3.7) [−40mV] [1144]	ivabradine (Antagonist) (pIC_50_ 5.7) [1145], ZD7288 (Antagonist) (pIC_50_ 4.5) [1144], Cs^+^ (Antagonist) (pIC_50_ 3.8) [−40mV] [1144]	ivabradine (Antagonist) (pIC_50_ 5.7) [1145], EC18 (pEC_50_ 5.4) [−80mV] [260], clonidine (Antagonist) (pIC_50_ 5) [−40mV] [613] – Mouse, ZD7288 (Antagonist) (pIC_50_ 4.7) [1144], MEL57A (pEC_50_ 4.1) [260], Cs^+^ (Antagonist) (pIC_50_ 3.8) [−40mV] [1144]

### Comments:

CNGA1, CNGA2 and CNGA3 express functional channels as homomers. Three additional subunits *CNGA4* (Q8IV77), *CNGB1* (Q14028) and *CNGB3* (Q9NQW8) do not, and are referred to as auxiliary subunits. The subunit composition of the native channels is believed to be as follows. Rod: CNGA1_3_/CNGB1a [75]; Cone: CNGA3_3_/CNGB3_1_ [1411] ; Olfactory neurons: CNGA2_2_/CNGA4/CNGB1b [956, 1313, 1408, 1409, 1415]. HCN channels are permeable to both Na^+^ and K^+^ ions, with a Na^+^/K^+^ permeability ratio of about 0.2. Functionally, they differ from each other in terms of time constant of activation with HCN1 the fastest, HCN4 the slowest and HCN2 and HCN3 intermediate. The compounds ZD7288 [121] and ivabradine [139] have proven useful in identifying and studying functional HCN channels in native cells. Zatebradine and cilobradine are also useful blocking agents.

## Potassium channels


Ion channels→Voltage-gated ion channels→Potassium channels

### Overview:

Activation of potassium channels regulates excitability and can control the shape of the action potential waveform. They are present in all cells within the body and can influence processes as diverse as cognition, muscle contraction and hormone secretion. Potassium channels are subdivided into families, based on their structural and functional properties. The largest family consists of potassium channels that activated by membrane depolarization, with other families consisting of channels that are either activated by a rise of intracellular calcium ions or are constitutively active. A standardised nomenclature for potassium channels has been proposed by the **NC-IUPHAR Subcommittees** on potassium channels [391, 419, 644, 1311], which has placed cloned channels into groups based on gene family and structure of channels that exhibit 6, 4 or 2 transmembrane domains (TM).

## Calcium- and sodium-activated potassium channels (K_Ca_, K_Na_)


$$\text{Ion channels}\to \text{Voltage-gated ion channels}\to \text{Potassium channels}\to \text{Calcium-}\;\text{and sodium-activated potassium channels}\;(K_{\text{Ca}},K_{\text{Na}})$$

### Overview:

Calcium- and sodium- activated potassium channels are members of the 6TM family of K channels which comprises the voltage-gated K_V_ subfamilies, including the KCNQ subfamily, the EAG subfamily (which includes hERG channels), the Ca^2+^-activated Slo subfamily (actually with 6 or 7TM) and the Ca^2+^- and Na^+^-activated SK subfamily (**nomenclature as agreed by the NC-IUPHAR Subcommittee on Calcium- and sodium-activated potassium channels** [556]). As for the 2TM family, the pore-forming a subunits form tetramers and heteromeric channels may be formed within subfamilies (*e.g.* K_V_1.1 with K_V_1.2; KCNQ2 with KCNQ3).

**Table:** 

Nomenclature	K_Ca_1.1
HGNC, UniProt	*KCNMA1,* Q12791
Activators	NS004, NS1619
Inhibitors	paxilline (pK_i_ 8.7) [0mV] [1061] – Mouse
Channel blockers	charybdotoxin, iberiotoxin, tetraethylammonium
Functional Characteristics	Maxi K_Ca_

**Table:** 

Nomenclature	K_Ca_2.1	K_Ca_2.2	K_Ca_2.3
HGNC, UniProt	*KCNN1,* Q92952	*KCNN2,* Q9H2S1	*KCNN3,* Q9UGI6
Activators	EBIO (Agonist) Concentration range: 2×10^−3^M [−80mV] [948, 1308], NS309 (Agonist) Concentration range: 3×10^−8^M-1×10^−7^M [−90mV] [1155, 1308]	NS309 (Agonist) (pEC_50_ 6.2) Concentration range: 3×10^−8^M-1×10^−7^M [947, 1155, 1308], EBIO (Agonist) (pEC_50_ 3.3) [947, 1308], EBIO (Agonist) (pEC_50_ 3) Concentration range: 2×10^−3^M [154, 948] – Rat	EBIO (Agonist) (pEC_50_ 3.8) [1308, 1326], NS309 (Agonist) Concentration range: 3×10^−8^M [1155, 1308]
Inhibitors	UCL1684 (pIC_50_ 9.1) [1154, 1308], apamin (pIC_50_ 7.9–8.5) [1092, 1147, 1154]	UCL1684 (pIC_50_ 9.6) [339, 1308], apamin (pK_d_ 9.4) [524]	apamin (pIC_50_ 7.9–9.1) [1194, 1326], UCL1684 (pIC_50_ 8–9) [339, 1308]
Channel blockers	tetraethylammonium (pIC_50_ 2.7) [1308]	tetraethylammonium (pIC_50_ 2.7) [1308]	tetraethylammonium (pIC_50_ 2.7) [1308]
Functional Characteristics	SK_Ca_	SK_Ca_	SK_Ca_
Comments	The rat isoform does not form functional channels when expressed alone in cell lines. N- or C-terminal chimeric constructs permit functional channels that are insensitive to apamin [1308]. Heteromeric channels are formed between K_Ca_2.1 and 2.2 subunits that show intermediate sensitivity to apamin [211].	–	–

**Table:** 

Nomenclature	K_Ca_3.1	K_Na_1.1	K_Na_3.1	K_Ca_5.1
HGNC, UniProt	*KCNN4,* O15554	*KCNT1,* Q5JUK3	*KCNT2,* Q6UVM3	*KCNU1,* A8MYU2
Activators	NS309 (Agonist) (pEC_50_ 8) [−90mV] [1155, 1308], SKA-121 (Agonist) (pEC_50_ 7) [217], SKA-111 (Agonist) (pEC_50_ 6.9) [217], EBIO (Agonist) (pEC_50_ 4.1–4.5) [−100mV – −50mV] [948, 1177, 1308]	bithionol (Agonist) (pEC_50_ 5–6) [1358] – Rat, niclosamide (Agonist) (pEC_50_ 5.5) [106], loxapine (Agonist) (pEC_50_ 5.4) [106]	niflumic acid (Agonist) (pEC_50_ 8.7) [233, 375]	–
Gating inhibitors	–	bepridil (pIC_50_ 5–6) [1358] – Rat	–	–
Channel blockers	charybdotoxin (pIC_50_ 7.6–8.7) [533, 549]	quinidine (pIC_50_ 4) [98, 1358] – Rat	Ba^2+^ (Inhibition) (pIC_50_ 3) [98], quinidine (Inhibition) Concentration range: 1×10^−3^M [98] – Rat	quinidine Concentration range: 2×10^−5^M [1189, 1331] – Mouse
Selective channel blockers	TRAM-34 (Inhibition) (pK_d_ 7.6–8) [620, 1336], senicapoc (Inhibition) (pIC_50_ 8) [1146]	–	–	–
Functional Characteristics	IK_Ca_	K_Na_	K_Na_	Sperm pH-regulated K^+^ current, KSPER

## Inwardly rectifying potassium channels (K_IR_)


$$\text{Ion channels}\to \text{Voltage-gated ion channels}\to \text{Potassium channels}\to \text{Inwardly rectifying potassium channels}\;(K_{\text{IR}})$$

### Overview:

The 2TM domain family of K channels are also known as the inward-rectifier K channel family. This family includes the strong inward-rectifier K channels (K_ir_2.x) that are constitutively active, the G-protein-activated inward-rectifier K channels (K_ir_3.x) and the ATP-sensitive K channels (K_ir_6.x, which combine with sulphonylurea receptors (SUR1-3)). The pore-forming *α* subunits form tetramers, and heteromeric channels may be formed within subfamilies (*e.g.* K_ir_3.2 with K_ir_3.3).

**Table:** 

Nomenclature	K_ir_1.1
HGNC, UniProt	*KCNJ1,* P48048
Ion Selectivity and Conductance	NH_4_^+^ [62pS] > K^+^ [38. pS] > Tl^+^ [21pS] > Rb^+^ [15pS] (Rat) [200, 468]
Channel blockers	tertiapin-Q (Inhibition) (pIC_50_ 8.9) [544], Ba^2+^ (Antagonist) (pIC_50_ 2.3–4.2) Concentration range: 1×10^−4^M [*voltage dependent* 0mV – −100mV] [468, 1416] – Rat, Cs^+^ (Antagonist) (pIC_50_ 2.9) [*voltage dependent* −120mV] [1416] – Rat
Functional Characteristics	K_ir_1.1 is weakly inwardly rectifying, as compared to classical (strong) inward rectifiers.

**Table:** 

Nomenclature	K_ir_2.1	K_ir_2.2	K_ir_2.3	K_ir_2.4
HGNC, UniProt	*KCNJ2,* P63252	*KCNJ12,* Q14500	*KCNJ4,* P48050	*KCNJ14,* Q9UNX9
Endogenous activators	PIP_2_ (Agonist) Concentration range: 1× 10^−5^M-5×10^−5^M [−30mV] [491, 1015, 1135] – Mouse	–	–	–
Endogenous inhibitors	–	Intracellular Mg^2+^ (pIC_50_ 5) [40mV] [1355]	–	Intracellular Mg^2+^
Gating inhibitors	–	Ba^2+^ (Antagonist) Concentration range: 5×10^−5^M [−150mV – −50mV] [1179] – Mouse, Cs^+^ (Antagonist) Concentration range: 5×10^−6^M-5×10^−5^M [−150mV – −50mV] [1179] – Mouse	–	–
Endogenous channel blockers	spermine (Antagonist) (pK_d_ 9.1) [*voltage dependent* 40mV] [514, 1360] – Mouse, spermidine (Antagonist) (pK_d_ 8.1) [*voltage dependent* 40mV] [1360] – Mouse, putrescine (Antagonist) (pK_d_ 5.1) [*voltage dependent* 40mV] [514, 1360] – Mouse, Intracellular Mg^2+^ (Antagonist) (pK_d_ 4.8) [*voltage dependent* 40mV] [1360] – Mouse	–	Intracellular Mg^2+^ (Antagonist) (pK_d_ 5) [*voltage dependent* 50mV] [730], putres-cine (Antagonist) Concentration range: 5× 10’^5^M-1×10-^3^M [−80mV- 80mV] [730], spermidine (Antagonist) Concentration range: 2.5×1 0’^5^M-1×10-^3^M [−80mV - 80mV] [730], spermine (Antagonist) Concentration range: 5×1 0^−5^M-1×10^−3^M [−80mV- 80mV] [730]	–
Channel blockers	Ba^2+^ (Antagonist) (pK_d_ 3.9–5.6) Concentration range: 1×10^−6^M-1×10^−4^M [*voltage dependent* 0mV – −80mV] [18] – Mouse, Cs^+^ (Antagonist) (pK_d_ 1.3–4) Concentration range: 3×10^−5^M-3×10^−4^M [*voltage dependent* 0mV – −102mV] [6] – Mouse	–	Ba^2+^ (Antagonist) (pIC_50_ 5) Concentration range: 3×10^−6^M-5×10^−4^M [−60mV] [769, 988, 1190], Cs^+^ (Antagonist) (pK_i_ 1.3–4.5) Concentration range: 3×10^−6^M-3×10^−4^M [0mV – −130mV] [769]	Cs^+^ (Antagonist) (pK_d_ 3–4.1) [*voltage dependent* −60mV – −100mV] [497], Ba^2+^ (Antagonist) (pK_d_ 3.3) [*voltage dependent* 0mV] [497]
Functional Characteristics	IK_1_ in heart, ‘strong’ inward-rectifier current	IK_1_in heart, ‘strong’ inward-rectifier current	IK_1_ in heart, ‘strong’ inward-rectifier current	IK_1_ in heart, ‘strong’ inward-rectifier current
Comments	K_ir_2.1 is also inhibited by intracellular polyamines	K_ir_2.2 is also inhibited by intracellular polyamines	K_ir_2.3 is also inhibited by intracellular polyamines	K_ir_2.4 is also inhibited by intracellular polyamines

**Table:** 

Nomenclature	K_ir_3.1	K_ir_3.2	K_ir_3.3	K_ir_3.4
HGNC, UniProt	*KCNJ3,* P48549	*KCNJ6,* P48051	*KCNJ9,* Q92806	*KCNJ5,* P48544
Endogenous activators	PIP_2_ (Agonist) (pK_d_ 6.3) Concentration range: 5×10^−5^M [physiological voltage] [491]	PIP_2_ (Agonist) (pK_d_ 6.3) Concentration range: 5×10^−5^M [physiological voltage] [491]	PIP_2_ [460]	PIP_2_ [77, 460]
Gating inhibitors	–	pimozide (Antagonist) (pEC_50_ 5.5) [−70mV] [617] – Mouse	–	–
Channel blockers	tertiapin-Q (Antagonist) (pIC_50_ 7.9) [543], Ba^2+^ (Antagonist) (PIC_50_ 4.7) [239] – Rat	desipramine (Antagonist) (pIC_50_ 4.4) [−70mV] [618] – Mouse	–	tertiapin-Q (Antagonist) (pIC_50_ 7.9) [543]
Functional Characteristics	G protein-activated inward-rectifier current	G protein-activated inward-rectifier current	G protein-activated inward-rectifier current	G protein-activated inward-rectifier current
Comments	K_ir_3.1 is also activated by G*βγ*. K_ir_3.1 is not functional alone. The functional expression of K_ir_3.1 in *Xenopus oocytes* requires coassembly with the endogenous *Xenopus* K_ir_3.5 subunit. The major functional assembly in the heart is the K_ir_3.1/3.4 heteromultimer, while in the brain it is K_ir_3.1/3.2, K_ir_3.1/3.3 and K_ir_3.2/3.3.	K_ir_3.2 is also activated by G*βγ*. K_ir_3.2 forms functional heteromers with K_ir_3.1/3.3.	K_ir_3.3 is also activated by G*βγ*	K_ir_3.4 is also activated by G*βγ*

**Table:** 

Nomenclature	K_ir_4.1	K_ir_4.2	K_ir_5.1
HGNC, UniProt	*KCNJ10,* P78508	*KCNJ15,* Q99712	*KCNJ16,* Q9NPI9
Channel blockers	Ba^2+^ (Antagonist) Concentration range: 3×10^−6^M-1×10^−3^M [−160mV – 60mV] [625, 1182, 1187] – Rat, Cs^+^ (Antagonist) Concentration range: 3×10^−5^M-3×10^−4^M [−160mV – 50mV] [1182] – Rat	Ba^2+^ (Antagonist) Concentration range: 1×10^−5^M-1×10^−4^M [−120mV – 100mV] [946] – Mouse, Cs^+^ (Antagonist) Concentration range: 1×10^−5^M-1×10^−4^M [−120mV – 100mV] [946] – Mouse	Ba^2+^ (Antagonist) Concentration range: 3×10^−3^M [−120mV – 20mV] [1186] – Rat
Functional Characteristics	Inward-rectifier current	Inward-rectifier current	Weakly inwardly rectifying

**Table:** 

Nomenclature	K_ir_6.1	K_ir_6.2	K_ir_7.1
HGNC, UniProt	*KCNJ8,* Q15842	*KCNJ11,* Q14654	*KCNJ13,* O60928
Associated subunits	SUR1, SUR2A, SUR2B	SUR1, SUR2A, SUR2B	–
Activators	cromakalim, diazoxide (Agonist) Concentration range: 2×10^−4^M [−60mV] [1353] – Mouse, minoxidil, nicorandil (Agonist) Concentration range: 3×10^−4^M [−60mV – 60mV] [1353] – Mouse	diazoxide (Agonist) (pEC_50_ 4.2) [physiological voltage] [504] – Mouse, cromakalim (Agonist) Concentration range: 3×10^−5^M [−60mV] [505] – Mouse, minoxidil, nicorandil	–
Inhibitors	glibenclamide, tolbutamide	glibenclamide, tolbutamide	–
Channel blockers	–	–	Ba^2+^ (Antagonist) (pK_i_ 3.2) [*voltage dependent* −100mV] [294, 636, 650, 937], Cs^+^ (Antagonist) (pK_i_ 1.6) [*voltage dependent* −100mV] [294, 636, 937]
Functional Characteristics	ATP-sensitive, inward-rectifier current	ATP-sensitive, inward-rectifier current	Inward-rectifier current

## Two-pore domain potassium channels (K_2P_)


$$\text{Ion channels}\to \text{Voltage-gated ion channels}\to \text{Potassium channels}\to \text{Two-pore domain potassium channels}\;(K_{2P})$$

### Overview:

The 4TM family of K channels mediate many of the background potassium currents observed in native cells. They are open across the physiological voltage-range and are regulated by a wide array of neurotransmitters and biochemical mediators. The pore-forming *α*-subunit contains two pore loop (P) domains and two subunits assemble to form one ion conduction pathway lined by four P domains. It is important to note that single channels do not have two pores but that each subunit has two P domains in its primary sequence; hence the name two-pore domain, or K_2P_ channels (and not two-pore channels). Some of the K_2P_ subunits can form heterodimers across subfamilies (*e.g.* K_2P_3.1 with K_2P_9.1). The nomenclature of 4TM K channels in the literature is still a mixture of IUPHAR and common names. The suggested division into subfamilies, described in the More detailed introduction, is based on similarities in both structural and functional properties within subfamilies and this explains the "common abbreviation" nomenclature in the tables below.

**Table:** 

Nomenclature	K_2P_1.1	K_2P_2.1	K_2P_3.1	K_2P_4.1
Common abbreviation	TWIK1	TREK1	TASK1	TRAAK1
HGNC, UniProt	*KCNK1*, O00180	*KCNK2*, O95069	*KCNK3*, O14649	*KCNK4*, Q9NYG8
Endogenous activators	–	arachidonic acid (studied at 1-10 *μ*M) (pEC_50_ 5) [941]	–	arachidonic acid (studied at 1-10 *μ*M) [351]
Activators	–	GI-530159 (pEC_50_ 6.1) [735], BL-1249 (pEC_50_ 5.3) [986], chloroform (studied at 1-5 mM) Concentration range: 8×10^−3^M [940], halothane (studied at 1-5 mM) [940], isoflurane (studied at 1-5 mM) [940]	halothane (studied at 1-10 mM) [662]	riluzole (studied at 1-100 *μ*M) [306]
Inhibitors	–	norfluoxetine (pIC_50_ 5.1) [586]	–	–
Channel blockers	–	–	R-(+)-methanandamide (pIC_50_ ~6.2) [765], anandamide (pIC_50_ ~6.2) [765]	–
Functional Characteristics	Background current	Background current	Background current	Background current
Comments	K_2P_1.1 is inhibited by acid pH_o_ external acidification with a pK_a_ ~6.7 [981]. K_2P_1 forms heterodimers with K_2P_3 and K_2P_9 [982].	K_2P_2.1 is also activated by membrane stretch, heat and acid pH_i_ [764, 766]. K_2P_2 can heterodimerize with K_2P_4 [109] and K_2P_10 [687].	Knock-out of the *kcnk3* gene leads to a prolonged QT interval in mice [250] and disrupted development of the adrenal cortex [445]. K_2P_3.1 is inhibited by acid pH_o_ with a pK_a_ of 6.4 [731 ]. K_2P_3 forms heterodimers with K_2P_1 [982] and K_2P_9 [231].	K_2P_4 is activated by membrane stretch [763], and increased temperature (~12 to 20-fold between 17 and 40°C [564]) and can heterodimerize with K_2P_2 [109].

**Table:** 

Nomenclature	K_2P_5.1	K_2P_6.1	K_2P_7.1	K_2P_9.1	K_2P_10.1
Common abbreviation	TASK2	TWIK2	–	TASK3	TREK2
HGNC, UniProt	*KCNK5*, O95279	*KCNK6*, Q9Y257	*KCNK7*, Q9Y2U2	*KCNK9*, Q9NPC2	*KCNK10*, P57789
Endogenous activators	–	–	–	–	arachidonic acid (studied at 1-10 *μ*M) [683]
Activators	–	–	–	halothane (studied at 1-5 mM) [1184]	GI-530159 [735], halothane (studied at 1-5 mM) [683]
Inhibitors	–	–	–	R-(+)-methanandamide (studied at 1-10 *μ*M) [1006], anandamide (studied at 1-10 *μ*M) [1006]	–
Functional Characteristics	Background current	Unknown	Unknown	Background current	Background current
Comments	K_2P_5.1 is activated by alkaline pH_o_ [1021 ]. Knockout of the kcnk5 gene in mice is associated with metabolic acidosis, hyponatremia and hypotension due to impaired bicarbonate handling in the kidney [1303], as well as deafness [165]. The T108P mutation is associated with Balkan Endemic Nephropathy in humans [1217].	–	–	K_2P_9.1 is also inhibited by acid pH_o_ with a pK_a_ of ~6 [1006]. Imprinting of the *KCNK9* gene is associated with Birk Barel syndrome [66]. K_2P_9 can form heterodimers with K_2P_1 [982] or K_2P_3 [231].	K_2P_10.1 is also activated by membrane stretch [683] and can heterodimerize with K_2P_2 [687].

**Table:** 

Nomenclature	K_2P_12.1	K_2P_13.1	K_2P_15.1	K_2P_16.1	K_2P_17.1	K_2P_18.1
Common abbreviation	THIK2	THIK1	TASK5	TALK1	TALK2	TRESK
HGNC, UniProt	*KCNK12*, Q9HB15	*KCNK13*, Q9HB14	*KCNK15*, Q9H427	*KCNK16*, Q96T55	*KCNK17*, Q96T54	*KCNK18*, Q7Z418
Endogenous inhibitors	–	–	–	–	–	arachidonic acid (studied at 10-50 *μ*M) [1063]
Inhibitors	–	halothane (studied at ~5 mM) [110]	–	–	–	–
Functional Characteristics	Does not function as a homodimer [1005] but can form afunctional heterodimer with K_2P_13 [110].	Background current	Unknown	Background current	Background current	Background current
Comments	–	Forms a heterodimer with K_2P_12 [110].	–	K_2P_16.1 current is increased by alkaline pH_o_ with a pK_a_ of 7.8 [565].	K_2P_17.1 current is increased by alkaline pH_o_ with a pK_a_ of 8.8 [565].	A frame-shift mutation (F139WfsX24) in the *KCNK18* gene, is associated with migraine with aura in humans [651].

### Comments:

The K_2P_6, K_2P_7.1, K_2P_15.1 and K_2P_12.1 subtypes, when expressed in isolation, are nonfunctional. All 4TM channels are insensitive to the classical potassium channel blockers tetraethylammonium and fampridine, but are blocked to varying degrees by Ba^2+^ ions.

## Voltage-gated potassium channels (K_v_)


$$\text{Ion channels}\to \text{Voltage-gated ion channels}\to \text{Potassium channels}\to \text{Voltage-gated potassium channels}\;(K_{V})$$

### Overview:

The 6TM family of K channels comprises the voltage-gated K_V_subfamilies, the EAG subfamily (which includes hERG channels), the Ca^2+^-activated Slo subfamily (actually with 7TM, termed BK) and the Ca^2+^-activated SK subfamily. These channels possess a pore-forming *α* subunit that comprise tetramers of identical subunits (homomeric) or of different subunits (heteromeric). Heteromeric channels can only be formed within subfamilies (e.g. K_v_1.1 with K_v_1.2; K_v_7.2 with K_v_7.3). The pharmacology largely reflects the subunit composition of the functional channel.

**K_v_7 channels** K_v_7.1-K_v_7.5 (KCNQ1-5) K^+^ channels are voltage-gated K^+^ channels with major roles in neurons, muscle cells and epithelia where they underlie physiologically important K^+^ currents, such as the neuronal M-current and the cardiac IKs. Genetic deficiencies in all five KCNQ genes result in human excitability disorders, including epilepsy, autism spectrum disorders, cardiac arrhythmias and deafness. Thanks to the recent knowledge of the structure and function of human KCNQ-encoded proteins, these channels are increasingly used as drug targets for treating diseases [1, 551, 1248].

**Table:** 

Nomenclature	K_v_1.1	K_v_1.2	K_v_1.3	K_v_1.4	K_v_1.5	K_v_1.6	K_v_1.7
HGNC, UniProt	*KCNA1*, Q09470	*KCNA2*, P16389	*KCNA3*, P22001	*KCNA4*, P22459	*KCNA5*, P22460	*KCNA6*, P17658	*KCNA7*, Q96RP8
Associated subunits	K_v_1.2, K_v_1.4, K_v*β*_1 and K_v*β*_2 [218]	K_v_1.1, K_v_1.4, K_v_ *β*1 and K_v_ *β*2 [218]	K_v_1.1, K_v_1.2, K_v_1.4, K_v_1.6 , K_v_ *β*1 and K_v_ *β2* [218]	K_v_1.1, K_v_1.2, K_v*β*_1 and K_v*β*_2 [218]	K_v_ *β*1 and K_v_ *β*2	K_v_ *β*1 and K_v_ *β*2	K_v_ *β*1 and K_v_ *β*2
Channel blockers	*α*-dendrotoxin (pEC_50_ 7.7–9) [407, 499] – Rat, margatoxin (Inhibition) (pIC_50_ 8.4) [76], tetraethylammonium (Inhibition) (pK_d_ 3.5) [407] – Mouse	margatoxin (Inhibition) (pIC_50_ 11.2) [76], *α*-dendrotoxin (pIC_50_ 78–9.4) [407, 499] – Rat, noxiustoxin (pK_d_ 8.7) [407] – Rat, *κ* M-conotoxin RIIIK (pIC_50_ 6.6) [OmV] [347]	margatoxin (pIC_50_ 10–10.3) [374, 384], noxiustoxin (pK_d_ 9) [407] – Mouse, maurotoxin (pIC_50_ 6.8) [1027], tetraethylammonium (pK_d_ 2) [407] – Mouse	fampridine (pIC_50_ 1.9) [1159] – Rat	fampridine (pIC_50_ 4.3) [343]	α-dendrotoxin (pIC_50_ 7.7) [411], tetraethylammonium (pIC_50_ 2.2) [411]	noxiustoxin (pIC_50_ 7.7) [562] – Mouse, fampridine (pIC_50_ 3.6) [562] – Mouse
Selective channel blockers	–	–	correolide (pIC_50_ 7.1) [344]	–	–	–	–
Functional Characteristics	K_V_	K_V_	K_v_	K_A_	K_v_	K_V_	K_V_
Comments	–	–	Resistant to dendrotoxins	Resistant to dendrotoxins	Resistant to external TEA	–	–

**Table:** 

Nomenclature	K_v_1.8	K_v_2.1	K_v_2.2	K_v_3.1	K_v_3.2	K_v_3.3	K_v_3.4
HGNC, UniProt	*KCNA10*, Q16322	*KCNB1*, Q14721	*KCNB2*, Q92953	*KCNC1*, P48547	*KCNC2*, Q96PR1	*KCNC3*, Q14003	*KCNC4*, Q03721
Associated subunits	K_v_ *β*1 and K_v_ *β*2	K_v_5.1, K_v_6.1-6.4, K_v_8.1-8.2 and K_v_9.1-9.3	K_v_5.1, K_v_6.1-6.4, K_v_8.1-8.2 and K_v_9.1-9.3	–	–	–	MiRP2 is an associated subunit for K_v_3.4
Gating inhibitors	–	RY796 (pIC_50_ 6.6) [454], RY785 (pIC_50_ 5.9) [454], GxTx-1E (pIC_50_ 2) [455]	GxTx-1E (pK_d_ 8.6) [455], RY796 (pIC_50_ 7.1) [454], RY785 (pIC_50_ 6.7) [454]	–	–	–	–
Channel blockers	fampridine (pIC_50_ 2.8) [657]	tetraethylammonium (Pore blocker) (pIC_50_ 2) [444] – Rat	fampridine (pIC_50_ 2.8) [1076], tetraethylammonium (pIC_50_ 2.6) [1076]	fampridine (pIC_50_ 4.5) [407] – Mouse, tetraethylammonium (pIC_50_ 3.7) [407] – Mouse	fampridine (pIC_50_ 4.6) [702] – Rat, tetraethylammonium (pIC_50_ 4.2) [702] – Rat	tetraethylammonium (pIC_50_ 3.9) [1240] – Rat	tetraethylammonium (pIC_50_ 3.5) [1020, 1084] – Rat
Selective channel blockers	–	–	–	–	–	–	sea anemone toxin BDS-I (pIC_50_ 7.3) [285] – Rat
Functional Characteristics	K_V_	K_V_	–	K_V_	K_V_	K_A_	K_A_

**Table:** 

Nomenclature	K_v_4.1	K_v_4.2	K_v_4.3
HGNC, UniProt	*KCND1*, Q9NSA2	*KCND2*, Q9NZV8	*KCND3*, Q9UK17
Associated subunits	KChIP 1-4, DP66, DPP10	KChIP 1-4, DPP6, DPP10, K_v*β*_1, NCS-1, Na_v*β*_1	KChIP 1-4, DPP6 and DPP10, MinK, MiRPs
Channel blockers	fampridine (pIC_50_ 2) [509]	–	–
Functional Characteristics	K_A_	K_A_	K_A_

**Table:** 

Nomenclature	K_v_5.1	K_v_6.1	K_v_6.2	K_v_6.3	K_v_6.4
HGNC, UniProt	*KCNF1*, Q9H3M0	*KCNG1*, Q9UIX4	*KCNG2*, Q9UJ96	*KCNG3*, Q8TAE7	*KCNG4*, Q8TDN1

**Table:** 

Nomenclature	K_v_7.1	K_v_7.2	K_v_7.3	K_v_7.4	K_v_7.5
HGNC, UniProt	*KCNQ1*, P51787	*KCNQ2*, O43526	*KCNQ3*, O43525	*KCNQ4*, P56696	*KCNQ5*, Q9NR82
Activators	ML277 (pEC_50_ 6.6) [793]	–	gabapentin (pEC_50_ 8.3) [779], retigabine (pEC_50_ 6.2) [1192]	retigabine (pEC_50_ 5.2) [1192]	retigabine (pEC_50_ 5) [307]
Selective activators	–	–	–	retigabine derivative 10g (pEC_50_ 6) [1288]	gabapentin (pEC_50_ 8.7) [779], retigabine derivative 10g (pEC_50_ 6) [1288]
Inhibitors	XE991 (pK_d_ 6.1) [1284], linopirdine (pIC_50_ 4.4) [902] – Mouse	XE991 (pIC_50_ 6.2) [1285], linopirdine (pIC_50_ 5.3) [1285]	linopirdine (pIC_50_ 5.4) [1285] – Rat	XE991 (pIC_50_ 5.3) [1131], linopirdine (pIC_50_ 4.9) [1131 ]	linopirdine (pK_d_ 4.8) [680]
Sub/family-selective inhibitors	–	–	–	–	XE991 (pIC_50_ 4.2) [1083]
Channel blockers	JNJ303 (pIC_50_ 7.2) [1220]	tetraethylammonium (pIC_50_ 3.5–3.9) [423, 1317]	–	tetraethylammonium (pIC_50_ 1.3) – [59]	–
Allosteric modulators	–	retigabine (Activation) (pEC_50_ 5.6) [697, 1192]	–	–	–
Functional Characteristics	cardiac IK_5_	M current as a heteromer between K_V_7.2 and K_V_7.3	M current as heteromeric K_v_7.2/K_v_7.3 or K_v_7.3/K_v_7.5	–	M current as heteromeric K_v_7.3/K_v_7.5
Comments	Polyunsaturated fatty acids (PUFA) activate K_v_7.1 (KCNQ1) [660]. The PUFA analogue DHA-glycine is a selective activator of IKS with pEC_50_ 5.2 [115]. A single binding site for ML277 was identified, localized to a pocket lined by the S4-S5 linker, S5 and S6 helices of two separate subunits [749, 1319]. 3D structures have revealed the structural basis of hKCNQ1 modulation and gating [1164, 1165].	–	–	Two PIP_2_ molecules are identified in the open-state structure of K_v_7.4 (KCNQ4), which act as a bridge to couple the voltage-sensing domain (VSD) and pore domain (PD) of KCNQ4 leading to the channel opening [695, 1412]. In K_v_7.4, retigabine nestles in each fenestration, whereas linopirdine resides in a cytosolic cavity underneath the channel’s inner gate [695].	–

**Table:** 

Nomenclature	K_v_8.1	K_v_8.2	K_v_9.1	K_v_9.2	K_v_9.3	K_v_10.1	K_v_10.2
HGNC, UniProt	*KCNV1*, Q6PIU1	*KCNV2*, Q8TDN2	*KCNS1*, Q96KK3	*KCNS2*, Q9ULS6	*KCNS3*, Q9BQ31	*KCNH1*, O95259	*KCNH5*, Q8NCM2

**Table:** 

Nomenclature	K_v_11.1	K_v_11.2	K_v_11.3	K_v_12.1	K_v_12.2	K_v_12.3
HGNC, UniProt	*KCNH2*, Q12809	*KCNH6*, Q9H252	*KCNH7*, Q9NS40	*KCNH8*, Q96L42	*KCNH3*, Q9ULD8	*KCNH4*, Q9UQ05
Associated subunits	minK (KCNE1) and MiRP1 (KCNE2)	minK (KCNE1)	minK(KCNE1)	minK (KCNE1)	minK (KCNE1) and MiRP2 (KCNE3)	–
Channel blockers	astemizole (pIC_50_ 9) [1421], terfenadine (pIC_50_ 7.3) [1010], disopyramide (Inhibition) (pIC_50_ 4) [587]	–	–	–	–	–
Sub/family-selective channel blockers	E4031 (pIC_50_ 8.1) [1420]	–	–	–	–	–
Selective channel blockers	dofetilide (Inhibition) (pK_i_ 8.2) [1114], ibutilide (pIC_50_ 7.6–8) [587, 962]	–	–	–	–	–
Functional Characteristics	cardiac IK_R_	–	–	–	–	–
Comments	RPR260243 is an activator of K_v_11.1 [567].	–	–	–	–	–

## Ryanodine receptors (RyR)


Ion channels→Voltage-gated ion channels→Ryanodine receptors (RyR)

### Overview:

The ryanodine receptors (RyRs) are found on intracellular Ca^2+^ storage/release organelles. The family of RyR genes encodes three highly related Ca^2+^ release channels: RyR1, RyR2 and RyR3, which assemble as large tetrameric structures. These RyR channels are ubiquitously expressed in many types of cells and participate in a variety of important Ca^2+^ signaling phenomena (neurotransmission, secretion, *etc.*). In addition to the three mammalian isoforms described below, various nonmammalian isoforms of the ryanodine receptor have been identified [1170]. The function of the ryanodine receptor channels may also be influenced by closely associated proteins such as the tacrolimus (FK506)-binding protein, calmodulin [1354], triadin, calsequestrin, junctin and sorcin, and by protein kinases and phosphatases. Recent studies solving the structure of the ryanodine receptor have shed light on the structural basis of ryanodine receptor function [see, for example, Samso (2017) [1060] and Meissner (2017) [805]].

**Table:** 

Nomenclature	RyR1	RyR2	RyR3
HGNC, UniProt	*RYR1*, P21817	*RYR2*, Q92736	*RYR3*, Q15413
Endogenous activators	cytosolic ATP (endogenous; mM range), luminal Ca^2+^ (endogenous), cytosolic Ca^2+^ (endogenous; *μ*M range)	cytosolic ATP (endogenous; mM range), cytosolic Ca^2+^ (endogenous; *μ*M range), luminal Ca^2+^ (endogenous)	cytosolic ATP (endogenous; mM range), cytosolic Ca^2+^ (endogenous; *μ*M range)
Activators	caffeine (pharmacological; mM range), ryanodine (pharmacological; nM - *μ*M range), suramin (pharmacological; *μ*M range)	caffeine (pharmacological; mM range), ryanodine (pharmacological; nM - *μ*M range), suramin (pharmacological; *μ*M range)	caffeine (pharmacological; mM range), ryanodine (pharmacological; nM - *μ*M range)
Endogenous antagonists	cytosolic Ca^2+^ Concentration range: >1×10^−4^M, cytosolic Mg^2+^ (mM range)	cytosolic Ca^2+^ Concentration range: >1×10^−3^M, cytosolic Mg^2+^ (mM range)	cytosolic Ca^2+^ Concentration range: >1×10^−3^M, cytosolic Mg^2+^ (mM range)
Antagonists	dantrolene	–	dantrolene
Channel blockers	procaine, ruthenium red, ryanodine Concentration range: >1×10^−4^M	procaine, ruthenium red, ryanodine Concentration range: >1×10^−4^M	ruthenium red
Functional Characteristics	Ca^2+^: (*P*_Ca_/*P*_K_6) single-channel conductance: 90 pS (50 mM Ca^2+^), 770 pS (200 mM K^+^)	Ca^2+^: (*P*_Ca_/*P*_K_6) single-channel conductance: 90 pS (50 mM Ca^2+^), 720 pS (210 mM K^+^)	Ca^2+^: (*P*_Ca_/*P*_K_6) single-channel conductance: 140 pS (50 mM Ca^2+^), *777* pS (250 mM K^+^)
Comments	RyR1 is also activated by depolarisation *via* DHP receptor, calmodulin at low cytosolic Ca^2+^ concentrations, CaM kinase and PKA; antagonised by calmodulin at high cytosolic Ca^2+^ concentrations	RyR2 is also activated by CaM kinase and PKA; antagonised by calmodulin at high cytosolic Ca^2+^ concentrations	RyR3 is also activated by calmodulin at low cytosolic Ca^2+^ concentrations; antagonised by calmodulin at high cytosolic Ca^2+^ concentrations

### Comments:

The modulators of channel function included in this table are those most commonly used to identify ryanodine-sensitive Ca^2+^ release pathways. Numerous other modulators of ryanodine receptor/channel function can be found in the reviews listed below. The absence of a modulator of a particular isoform of receptor indicates that the action of that modulator has not been determined, not that it is without effect. The potential role of cyclic ADP ribose as an endogenous regulator of ryanodine receptor channels is controversial. A region of RyR likely to be involved in ion translocation and selection has been identified [368, 1407].

## Transient Receptor Potential channels (TRP)


Ion channels→Voltage-gated ion channels→Transient Receptor Potential channels (TRP)

### Overview:

The TRP superfamily of channels (**nomenclature as agreed by NC-IUPHAR** [214, 1335]), whose founder member is the *Drosophila* Trp channel, exists in mammals as six families; TRPC, TRPM, TRPV, TRPA, TRPP and TRPML based on amino acid homologies. TRP subunits contain six putative TM domains and assemble as homo- or hetero-tetramers to form cation selective channels with diverse modes of activation and varied permeation properties (reviewed by [922]). Established, or potential, physiological functions of the individual members of the TRP families are discussed in detail in the recommended reviews and in a number of books [321, 515, 879, 1423]. The established, or potential, involvement of TRP channels in disease [1389] is reviewed in [603, 877], [881] and [621], together with a special edition of *Biochemica et Biophysica Acta* on the subject [877]. Additional disease related reviews, forpain [834], stroke [1401], sensation and inflammation [1241], itch [160], and airway disease [397, 1316], are available. The pharmacology of most TRP channels has been advanced in recent years. Broad spectrum agents are listed in the tables along with more selective, or recently recognised, ligands that are flagged by the inclusion of aprimary reference. See Rubaiy (2019) for a review of pharmacological tools for TRPC1/C4/C5 channels [1038]. Most TRP channels are regulated by phosphoinostides such as PtIns(4,5) P_2_ although the effects reported are often complex, occasionally contradictory, and likely to be dependent upon experimental conditions, such as intracellular ATP levels (reviewed by [882, 1031, 1254]). Such regulation is generally not included in the tables. When thermosensitivity is mentioned, it refers specifically to a high Q10 of gating, often in the range of 10-30, but does not necessarily imply that the channel’s function is to act as a ’hot’ or ’cold’ sensor. In general, the search for TRP activators has led to many claims for temperature sensing, mechanosensation, and lipid sensing. All proteins are of course sensitive to energies of binding, mechanical force, and temperature, but the issue is whether the proposed input is within a physiologically relevant range resulting in a response.

### TRPA (ankyrin) family

TRPA1 is the sole mammalian member of this group (reviewed by [373]). TRPA1 activation of sensory neurons contribute to nociception [552, 800, 1151]. Pungent chemicals such as mustard oil (AITC), allicin, and cinnamaldehyde activate TRPA1 by modification of free thiol groups of cysteine side chains, especially those located in its amino terminus [79, 465, 754, 756]. Alkenals with *α*, *β*-unsaturated bonds, such as propenal (acrolein), butenal (crotylaldehyde), and 2-pentenal can react with free thiols *via* Michael addition and can activate TRPA1. However, potency appears to weaken as carbon chain length increases [35, 79]. Covalent modification leads to sustained activation of TRPA1. Chemicals including carvacrol, menthol, and local anesthetics reversibly activate TRPA1 by non-covalent binding [571, 676, 1344, 1345]. TRPA1 is not mechanosensitive under physiological conditions, but can be activated by cold temperatures [259, 572]. The electron cryo-EM structure of TRPA1 [943] indicates that it is a 6-TM homotetramer. Each subunit of the channel contains two short ‘pore helices’ pointing into the ion selectivity filter, which is big enough to allow permeation of partially hydrated Ca^2+^ ions.

### TRPC (canonical) family

Members of the TRPC subfamily (reviewed by [5, 28, 86, 104, 361, 601, 938, 995]) fall into the subgroups outlined below. TRPC2 is a pseudogene in humans. It is generally accepted that all TRPC channels are activated downstream of G_q_/_11_-coupled receptors, or receptor tyrosine kinases (reviewed by [983, 1226, 1335]). A comprehensive listing of G protein-coupled receptors that activate TRPC channels is given in [5]. Hetero-oligomeric complexes of TRPC channels and their association with proteins to form signalling complexes are detailed in [28] and [602]. TRPC channels have frequently been proposed to act as store-operated channels (SOCs) (or components of multimeric complexes that form SOCs), activated by depletion of intracellular calcium stores (reviewed by [28, 82, 196, 197, 912, 950, 987, 1057, 1388]). However, the weight of the evidence is that they are not directly gated by conventional store-operated mechanisms, as established for Stim-gated Orai channels. TRPC channels are not mechanically gated in physiologically relevant ranges of force. All members of the TRPC family are blocked by 2-APB and SKF96365 [437, 438]. Activation of TRPC channels by lipids is discussed by [86]. Important progress has been recently made in TRPC pharmacology [118, 229, 370, 589, 824, 1038, 1094]. TRPC channels regulate a variety of physiological functions and are implicated in many human diseases [87, 119, 191, 378, 536, 723, 1143, 1168, 1280, 1296].

### TRPC1/C4/C5 subgroup

TRPC1 alone may not form a functional ion channel [276]. TRPC4/C5 may be distinguished from other TRP channels by their potentiation by micromolar concentrations of La^3^+. TRPC2 is a pseudogene in humans, but in other mammals appears to be an ion channel localized to microvilli of the vomeronasal organ. It is required for normal sexual behavior in response to pheromones in mice. It may also function in the main olfactory epithelia in mice [705, 910, 911, 1372, 1378, 1382, 1432].

### TRPC3/C6/C7 subgroup

All members are activated by diacylglycerol independent of protein kinase C stimulation [438].

### TRPM (melastatin) family

Members of the TRPM subfamily (reviewed by [356, 437, 950, 1413]) fall into the five subgroups outlined below.

### TRPM1/M3 subgroup

In darkness, glutamate released by the photoreceptors and ON-bipolar cells binds to the metabotropic glutamate receptor 6 , leading to activation of Go . This results in the closure of TRPM1. When the photoreceptors are stimulated by light, glutamate release is reduced, and TRPM1 channels are more active, resulting in cell membrane depolarization. Human TRPM1 mutations are associated with congenital stationary night blindness (CSNB), whose patients lack rod function. TRPM1 is also found melanocytes. Isoforms of TRPM1 may present in melanocytes, melanoma, brain, and retina. In melanoma cells, TRPM1 is prevalent in highly dynamic intracellular vesicular structures [507, 897]. TRPM3 (reviewed by [900]) exists as multiple splice variants which differ significantly in their biophysical properties. TRPM3 is expressed in somatosensory neurons and may be important in development of heat hyperalgesia during inflammation (see review [1196]). TRPM3 is frequently coexpressed with TRPA1 and TRPV1 in these neurons. TRPM3 is expressed in pancreatic beta cells as well as brain, pituitary gland, eye, kidney, and adipose tissue [899, 1195]. TRPM3 may contribute to the detection of noxious heat [1261].

### TRPM2

TRPM2 is activated under conditions of oxidative stress (respiratory burst of phagocytic cells). The direct activators are calcium, adenosine diphosphate ribose (ADPR) [1234] and cyclic ADPR (cADPR) [1385]. As for many ion channels, PI(4,5) P2 must also be present [1372]. Numerous splice variants of *TRPM2* exist which differ in their activation mechanisms [300]. Recent studies have reported structures of human (hs) TRPM2, which demonstrate two ADPR binding sites in hsTRPM2, one in the N-terminal MHR1/2 domain and the other in the C-terminal NUDT9-H domain. In addition, one Ca^2+^ binding site in the intracellular S2-S3 loop is revealed and proposed to mediate Ca^2+^ binding that induces conformational changes leading the ADPR-bound closed channel to open [495, 1287]. Meanwhile, a quadruple-residue motif (979FGQI982) was identified as the ion selectivity filter and a gate to control ion permeation in hsTRPM2 [1386]. TRPM2 is involved in warmth sensation [1089], and contributes to several diseases [94]. TRPM2 interacts with extra synaptic NMDA receptors (NMDAR) and enhances NMDAR activity in ischemic stroke [1430]. Activation of TRPM2 in macrophages promotes atherosclerosis [1406, 1431]. Moreover, silica nanoparticles induce lung inflammation in mice *via* ROS/PARP/TRPM2 signaling-mediated lysosome impairment and autophagy dysfunction [1289]. Recent studies have designed various compounds for their potential to selectively inhibit the TRPM2 channel, including ACA derivatives A23, and 2,3-dihydroquinazolin-4(1H)-one derivatives [1403, 1405].

### TrPm4/5 subgroup

TRPM4 and TRPM5 have the distinction within all TRP channels of being impermeable to Ca^2+^ [1335]. A splice variant of TRPM4 (*i.e.*TRPM4b) and TRPM5 are molecular candidates for endogenous calcium-activated cation (CAN) channels [413]. TRPM4 is active in the late phase of repolarization of the cardiac ventricular action potential. TRPM4 deletion or knockout enhances beta adrenergic-mediated inotropy [791]. Mutations are associated with conduction defects [519, 791, 1139]. TRPM4 has been shown to be an important regulator of Ca^2+^ entry in to mast cells [1243] and dendritic cell migration [65]. TRPM5 in taste receptor cells of the tongue appears essential for the transduction of sweet, amino acid and bitter stimuli [704] TRPM5 contributes to the slow afterdepolarization of layer 5 neurons in mouse prefrontal cortex [678]. Both TRPM4 and TRPM5 are required transduction of taste stimuli [311].

### TRPM6/7 subgroup

TRPM6 and 7 combine channel and enzymatic activities (‘chanzymes’) [212]. These channels have the unusual property of permeation by divalent (Ca^2+^, Mg^2+^, Zn^2+^) and monovalent cations, high single channel conductances, but overall extremely small inward conductance when expressed to the plasma membrane. They are inhibited by internal Mg^2+^ at ~0.6 mM, around the free level of Mg^2+^ in cells. Whether they contribute to Mg^2+^ homeostasis is a contentious issue. PIP_2_ is required for TRPM6 and TRPM7 activation [1044, 1340]. When either gene is deleted in mice, the result is embryonic lethality [541, 1330]. The C-terminal kinase region of TRPM6 and TRPM7 is cleaved under unknown stimuli, and the kinase phosphorylates nuclear histones [634, 635]. TRPM7 is responsible for oxidant- induced Zn^2+^ release from intracellular vesicles [4] and contributes to in-testinal mineral absorption essential for postnatal survival [826]. The putative metal transporter proteins CNNM1-4 interact with TRPM7 and regulate TRPM7 channel activity [58, 624].

### TRPM8

Is a channel activated by cooling and pharmacological agents evoking a ‘cool’ sensation and participates in the thermosensation of cold temperatures [81, 216, 272] reviewed by [615, 725, 773, 1255]. Direct chemical agonists include menthol and icilin[1349]. Besides, linalool can promote ERK phosphorylation in human dermal microvascular endothelial cells, down-regulate intracellular ATP levels, and activate TRPM8 [84]. Recent studies have found that TRPM8 has typical S4-S5 connectomes with clear selective filters and exowell rings [677], and have identified cryo-electron microscopy structures of mouse TRPM8 in closed, intermediate, and open states along the ligand- and PIP_2_-dependent gated pathways [1373]. Moreover, the last 36 amino acids at the carboxyl terminal of TRPM8 are key protein sequences for TRPM8’s temperature-sensitive function [232]. TRPM8 deficiency reduced the expression of S100A9 and increased the expression of HNF4*α* in the liver of mice, which reduced inflammation and fibrosis progression in mice with liver fibrosis, and helped to alleviate the symptoms of bile duct disease [720]. Channel deficiency also shortens the time of hypersensitivity reactions in migraine mouse models by promoting the recovery of normal sensitivity [19]. A cyclic peptide DeC-1.2 was designed to inhibit ligand activation of TRPM8 but not cold activation, which can eliminate the side effects of cold dysalgesia in oxaliplatin-treated mice without changing body temperature [14]. Analysis of clinical data shows that TRPM8-specific blockers WS12 can reduce tumor growth in colorectal cancer xenografted mice by reducing transcription and activation of Wnt signaling regulators and *β*-catenin and its target oncogenes, such as C-Myc and Cyclin D1 [924].

### TRPML (mucolipin) family

The TRPML family [225, 992, 998, 1348, 1393] consists of three mammalian members (TRPML1-3). TRPML channels are probably restricted to intracellular vesicles and mutations in the gene (*MCOLN1*) encoding TRPML1 (mucolipin-1) cause the neurodegenerative disorder mucolipidosis type IV (MLIV) in man. TRPML1 is a cation selective ion channel that is important for sorting/transport of endosomes in the late endocytotic pathway and specifically, fission from late endosome-lysosome hybrid vesicles and lysosomal exocytosis [1059]. TRPML2 and TRPML3 show increased channel activity in low luminal sodium and/or increased luminal pH, and are activated by similar small molecules [180, 403, 1138]. A naturally occurring gain of function mutation in TRPML3 (*i.e.* A419P) results in the varitint waddler (V*a*) mouse phenotype (reviewed by [883, 998]).

### TRPP (polycystin) family

The TRPP family (reviewed by [263, 265, 385, 471, 1327]) or PKD2 family is comprised of PKD2 (PC2), PKD2L1 (PC2L1), PKD2L2 (PC2L2), which have been renamed TRPP1, TRPP2 and TRPP3, respectively [1335]. It should also be noted that the nomenclature of PC2 was TRPP2 in old literature. However, PC2 has been uniformed to be called TRPP2 [436]. PKD2 family channels are clearly distinct from the PKD1 family, whose function is unknown. PKD1 and PKD2 form a hetero-oligomeric complex with a 1:3 ratio. [1161]. Although still being sorted out, TRPP family members appear to be 6TM spanning nonselective cation channels.

### TRPV (vanilloid) family

Members of the TRPV family (reviewed by [1244]) can broadly be divided into the non-selective cation channels, TRPV1-4 and the more calcium selective channels TRPV5 and TRPV6.

### TRPV1-V4 subfamily

TRPV1 is involved in the development of thermal hyperalgesia following inflammation and may contribute to the detection of noxius heat (reviewed by [973, 1140, 1178]). Numerous splice variants of TRPV1 have been described, some of which modulate the activity of TRPV1, or act in a dominant negative manner when co-expressed with TRPV1 [1086]. The pharmacology of TRPV1 channels is discussed in detail in [415] and [1259]. TRPV2 is probably not a thermosensor in man [933], but has recently been implicated in innate immunity [713]. Functional TRPV2 expression is described in placental trophoblast cells of mouse [244]. TRPV3 and TRPV4 are both thermosensitive. There are claims that TRPV4 is also mechanosensitive, but this has not been established to be within a physiological range in a native environment [153, 700].

### TRPV5/V6 subfamily

TRPV5 and TRPV6 are highly expressed in placenta, bone, and kidney. Under physiological conditions, TRPV5 and TRPV6 are calcium selective channels involved in the absorption and reabsorption of calcium across intestinal and kidney tubule epithelia (reviewed by [245, 342, 851, 1325]).TRPV6 is reported to play a key role in calcium transport in the mouse placenta [1323].

**Table:** 

Nomenclature	TRPA1
HGNC, UniProt	*TRPA1*, O75762
Chemical activators	Isothiocyanates (covalent) and 1,4-dihydropyridines (non-covalent)
Oxidative stress compounds	4-oxo-nonenal: pEC_50_ 5.7, H_2_O_2_, : pEC_50_ 3.6, hypochlorite: EC_50_ 11 ppm (human) and 7 ppm (mouse) (Mouse) [33, 95, 1067]
Physical activators	Cooling (<17 °C) (disputed) [552, 853, 1238]
Activators	polygodial (pEC_50_ 6.4) [328], acrolein (Agonist) (pEC_50_ 5.3) [physiological voltage] [79], allicin (Agonist) (pEC_50_ 5.1) [physiological voltage] [80], Δ^9^-tetrahydrocannabinol (Agonist) (pEC_50_ 4.9) [−60mV] [552], nicotine (non-covalent) (pEC_50_ 4.8) [−75mV] [1183], thymol (non-covalent) (pEC_50_ 4.7) Concentration range: 6.2×10^−6^M-2.5×10^−5^M [672], URB597 (Agonist) (pEC_50_ 4.6) [876], (−)-menthol (Partial agonist) (pEC_50_ 4–4.5) [571, 1339], allyl isothiocyanate (pEC_50_ 4.2) [465], cinnamaldehyde (Agonist) (pEC_50_ 4.2) [physiological voltage] [61] – Mouse, formalin (covalent. This level of activity is also observed for rat TRPA1) (pEC_50_ 3.4) [756, 800] – Mouse, icilin (Agonist) Concentration range: 1×10^−4^M [physiological voltage] [1151] – Mouse
Selective activators	JT010 (pEC_50_ 9.2) [1180], PF-4840154 (This compound has similar activity at rat and mouse TRPA1) (pEC_50_ 7.6) [1047], chlorobenzylidene malononitrile (covalent) (pEC_50_ 6.7) [131], ASP7663 (pEC_50_ 6.3) [622]
hannel blockers	AP18 (Inhibition) (pIC_50_ 5.5) [965], ruthenium red (Inhibition) (pIC_50_ 5.5) [−80mV] [853] – Mouse
Selective channel blockers	GDC-0334 (Inhibition) (pIC_50_ 8.8) [60], AM-0902 (Antagonist) (pIC_50_ 7.7) [1073], A-967079 (Inhibition) (pIC_50_ 7.2) [183]
Functional Characteristics	*γ* = 87–100 pS; conducts mono- and di-valent cations non-selectively (P_Ca_/P_Na_ = 0.84); outward rectification; activated by elevated intracellular Ca^2+^
Comments	miRNA-711 is a selective activator of TRPA1 (pEC_50_ ~5.0) [426]. GRC 17536 (structure not disclosed) is a TRPA1 antagonist with potential as an anti-tussive therapeutic [843]. Some pathogen-derived molecules activate human TRPA1, such as lipopolysaccharide (LPS) [806], indole (pEC_50_ 4.1) [210] and indole-3-carboxyaldehyde (pEC_50_ 4.1) [1367].

**Table:** 

Nomenclature	TRPC1	TRPC2	TRPC3	TRPC4
HGNC, UniProt	*TRPC1*, P48995	*TRPC2*, –	*TRPC3*, Q13507	*TRPC4*, Q9UBN4
Chemical activators	NO-mediated cysteine S-nitrosylation	Diacylglycerol (SAG, OAG, DOG): strongly inhibited by Ca^2+^/CaM once activated by DAG [1137]	diacylglycerols	NO-mediated cysteine S-nitrosylation, potentiation by extracellular protons
Physical activators	membrane stretch (likely direct)	DAG kinase; regulates DAG concentration in vomeronasal sensory neurons	–	–
Endogenous activators	–	Intracellular Ca^2+^	–	–
Activators	–	DOG (Agonist) Concentration range: 1×10^−4^M [−80mV] [741] – Mouse, SAG (Agonist) Concentration range: 1×10^−4^M [−80mV] [741] – Mouse	pyrazolopyrimidine 4n (pEC_50_ 7.7) [1002], GSK1702934A (Agonist) (pEC_50_ 7.1) [1352]	(−)-englerin A (Agonist) (pEC_50_ 7.9) [15], tonantzitlolone (pEC_50_ 6.9) [1040], La^3+^ (*μ*M range)
Channel blockers	2-APB (Antagonist) [−70mV] [1157], Gd^3+^(Antagonist) Concentration range: 2×10^−5^M [−70mV] [1429], La^3+^ (Antagonist) Concentration range: 1×10^−4^M [−70mV] [1157]	2-APB (Antagonist) Concentration range: 5×10^−5^M [−70mV – 80mV] [741 ] – Mouse, U73122 (Antagonist) Concentration range: 1×10^−5^M – Mouse	GSK2833503A (pIC_50_ 7.7) [80mV] [1089], GSK417651A (Antagonist) (pIC_50_ 7.1) [1304], Gd^3+^ (Antagonist) (pEC_50_ 7) [−60mV] [425], SAR7334 (pIC_50_ 6.6) [762], BTP2 (Antagonist) (pIC_50_ 6.5) [−80mV] [443], Pyr3 (pIC_50_ 6.2) [607], Pyr10 (Antagonist) (pIC_50_ 6.1) [1075], norgestimate (pK_i_ 5.5) [814], La^3+^ (Antagonist) (pIC_50_ 5.4) [−60mV] [425], clemizole (pIC_50_ 5) [1022], 2-APB (Antagonist) (pIC_50_ 5) [physiological voltage] [703], Ni^2+^, SKF96365	HC-070 (Antagonist) (pIC_50_ 7.3) [554], ML204 (pIC_50_ 5.5) [818], M084 (Inhibition) (pIC_50_ 5.3) [1424], clemizole (pIC_50_ 5.2) [1022], La^3+^ (mM range), SKF96365, niflumic acid (Antagonist) Concentration range: 3×10^−5^M [−60mV] [1273] – Mouse
Functional Characteristics	It is not yet clear that TRPC1 forms a homomer. It does form heteromers with TRPC4 and TRPC5	*γ* = 42 pS linear single channel conductance in 150 mM symmetrical Na^+^ in vomeronasal sensory neurons. P_Ca_/P_Na_ = 2.7; permeant to Na^+^, Cs^+^, Ca^2+^, but not NMDC [910, 1378]	*γ* = 66 pS; conducts mono and di-valent cations non-selectively (P_Ca_/P_Na_ = 1.6); monovalent cation current suppressed by extracellular Ca^2+^; dual (inward and outward) rectification	*γ* = 30 −41 pS, conducts mono and di-valent cations non-selectively (P_Ca_/P_Na_ = 1.1–77); dual (inward and outward) rectification

**Table:** 

Nomenclature	TRPC5	TRPC6	TRPC7
HGNC, UniProt	*TRPC5*, Q9UL62	*TRPC6*, Q9Y210	*TRPC7*, Q9HCX4
Chemical activators	NO-mediated cysteine S-nitrosylation (disputed), potentiation by extracellular protons	Diacylglycerols	diacylglycerols
Physical activators	Membrane stretch	Membrane stretch	–
Endogenous activators	intracellular Ca^2+^ (at negative potentials) (pEC_50_ 6.2), lysophosphatidylcholine	20-HETE, arachidonic acid, lysophosphatidylcholine	–
Activators	(−)-englerin A (Agonist) (pEC_50_ 8.1) [15], tonantzitlolone (pEC_50_ 7.1) [1040], BTD (pEC_50_ 5.8) [85], riluzole (pEC_50_ 5) [1023], methylprednisolone (pEC_50_ 4.9) [85], rosiglitazone (pEC_50_ 4.5) [768], Gd^3+^ Concentration range: 1×10^−4^M, La^3+^ (*μ*M range), Pb^2+^ Concentration range: 5×10^−6^M, genistein (independent of tyrosine kinase inhibition) [1328]	AM-0883 (Agonist) (pEC_50_ 7.3) [57], GSK1702934A (Agonist) (pIC_50_ 6.4) [1352], pyrazolopyrimidine 4n (pEC_50_ 5.9) [1002], OptoBI-1 (photoswitch activation; concentration range: 1-2x10^−5^M) [1209], OptoDArG (photoswitch activation; concentration range: 3x10^−5^M) [701], flufenamate, hyp 9 [685], hyperforin [686]	pyrazolopyrimidine 4n (pIC_50_ 6.1) [1002], OptoBI-1 (photoswitch activation; concentration range: 1-2x10^−5^M) [1209]
Selective activators	AM237 (pEC_50_ 7.7) [823]	–	–
Channel blockers	Pico145 (Inhibition) (pIC_50_ 8.9) [1039], HC-070 (Antagonist) (pIC_50_ 8) [554], AM12 (Inhibition) (pIC_50_ 6.6) [863], CFB-8438 (Inhibition) (pIC_50_ 6.5) [1384], galangin (pK_i_ 6.3) [863], clemizole (pIC_50_ 6) [1022], KB-R7943 (Inhibition) (pIC_50_ 5.9) [630], M084 (Inhibition) (pIC_50_ 5.1) [1424], ML204 (pIC_50_ ~5) [818], 2-APB (Antagonist) (pIC_50_ 4.7) [−80mV] [1350], La^3+^ (Antagonist) Concentration range: 5× 10^−3^M [−60mV] [553] – Mouse	AM-1473 (Antagonist) (pIC_50_ 9.7) [57], GSK2833503A (pIC_50_ 8.5) [80mV] [1089], GSK2332255B (Antagonist) (pIC_50_ 8.4) [1089], SAR7334 (pIC_50_ 8) [762], BTDM (Inhibition) (pIC_50_ 8) [1188], DS88790512 (Inhibition) (pIC_50_ ~79) [839], BI 749327 (Antagonist) (pIC_50_ 7.9) [706], SH045 (pIC_50_ 7.2) [424], larixyl acetate (Inhibition) (pIC_50_ 7) [1235], GSK417651A (Antagonist) (pIC_50_ 6.4) [1304], Pyrazolo-pyrimidine 14a (Inhibition) (pIC_50_ ~6) [279], clemizole (pIC_50_ 5.9) [1022], Gd^3+^ (Antagonist) (pIC_50_ 5.7) [−60mV] [506] – Mouse, SKF96365 (Antagonist) (pIC_50_ 5.4) [−60mV] [506] – Mouse, norgestimate (pIC_50_ 5.3) [814], La^3+^ (pIC_50_ ~5.2), amiloride (Antagonist) (pIC_50_ 3.9) [−60mV] [506] – Mouse, Cd^2+^ (Antagonist) (pIC_50_ 3.6) [−60mV] [506] – Mouse, 2-APB, ACAA, GsMTx-4, Extracellular H^+^, KB-R7943, ML9	SH045 (pIC_50_ ~77) [424], SAR7334 (pIC_50_ 6.7) [762], BI 749327 (Antagonist) (pIC_50_ 6.3) [706], larixyl acetate (Inhibition) (pIC_50_ ~6.3) [1235], 2-APB, La^3+^ (Antagonist) Concentration range: 1×10^−4^M [−60mV] [904] – Mouse, SKF96365 (Antagonist) Concentration range: 2.5×10^−5^M [−60mV] [904] – Mouse, amiloride
Selective channel blockers	AC1903 (Inhibition) (pIC_50_ 4.8) [1419]	–	–
Functional Characteristics	*γ* = 41-63 pS; conducts mono-and di-valent cations non-selectively (P_Ca_/P_Na_ = 1.8-9.5); dual rectification (inward and outward) as a homomer, outwardly rectifying when expressed with TRPC1 or TRPC4	*γ* = 28-37 pS; conducts mono and divalent cations with a preference for divalents (P_Ca_/P_Na_ = 4.5-5.0); monovalent cation current suppressed by extracellular Ca^2+^ and Mg^2+^, dual rectification (inward and outward), or inward rectification	*γ* = 25-75 pS; conducts mono and divalent cations with a preference for divalents (P_Ca_/P_Cs_ = 5.9); modest outward rectification (monovalent cation current recorded in the absence of extracellular divalents); monovalent cation current suppressed by extracellular Ca^2+^ and Mg^2+^

**Table:** 

Nomenclature	TRPM1	TRPM2
HGNC, UniProt	*TRPM1*, Q7Z4N2	*TRPM2*, O94759
Chemical activators	–	Agents producing reactive oxygen (e.g. H_2_O_2_) and nitrogen (e.g. GEA 3162) species
Physical activators	–	Heat ~ 35°C
Endogenous activators	pregnenolone sulphate [654]	intracellular cADPR (Agonist) (pEC_50_ 5) [−80mV – −60mV] [83, 623, 1211], intracellular ADP ribose (Agonist) (pEC_50_ 3.9–4.4) [−80mV] [961], intracellular Ca^2+^ (perhaps *via* calmodulin), H_2_O_2_, (Agonist) Concentration range: 5×10^−7^M-5×10^−5^M [physiological voltage] [358, 435, 632, 1124, 1310], membrane PIP, [1218], arachidonic acid (Potentiation) Concentration range: 1×10^−5^M-3×10^−5^M [physiological voltage] [435]
Activators	–	GEA 3162
Endogenous channel blockers	Zn^2+^ (pIC_50_ 6)	Zn^2+^ (pIC_50_ 6), extracellular H^+^
Channel blockers	–	2-APB (Antagonist) (pIC_50_ 6.1) [−60mV] [1212], ACAA (Antagonist) (pIC_50_ 5.8) [physiological voltage] [631], clotrimazole (Antagonist) Concentration range: 3×10^−6^M-3×10^−5^M [−60mV – −15mV] [462], econazole (Antagonist) Concentration range: 3×10^−6^M-3×10^−5^M [−60mV – −15mV] [462], flufenamic acid (Antagonist) Concentration range: 5×10^−5^M-1×10^−3^M [−60mV – −50mV] [461, 1212], miconazole (Antagonist) Concentration range: 1×10^−5^M [−60mV] [1212]
Functional Characteristics	Conducts mono- and di-valent cations non-selectively, dual rectification (inward and outward)	*γ* = 52-60 pS at negative potentials, 76 pS at positive potentials; conducts mono- and di-valent cations non-selectively (P_Ca_/P_Na_ = 0.6-0.7); non-rectifying; inactivation at negative potentials; activated by oxidative stress probably *via* PARP-1, PARP inhibitors reduce activation by oxidative stress, activation inhibited by suppression of APDR formation by glycohydrolase inhibitors.
Comments	–	Additional endogenous activators include 2’-deoxy-ADPR, 3’-deoxy-ADPR, 2’-phospho-ADPR, 2-F-ADPR and ADP-ribose-2’-phosphate (ADPRP) [357, 1219]. 8-Br-cADPR acts as a gating inhibitor [623].

**Table:** 

Nomenclature	TRPM3	TRPM4
HGNC, UniProt	*TRPM3*, Q9HCF6	*TRPM4*, Q8TD43
Other channel blockers	–	Intracellular nucleotides including ATP, ADP, adenosine 5’-monophosphate and AMP-PNP with an IC_50_ range of 1.3-1.9 *μ*M
Physical activators	heat (Q_10_ = 7.2 between 15-25°C), hypotonic cell swelling [405, 1261, 1262]	Membrane depolarization (V_1/2_ = −20 mV to + 60 mV dependent upon conditions) in the presence of elevated [Ca^2+^]_i_, heat (Q_10_ = 8.5 @ +25 mV between 15 and 25°C)
Endogenous activators	pregnenolone sulphate (pEC_50_ 4.9) [1266], sphingosine (Agonist) (pEC_50_ 4.9) [physiological voltage] [406], dihydrosphingosine (Agonist) (pEC_50_ 4.7) [406], epipregnanolone sulphate [767]	intracellular Ca^2+^ (Agonist) (pEC_50_ 3.9-6.3) [−100mV – 100mV] [880, 884, 885, 1181]
Activators	CIM0216 (pEC_50_ 6.1) [448, 1196], nifedipine, pentafluoro-trityl clotrimazole analogue 29a (Agonist) [558]	BTP2 (Agonist) (pEC_50_ 8.1) [−80mV] [1181], decavanadate (Agonist) (pEC_50_ 5.7) [−100mV] [884]
Gating inhibitors	2-APB (Antagonist) (pIC_50_ 4) [1350]	flufenamic acid (Antagonist) (pIC_50_ 5.6) [100mV] [1234] – Mouse, clotrimazole (Antagonist) Concentration range: 1×10^−6^M-1×10^−5^M [100mV] [888]
Endogenous channel blockers	Mg^2+^ (Antagonist) (pIC_50_ 2) [898] – Mouse, extracellular Na^+^ (TRPM3*α*2 only)	–
Channel blockers	isosakuranetin (pIC_50_ 6.3) [1152], primidone (pIC_50_ 6.2) [643], maprotiline (pIC_50_ 5.8) [643], diclofenac (pIC_50_ 5.2) [1172], liquiritigenin (pIC_50_ 5.2) [1152], naringenin (pIC_50_ 5.2) [1152, 1153], Gd^3+^ (Antagonist) (pIC_50_ 4) [405, 671 ], La^3+^ (Antagonist) (pIC_50_ 4) [405, 671], chloroform (Antagonist) (pIC_50_ 3.8) [582], halothane (Antagonist) (pIC_50_ 3.3) [582]	compound 6 (pIC_50_ 6.4) [1148], LBA (pIC_50_ 5.8) [1148], compound 5 (Antagonist) (pIC_50_ 5.7) [923], meclofenamic acid (pIC_50_ 5.5) [1239], 9-phenanthrol (pIC_50_ 4.6–4.8) [398], spermine (Antagonist) (pIC_50_ 4.2) [100mV] [886], adenosine (pIC_50_ 3.2)
Functional Characteristics	TRPM3_1235_: *γ* = 83 pS (Na^+^ current), 65 pS (Ca^2+^ current); conducts mono and di-valent cations non-selectively (P_Ca_/P_Na_ = 1.6) TRPM3*α*1: selective for monovalent cations (P_Ca_/P_Cs_~0.1); TRPM3*α*2: conducts mono- and di-valent cations non-selectively (P_Ca_/P_Cs_ = 1-10); In- and outwardly rectifying currents by co-application of pregnenolone sulphate and clotrimazole or single application of CIM0216 [448, 1260]. Activated by clotrimazole but not by pregnenolone sulphate [447].	*γ* = 23 pS (within the range 60 to +60 mV); permeable to monovalent cations; impermeable to Ca^2+^; strong outward rectification; slow activation at positive potentials, rapid deactivation at negative potentials, deactivation blocked by decavanadate
Comments	G protein *βγ* subunits can act as endogenous inhibitors of TRPM3 channel activity [54, 266, 1003].	–

**Table:** 

Nomenclature	TRPM5	TRPM6
HGNC, UniProt	*TRPM5*, Q9NZQ8	*TRPM6*, Q9BX84
EC number	–	2.7.11.1
Other chemical activators	–	constitutively active, activated by reduction of intracellular Mg^2+^
Physical activators	membrane depolarization (V_1/2_ = 0 to + 120 mV dependent upon conditions), heat (Q_10_ = 10.3 @ −75 mV between 15 and 25°C)	–
Endogenous activators	intracellular Ca^2+^ (Agonist) (pEC_50_ 4.5-6.2) [−80mV – 80mV] [473, 717, 1234] – Mouse	extracellular H^+^ (Potentiation), intracellular Mg^2+^
Activators	compound 39 (Agonist) (pEC_50_ 7.5) [1048]	2-APB (Agonist) (pEC_50_ 3.4–3.7) [−120mV – 100mV] [692]
Endogenous channel blockers	–	Mg^2+^ (inward current mediated by monovalent cations is blocked) (pIC_50_ 5.5–6), Ca^2+^ (inward current mediated by monovalent cations is blocked) (pIC_50_ 5.3–5.3)
Channel blockers	flufenamic acid (pIC_50_ 4.6), intracellular spermine (pIC_50_ 4.4), Extracellular H^+^ (pIC_50_ 3.2)	ruthenium red (pIC_50_ 7) [*voltage dependent* -120mV]
Allosteric modulators	APV207095A (Potentiation) (pEC_50_ 5) [1250], APV207094A (Potentiation) (pEC_50_ 4.4) [1250], APV207010A (Potentiation) (pEC_50_ 4.4) [1250], APV206690A (Potentiation) (pEC_50_ 4) [1250]	–
Functional Characteristics	*γ* = 15-25 pS; conducts monovalent cations selectively (P_Ca_/P_Na_ = *0.05*); strong outward rectification; slow activation at positive potentials, rapid inactivation at negative potentials; activated and subsequently desensitized by [Ca^2+^]I	*γ* = 40-87 pS; permeable to mono- and di-valent cations with a preference for divalents (Mg^2+^ > Ca^2+^; P_Ca_/P_Na_ = 6.9), conductance sequence Zn^2+^ > Ba^2+^ > Mg^2+^= Ca^2+^ = Mn^2+^ > Sr^2+^ > Cd^2+>^ Ni^2+^; strong outward rectification abolished by removal of extracellular divalents, inhibited by intracellular Mg^2+^ (IC_S0_ = 0.5 mM) and ATP
Comments	TRPM5 is not blocked by ATP. APV206512A and APV206513A are TRMP5 blockers, with IC_50_s of 15 *μ*M [1250]. Steviol glycosides (sweet-tasting organic molecules) act as positive modulators of TRMP5 activity [967].	–

**Table:** 

Nomenclature	TRPM7	TRPM8
HGNC, UniProt	*TRPM7*, Q96QT4	*TRPM8*, Q7Z2W7
EC number	2.7.11.1	–
Chemical activators	–	agonist activities are temperature dependent and potentiated by cooling
Physical activators	–	depolarization (V_1/2_ ~ +50 mV at 15°C), cooling (< 22-26°C)
Endogenous activators	Extracellular H^+^ (Potentiation) (pEC_50_ 4.5) [537]	–
Activators	2-APB Concentration range: >1×10^−3^M [692] – Mouse, naltriben [474]	icilin (Agonist) (pEC_50_ 6.7–6.9) [physiological voltage] [32, 88] – Mouse, tacrolimus (Agonist) (pEC_50_4.8) [40], (−)-menthol (inhibited by intracellular Ca^2+^) (pEC_50_ 4.6) [−120mV – 160mV] [1253]
Selective activators	–	WS-12 (Full agonist) (pEC_50_ 4.9) [physiological voltage] [750, 1101] – Rat
Selective antagonists	–	KPR-5714 (pIC_50_ 7.6) [859]
Channel blockers	sphingosine (Inhibition) (pIC_50_ 6.2) [−100mV – 100mV] [1001] – Mouse, fingolimod (Inhibition) (pIC_50_ 6.1) [−100mV – 100mV] [1001] – Mouse, spermine (Inhibition) (pK_i_ 5.6) [−110mV – 80mV] [629] – Rat, 2-APB (Inhibition) (pIC_50_ 3.8) [−100mV – 100mV] [692] – Mouse, carvacrol (Inhibition) (pIC_50_ 3.5) [−100mV – 100mV] [936] – Mouse, Mg^2+^ (Antagonist) (pIC_50_ 2.5) [80mV] [852] – Mouse, La^3+^ (Antagonist) Concentration range: 2×10^−3^M [−100mV – 100mV] [1045] – Mouse	BCTC (Antagonist) (pIC_50_ 6.1) [physiological voltage] [88] – Mouse, scutellarein (pIC_50_ 5.8) [1062], 2-APB (Antagonist) (pIC_50_ 4.9–5.1) [100mV – −100mV] [489, 864] – Mouse, capsazepine (Antagonist) (pIC_50_ 4.7) [physiological voltage] [88] – Mouse
Selective channel blockers	–	PF-05105679 (Antagonist) (pIC_50_ 7) [*voltage dependent*] [36]
Functional Characteristics	*γ* = 40-105 pS at negative and positive potentials respectively; conducts mono-and di-valent cations with a preference for monovalents (P_Ca_/P_Na_ = 0.34); conductance sequence Ni^2+^ > Zn^2+^ > Ba^2+^ = Mg^2+^ > Ca^2+^ = Mn^2+^ > Sr^2+^ > Cd^2+^; outward rectification, decreased by removal of extracellular divalent cations; inhibited by intracellular Mg^2+^, Ba^2+^, Sr^2+^, Zn^2+^, Mn^2+^ and Mg.ATP (disputed); activated by and intracellular alkalinization; sensitive to osmotic gradients	*γ* = 40-83 pS at positive potentials; conducts mono- and di-valent cations non-selectively (P_Ca_/P_Na_ = 1.0-3.3); pronounced outward rectification; demonstrates densensitization to chemical agonists and adaptation to a cold stimulus in the presence of Ca^2+^; modulated by lysophospholipids and PUFAs
Comments	2-APB acts as a channel blocker in the *μ*M range. Recent study shows cAMP inhibits TRPM7-mediated Ca^2+^ influx [130]. Waixenicin-A specifically inhibits TRPM7 [1427].	Cannabidiol and A9-tetrahydrocannabinol are examples of cannabinoid activators. TRPM8 is insensitive to ruthenium red. Icilin requires intracellular Ca^2+^ for full agonist activity.

**Table:** 

Nomenclature	TRPML1	TRPML2	TRPML3
HGNC, UniProt	*MCOLN1*, Q9GZU1	*MCOLN2*, Q8IZK6	*MCOLN3*, Q8TDD5
Endogenous activators	phosphatidyl (3,5) inositol bisphosphate (Also activates other TRPMLs) (pEC_50_ 7.3) [289]	–	–
Activators	ML SA1 (pEC_50_ 7.3) [−140mV] [1098], MK6-83 (pEC_50_ 7) [182], SF-22 (pEC_50_ 6.3) [−200mV] [182], ML-SA5 (pEC_50_ 5.3) [1383], SF-51 (pEC_50_ 4.5) [1098], ML1-SA1 [1138] TRPML1^Va^: Constitutively active, current potentiated by extra-cellular acidification (equivalent to intralysosomal acidification)	ML SA1 Concentration range: 1×10^−5^M [−140mV] [1098], phosphatidyl (3,5) inositol bisphosphate Concentration range: 1×10^−6^M [−140mV] [289] TRPML2^Va^: Constitutively active, current potentiated by extracellular acidification (equivalent to intralysosomal acidification)	SF-11 (pEC_50_ 6.6) [404], EVP-21 (pEC_50_ 4.3) [1017], ML SA1 Concentration range: 1×10^−5^M [−140mV] [1098], phosphatidyl (3,5) inositol bisphosphate Concentration range: 1×10^−6^M [−140mV] [289]
Selective activators	–	ML2-SA1 (Agonist) (pEC_50_ 1.2) [984]	ML3-SA1 (pEC_50_ 9) [1138]
Channel blockers	estradiol 3-methyl ether (pIC_50_ 6.7) [1042], PRU-12 (Inhibition) (pIC_50_ 6.6) [1042], estradiol 3-methyl ether (pIC_50_ 6.2) [1042]	–	Cd^3+^ (Antagonist) (pIC_50_ 4.7) [−80mV] [854] – Mouse
Functional Characteristics	TRPML1^Va^: *γ* = 40 pS and 76-86 pS at very negative holding potentials with Fe^2+^ and monovalent cations as charge carriers, respectively; conducts Na^+^s≅ K^+^>Cs^+^ and divalent cations (Ba^2+^> Mn^2+^>Fe^2+^>Ca^2+^> Mg^2+^> Ni^2+^>Co^2+^> Cd^2+^>Zn^2+^≫Cu^2+^); monovalent cation flux suppressed by divalent cations (*e.g.* Ca^2+^, Fe^2+^); inwardly rectifying	Conducts Na^+^; monovalent cation flux suppressed by divalent cations; inwardly rectifying	TRPML3^Va^: *γ* = 49 pS at very negative holding potentials with monovalent cations as charge carrier; conducts Na^+^ > K^+^ > Cs^+^ with maintained current in the presence of Na^+^, conducts Ca^2+^ and Mg^2+^, but not Fe^2+^, impermeable to protons; inwardly rectifying Wild type TRPML3: *γ* = 59 pS at negative holding potentials with monovalent cations as charge carrier; conducts Na^+^ > K^+^ > Cs^+^ and Ca^2+^ (P_Ca_/P_K_≅ 350), slowly inactivates in the continued presence of Na^+^ within the extracellular (extracytosolic) solution; outwardly rectifying
Comments	TRPML1 current is potentiated by acidic pH and sphingosine [1098].	TRPML2 current is inhibited by intralysosomal acidification [984].	Current is activated by Na^+^-free extracellular (extracytosolic) solution, and is inhibited by extracellular acidification (equivalent to intra-lysosomal acidification). Channel blockers include the ML-SI series of compounds (*e.g.* ML-SI1; concentration range 5x10^−5^ M; −120mV [807].

**Table:** 

Nomenclature	TRPP1	TRPP2	TRPP3
HGNC, UniProt	*PKD2*, Q13563	*PKD2L1*, Q9P0L9	*PKD2L2*, Q9NZM6
Activators	–	Calmidazolium (in primary cilia): 10 *μ*M	–
Channel blockers	–	phenamil (pIC_50_ 6.9), benzamil (pIC_50_ 6), ethylisopropylamiloride (pIC_50_ 5), amiloride (pIC_50_ 3.8), Gd^3+^ Concentration range: 1×10^−4^M [−50mV] [192], La^3+^ Concentration range: 1×10^−4^M [−50mV] [192], flufenamate	
Functional Characteristics	TRPP1 (PKD2) forms a cation channel (as a homomer consisting of 4 PKD2 sub-units or as a heteromer combining 3 PKD2 subunits with one PKD1 subunit) that is expressed on primary cilia of kidney epithelial cells [722]. In kidney epithelial cells TRPP1 is only functional in the ciliary membrane, but not in the plasma membrane. In oocyte overexpression TRPP1 forms functional homomeric and heteromeric channels. Gain of function mutations in TRPP1 in either the S4-S5 linker (F604P) or in the lower gate (L677A, N681A) result in constitutively active channels [41,420, 1301]. TRPP1 prefers monovalent cations over divalent cations in the order of K^+^>Na^+^>Ca^2+^ (permeability 1:0.4:0.025), showing low selectivity for Ca^2+^. The conductance of TRPP1 varies depending on the ion (K^+^ : 144 pS, Na^+^: 89 pS, Ca^2+^: 4pS) [722]. TRPP1 homomeric channel produces a larger conductance of 82 pS than the PC-1/TRPP1 heteromeric channel (79.5 pS) with higher absolute open probability (TRPP1 homomeric channel: 0.58, PC-1/TRPP1 heteromeric channel: 0.08) in primary cilia [420]. Specific activators or channel blockers of TRPP1 remain unknown.	TRPP2 is a nonselective cation channel functionally expressed on primary cilia and/or the plasma membrane depending on cell type. It can form a functional channel with PC1-L1 on primary cilia of retinal pigmented epithelial cells. TRPP2 (PKD2L1) displays calcium dependent activation. Calcium accumulation due to prolonged channel activity may lead to outward-moving Ca^2+^ ions within the pore to close the channel [249]. TRPP2 permeates K^+^, Na^+^ and Ca^2+^ with the single-channel conductance of 189 pS for K^+^, 156 for Na^+^, and 53 pS for Ca^2+^, respectively [327]. PKD2L1 forms a heteromeric channel with PC-1 L3 (*PKD1L3*) that may be activated by intracellular Ca^2+^ [1160].	TRPP3 is not fully characterized yet. One report suggests the single channel activity of PKD2L2 in HEK293 cells as a 25 pS conductance [1171], but these recordings have not been confirmed.
Comments	Several studies have reported that TRPP1 forms heteromeric ion channels with other TRP channels such as TRPM3 and TRPM4, but the physiological significance of these potential heteromers remains unclear [355, 609]. TRPP1 has also been reported to function as a heteromeric channel with PC-1-L1 (*PKD1L1*) in the embryonic node, but the biophysical characteristics of this heteromeric channel have not yet been characterized [579].	–	–

**Table:** 

Nomenclature	TRPV1	TRPV2
HGNC, UniProt	*TRPV1*, Q8NER1	*TRPV2*, Q9Y5S1
Other chemical activators	NO-mediated cysteine S-nitrosylation	–
Physical activators	depolarization (V_1/2_, ~ 0 mV at 35°C), noxious heat (> 43°C at pH 7.4)	–
Endogenous activators	extracellular H^+^ (at 37°C) (pEC_50_ 5.4), 12S-HPETE (Agonist) (pEC_50_ 5.1) [−60mV] [502] – Rat, anandamide (pEC_50_ 5) [11], LTB_4_ (Agonist) (pEC_50_ 4.9) [−60mV] [502] – Rat, 5S-HETE	–
Activators	resiniferatoxin (Agonist) (pEC_50_ 8.4) [physiological voltage] [1120], capsaicin (Agonist) (pEC_50_ 7.5) [−100mV – 160mV] [1253], RhTx (pEC_50_ 6.3) [1363], piperine (Agonist) (pEC_50_ 4.4–5) [−70mV] [801], camphor, diphenylboronic anhydride, phenylacetylrinvanil [38]	2-APB (pEC_50_ 5) [866, 1000] – Rat, A9-tetrahydrocannabinol (pEC_50_ 4.8) [1000] – Rat, cannabidiol (pEC_50_ 4.5) [1000], probenecid (pEC_50_ 4.5) [62] – Rat, 2-APB (Agonist) (pEC_50_ 3.8–3.9) [physiological voltage] [489, 555] – Mouse, diphenylboronic anhydride (Agonist) Concentration range: 1×10^−4^M [−80mV] [208, 555] – Mouse
Selective activators	olvanil (Agonist) (pEC_50_ 7.7) [physiological voltage] [1120], DkTx (pEC_50_ 6.6) [physiological voltage] [117] – Rat	–
Channel blockers	5’-iodoresiniferatoxin (pIC_50_ 8.4), 6-iodo-nordihydrocapsaicin (pIC_50_ 8), AMG 9810 (Inhibition) (pIC_50_ 7.8) [physiological voltage] [379], BCTC (Antagonist) (pIC_50_ 7.5) [174], capsazepine (Antagonist) (pIC_50_ 7.4) [−60mV] [798], ruthenium red (pIC_50_ 6.7–7)	ruthenium red (pIC_50_ 6.2), tranilast (Inhibition) (pIC_50_ 4–5) [871], SKF96365 (pIC_50_ 4) [555], TRIM (Inhibition) Concentration range: 5×10^−4^M [555] – Mouse
Selective channel blockers	AMG517 (pIC_50_ 9) [111], AMG628 (pIC_50_ 8.4) [1283] – Rat, A425619 (pIC_50_ 8.3) [320], A778317 (pIC_50_ 8.3) [100], SB366791 (pIC_50_ 8.2) [417], JYL1421 (Antagonist) (pIC_50_ 8) [1298] – Rat, JNJ17203212 (Antagonist) (pIC_50_ 7.8) [physiological voltage] [1174], SB452533 (Antagonist) (pK_B_ 7.7), SB705498 (Antagonist) (pIC_50_ 7.1) [416]	SET2 (pIC_50_ 6.3) [168], loratadine (Inhibition) (pIC_50_ 5.5) [1236]
Allosteric modulators (Positive)	s-RhTx (pEC_50_ 6.1) [1402], MRS-1477 [578]	–
Labelled ligands	[^3^H]A778317 (Channel blocker) (pK_d_ 8.5) [100], [^125^I]resiniferatoxin (Channel blocker, Antagonist) (pIC_50_ 8.4) [−50mV] [1267] – Rat, [^3^H]resiniferatoxin (Activator)	–
Functional Characteristics	*γ* = 35 pS at − 60 mV; 77 pS at + 60 mV, conducts mono and di-valent cations with a selectivity for divalents (P_Ca_/P_Na_ = 9.6); voltage- and time- dependent outward rectification; potentiated by ethanol; activated/potentiated/upregulated by PKC stimulation; extracellular acidification facilitates activation by PKC; desensitisation inhibited by PKA; inhibited by Ca^2+^/calmodulin; cooling reduces vanilloid-evoked currents; may be tonically active at body temperature	Conducts mono- and di-valent cations (P_Ca_/P_Na_ = 0.9-2.9); dual (inward and outward) rectification; current increases upon repetitive activation by heat; translocates to cell surface in response to IGF-1 to induce a constitutively active conductance, translocates to the cell surface in response to membrane stretch. Cannabidiol sensitizes TRPV2 channels to activation by 2APB [389].

**Table:** 

Nomenclature	TRPV3	TRPV4
HGNC, UniProt	*TRPV3*, Q8NET8	*TRPV4*, Q9HBA0
Other chemical activators	NO-mediated cysteine S-nitrosylation	Epoxyeicosatrieonic acids and NO-mediated cysteine S-nitrosylation
Physical activators	depolarization (V_1/2_ ~ +80 mV, reduced to more negative values following heat stimuli), heat (23°C - 39°C, temperature threshold reduces with repeated heat challenge)	Constitutively active, heat (> 24°C - 32°C), mechanical stimuli
Activators	incensole acetate (pEC_50_ 4.8) [841] – Mouse, 2-APB (Full agonist) (pEC_50_ 4.6) [−80mV – 80mV] [209] – Mouse, diphenylboronic anhydride (Full agonist) (pEC_50_ 4.1–4.2) [*voltage dependent* −80mV – 80mV] [208] – Mouse, thymol (Full agonist) (pEC_50_ 3.3) [1345] – Mouse, eugenol (Full agonist) (pEC_50_ 2.5) [1345] – Mouse, camphor (Full agonist) (pEC_50_ 2) [833] – Mouse, (−)-menthol (pEC_50_ 1.7) [−80mV – 80mV] [755] – Mouse, carvacrol (Full agonist) Concentration range: 5×10^−4^M [−80mV – 80mV] [1345] – Mouse	phorbol 12-myristate 13-acetate (Agonist) (pEC_50_ 7.9) [physiological voltage] [1343], quinazolin-4(3H) derivative 36 (pEC_50_ 7.2) [48], curcumin (pEC_50_ 5.2) [1093], arachidonic acid (pEC_50_ 5) [1305] – Mouse, puerarin (pEC_50_ 4.8) [1418], vildagliptin (pEC_50_ 3) [369]
Selective activators	KS0365 (pEC_50_ 5.3) [761], 6-tert-butyl-*m*-cresol (pEC_50_ 3.4) [1257] – Mouse	GSK1016790A (pEC_50_ 8.7) [physiological voltage] [1208], 4*α*-PDH (pEC_50_ 7.1) [physiological voltage] [608] – Mouse, 4*α*-PDD (Agonist) (pEC_50_ 6.5) [1343], RN1747 (pEC_50_ 6.1) [physiological voltage] [1249], bisandrographolide (Agonist) (pEC_50_ 6) [−60mV] [1125] – Mouse
Inhibitors	–	GSK2798745 (pIC_50_ 8.8) [133], GSK-Bz derivative 2b (pIC_50_ 7.7) [13], paracetamol (pIC_50_ 6.5) [856], cimifugin (pIC_50_ 5.8) [1356], propofol (pIC_50_ 4.4) [1278], Crotamiton [606]
Channel blockers	forsythoside B (Inhibition) (pIC_50_ 6.2) [1404], ruthenium red (Inhibition) (pIC_50_ 6) [952] – Mouse, diphenyltetrahydrofuran (Antagonist) (pIC_50_ 5–5.2) [−80mV – 80mV] [208] – Mouse, Citrusinine II (Inhibition) (pIC_50_ 4.9) [427], osthole (Inhibition) (pIC_50_ 4.4) [1167]	NSC151066 (Inhibition) (pIC_50_ 6.8) [287], ruthenium red (Inhibition) (pIC_50_ 6.7) [414] – Rat, Gd^3+^, La^3+^
Selective channel blockers	Trpvicin (Inhibition) (pIC_50_ 6.4) [338], Isochlorogenic acid B (Inhibition) (pIC_50_ 6.1) [997], Isochlorogenic acid A (Inhibition) (pIC_50_ 5.6) [997], verbascoside (Inhibition) (pIC_50_4.8) [1166]	HC067047 (Inhibition) (pIC_50_ 7.3) [−40mV] [335], RN-9893 (Antagonist) (pIC_50_ 6.2) [1312], RN1734 (Inhibition) (pIC_50_ 5.6) [physiological voltage] [1249]
Functional Characteristics	*γ* = 197 pS at = +40 to +80 mV, 48 pS at negative potentials; conducts mono- and di-valent cations; outward rectification; potentiated by arachidonic acid	*γ* = ~60 pS at −60 mV, −90-100 pS at +60 mV; conducts mono- and di-valent cations with a preference for divalents (P_Ca_/P_N_ =6-10); dual (inward and outward) rectification; potentiated by intracellular Ca^2+^ *via* Ca^2+^/calmodulin; inhibited by elevated intracellular Ca^2+^ *via* an unknown mechanism (IC_50_ = 0.4 *μ*M)

**Table:** 

Nomenclature	TRPV5	TRPV6
HGNC, UniProt	*TRPV5*, Q9NQA5	*TRPV6*, Q9H1D0
Other channel blockers	Pb^2+^ = Cu^2+^ = Gd^3+^ > Cd^2+^ > Zn^2+^ > La^3+^ > Co^2+^ > Fe^2^	–
Activators	constitutively active (with strong buffering of intracellular Ca^2+^)	acetaldehyde (pEC_50_ 6.7) [803], ethanol (pEC_50_ 0.8) [803], 2-APB (Potentiation) constitutively active (with strong buffering of intracellular Ca^2+^)
Inhibitors	gentamicin (pIC_50_ 6) [1237], tetrahydrocannabivarin (pIC_50_ 5.4) [527], oxoglaucine (pIC_50_ 4.8) [1414]	GSK3527497 (pIC_50_ 7.9) [132], SOR-C13 (pIC_50_ 7.8) [124], TRPV6 inhibitor cis-22 a (pIC_50_ 6.5) [1112], tetrahydrocannabivarin (pIC_50_ 5.1) [527]
Channel blockers	ruthenium red (pIC_50_ 6.9), Mg^2+^	ruthenium red (Antagonist) (pIC_50_ 5) [−80mV] [470] – Mouse, Cd^2+^, La^3+^, Mg^2+^
Functional Characteristics	*γ* = 59-78 pS for monovalent ions at negative potentials, conducts mono- and di-valents with high selectivity for divalents (P_Ca_/P_Na_ > 107); voltage- and time-dependent inward rectification; inhibited by intracellular Ca^2+^ promoting fast inactivation and slow downregulation; feedback inhibition by Ca^2+^ reduced by calcium binding protein 80-K-H; inhibited by extracellular and intracellular acidosis; upregulated by 1,25-dihydrovitamin D3	*γ* = 58-79 pS for monovalent ions at negative potentials, conducts mono- and di-valents with high selectivity for divalents (P_Ca_/P_Na_ > 130); voltage- and time-dependent inward rectification; inhibited by intracellular Ca^2+^ promoting fast and slow inactivation; gated by voltage-dependent channel blockade by intracellular Mg^2+^; slow inactivation due to Ca^2+^-dependent calmodulin binding; phosphorylation by PKC inhibits Ca^2+^-calmodulin binding and slow inactivation; upregulated by 1,25-dihydroxyvitamin D3

### Comments:

#### TRPA (ankyrin) family

Agents activating TRPA1 in a covalent manner are thiol reactive electrophiles that bind to cysteine and lysine residues within the cytoplasmic domain of the channel [465, 753]. TRPA1 is activated by a wide range of endogenous and exogenous compounds and only a few representative examples are mentioned in the table: an exhaustive listing can be found in [64]. In addition, TRPA1 is potently activated by intracellular zinc (EC_50_ = 8 nM) [34, 488]. A gain-of-function mutation in TRPA1 was found to cause familial episodic pain syndrome [639].

#### TRPM (melastatin) family

Ca^2+^ activates all splice variants of TRPM2, but other activators listed are effective only at the full length isoform [300]. Inhibition of TRPM2 by clotrimazole, miconazole, econazole, flufenamic acid is largely irreversible. Co-application of pregnenolone sulphate and clotrimazole caused TRPM3 currents to acquire an inwardly rectifying component at negative voltages, resulting in a biphasic conductance-voltage relationship. Evidence was presented that the inward current might reflect the permeation of cations through the opening of a non-canonical pore [1260]. TRPM3 activity is impaired in chronic fatigue syndrome/myalgic encephalomyelitis patients suggesting changes in intracellular Ca^2+^ concentration, which may impact natural killer cellular functions [144]. TRPM4 exists as multiple spice variants: data listed are for TRPM4b. The sensitivity of TRPM4b and TRPM5 to activation by [Ca^2+^]_i_ demonstrates a pronounced and time-dependent reduction following excision of inside-out membrane patches [1234]. The V_1/2_ for activation of TRPM4 and TRPM5 demonstrates a pronounced negative shift with increasing temperature. Activation of TRPM8 by depolarization is strongly temperature-dependent via a channel-closing rate that decreases with decreasing temperature. The V_1/2_ is shifted in the hyperpolarizing direction both by decreasing temperature and by exogenous agonists, such as (1)-menthol [1253] whereas antagonists produce depolarizing shifts in V_1/2_ [772]. The V_1/2_ for the native channel is far more positive than that of heterologously expressed TRPM8 [772]. It should be noted that (−)-menthol and structurally related compounds can elicit release of Ca^2+^ from the endoplasmic reticulum independent of activation of TRPM8 [760]. Intracellular pH modulates activation of TRPM8 by cold and icilin, but not (−)-menthol [32].

#### TRPML (mucolipin) family

Data in the table are for TRPML proteins mutated (*i.e* TRPML-1^Va^, TRPML2^Va^ and TRPML3^Va^) at loci equivalent to TRPML3 A419P to allow plasma membrane expression when expressed in HEK-293 cells and subsequent characterisation by patch-clamp recording [288, 401, 594, 854, 1346]. Data for wild type TRPML3 are also tabulated [594, 595, 854, 1346]. It should be noted that alternative methodologies, particularly in the case of TRPML1, have resulted in channels with differing biophysical characteristics (reviewed by [992]). Initial functional characteristics of TRPML channels are performed on their Va mutations of TRPMLs at loci equivalent to TRPML3 A419P. Current pharmacological characterization of channel activators and blockers are conducted on wild-type channel proteins using endolysosomal patch-clamp [182, 289, 984, 1098].

#### TRPP (polycystin) family

Data in the table are extracted from [234, 265] and [1103]. Broadly similar single channel conductance, mono- and di-valent cation selectivity and sensitivity to blockers are observed for TRPP2 co-expressed with TRPP1 [264]. Ca^2+^, Ba^2+^ and Sr^2+^ permeate TRPP3, but reduce inward currents carried by Na^+^. Mg^2+^ is largely impermeant and exerts a voltage dependent inhibition that increases with hyperpolarization.

#### TRPV (vanilloid) family

Activation of TRPV1 by depolarisation is strongly temperature-dependent via a channel opening rate that increases with increasing temperature. The V_1/2_ is shifted in the hyperpolarizing direction both by increasing temperature and by exogenous agonists [1253]. TRPV3 channel dysfunction caused by genetic gain-of-function mutations is implicated in the pathogenesis of skin inflammation, dermatitis, and chronic itch. In rodents, a sponateous gain-of-function matation of the TRPV3 gene causes the development of skin lesions with pruritus and dermatitis [43, 709]. In contrast to other thermoTRP channels, TRPV3 sensitizes rather than desensitizes, upon repeated stimulation with either heat or agonists [209, 716, 1347]. The sensitivity of TRPV4 to heat, but not 4*α*-PDD is lost upon patch excision. TRPV4 is activated by anandamide and arachidonic acid following P450 epoxygenase-dependent metabolism to 5,6-epoxyeicosatrienoic acid (reviewed by [887]). Activation of TRPV4 by cell swelling, but not heat, or phorbol esters, is mediated via the formation of epoxyeicosatrieonic acids. Phorbol esters bind directly to TRPV4. Different TRPV4 mutations load to a broad spectrum of dominant skeletal dysplasias [633, 1029] and spinal muscular atrophies and hereditary motor and sensory neuropathies [50, 268]. Similar mutations were also found in patients with Charcot-Marie-Tooth disease type 2C [656]. TRPV5 preferentially conducts Ca^2+^ under physiological conditions, but in the absence of extracellular Ca^2+^, conducts monovalent cations. Single channel conductances listed for TRPV5 and TRPV6 were determined in divalent cation-free extracellular solution. Ca^2+^-induced inactivation occurs at hyperpolarized potentials when Ca^2+^ is present extracellularly. Single channel events cannot be resolved (probably due to greatly reduced conductance) in the presence of extracellular divalent cations. Measurements of P_Ca_/P_Na_ for TRPV5 and TRPV6 are dependent upon ionic conditions due to anomalous mole fraction behaviour. Blockade of TRPV5 and TRPV6 by extracellular Mg^2+^ is voltage-dependent. Intracellular Mg^2+^ also exerts a voltage dependent block that is alleviated by hyperpolarization and contributes to the time-dependent activation and deactivation of TRPV6 mediated monovalent cation currents. TRPV5 and TRPV6 differ in their kinetics of Ca^2+^-dependent inactivation and recovery from inactivation. TRPV5 and TRPV6 function as homo- and hetero-tetramers.

## Voltage-gated calcium channels (Ca_V_)


$$\text{Ion channels}\to \text{Voltage-gated ion channels}\to \text{Voltage-gated calcium channels}\;({\text{Ca}}_{V})$$

### Overview:

Ca^2+^ channels are voltage-gated ion channels present in the membrane of most excitable cells. The nomenclature for Ca^2+^channels was proposed by [326] and **approved by the NC-IUPHAR Subcommittee on Ca^2+^ channels** [163]. Most Ca^2+^ channels form hetero-oligomeric complexes. The *α* 1 subunit is pore-forming and provides the binding site(s) for practically all agonists and antagonists. The 10 cloned *α*1-sub-units can be grouped into three families: (1) the high-voltage activated dihydropyridine-sensitive (L-type, Ca_V_1.x) channels; (2) the high- to moderate-voltage activated dihydropyridine-in-sensitive (Ca_V_2.x) channels and (3) the low-voltage-activated (T-type, Ca_V_3.x) channels. Each *α*1 subunit has four homologous repeats (I-IV), each repeat having six transmembrane domains (S1-S6) and a pore-forming region between S5 and S6. Voltage-dependent gating is driven by the membrane spanning S4 segment, which contains highly conserved positive charges that respond to changes in membrane potential. All of the *α*1-subunit genes give rise to alternatively spliced products. At least for high-voltage activated channels, it is likely that native channels comprise co-assemblies of *α*1, *β* and *α*2-*δ*5 subunits. The *γ* subunits have not been proven to associate with channels other than the *α*1s skeletal muscle Ca_v_1.1 channel. The *α*2-*δ*1 and *α*2-*δ*2 subunits bind gabapentin and pregabalin.

**Table:** 

Nomenclature	Ca_v_1.1	Ca_v_1.2	Ca_v_1.3	Ca_v_1.4
HGNC, UniProt	*CACNA1S*, Q13698	*CACNA1C*, Q13936	*CACNA1D*, Q01668	*CACNA1F*, O60840
Activators	FPL64176 (pEC_50_ ~7.8), (−)-(S)-BayK8644 (pEC_50_ ~7.8)	(−)-(S)-BayK8644 (pEC_50_ ~7.8), FPL64176 Concentration range: 1×10^−7^M-1×10^−6^ [566, 719]	FPL64176 (pEC_50_ ~7.8), (−)-(S)-BayK8644 (pEC_50_ ~7.8)	(−)-(S)-BayK8644 (pEC_50_ ~78)
Gating inhibitors	nifedipine (Antagonist) (pIC_50_ 6.3) [*voltage dependent* −90mV] [653] – Rat, nimodipine (Antagonist) (pIC_50_ ~6) [−70mV]	amlodipine (pIC_50_ 9.3) [496] – Rabbit, isradipine (Antagonist) (pIC_50_ 8.8) [915], nifedipine (Antagonist) (pIC_50_ 8.1–8.7) [−40mV] [958, 972] – Rat, isradipine (Antagonist) (pIC_50_ 8.5) [915], nimodipine (Antagonist) (pIC_50_ 6.8) [−80mV] [1351] – Rat	nitrendipine (Inhibition) (pIC_50_ 8.4) [1116], isradipine (dopamine neuron-like activity; splice variant-dependent) (pIC_50_ 7.8–8.2) [915], nifedipine (Antagonist) (pIC_50_ 7.7) [1116], nimodipine (Antagonist) (pIC_50_ 5.7–6.6) [−80mV ‒ −40mV] [1051, 1351] – Rat	nifedipine (Antagonist) (pIC_50_ 6) [−100mV] [802], nimodipine (Antagonist) (pIC_50_ ~6) [−70mV], nitrendipine (Antagonist) (pIC_50_ ~6) [−70mV]
Channel blockers	verapamil (Antagonist) (pIC_50_ ~5) [100mV] [1275] – Rabbit, diltiazem (Antagonist) (pIC_50_ 4.2) [−100mV] [1275] – Rabbit	verapamil (Antagonist) (pIC_50_ 5.3–6.5) [546] – Rat, diltiazem (Antagonist) (pIC_50_ 6.3) [563] – Ferret	verapamil (Antagonist) (pIC_50_ 3.7) [−70mV] [1191] – Mouse, diltiazem (pIC_50_ 3.5) [−70mV] [1191] – Mouse	diltiazem (pIC_50_ 4) [−80mV] [78] – Mouse, verapamil Concentration range: 1×10^−4^M [−80mV] [78] – Mouse
Sub/family-selective channel blockers	–	calciseptine (Antagonist) (pIC_50_ 7.1–78) [74, 248, 1307]	–	–
Functional Characteristics	L-type calcium current: High voltage-activated, very slow voltage dependent inactivation	L-type calcium current: High voltage-activated, voltage- and calcium-dependent inactivation	L-type calcium current: more negative activation voltage range than Ca_v_1.2, calcium-dependent inactivation	L-type calcium current: More negative activation voltage range than Ca_v_1.2, no/weak calcium-dependent inactivation
Comments	Serves primarily as voltage-sensor for excitation contraction coupling in skeletal muscle.	Amlodopine, isradipine, nifedipine and nimodipine are examples of dihydropyridine calcium channel antagonists. Verapamil is a phenylalkylamine calcium channel blocker. Diltiazem is an example of a benzothiazepine calcium channel blocker. Inhibition by dihydropyridines (*e.g.* nifedipine or isradipine) is voltage-dependent with a higher apparent affinity at more depolarised potentials; phenylalkylamines and diltiazem exhibit strong use-dependence with a higher apparent affinity at higher stimulation frequencies.	Ca_v_1.3 is about 5-10 fold less sensitive to dihydropyridine antagonists.	Ca_v_1.4 is less sensitive to dihydropyridine antagonists than other Ca_v_1 channels

**Table:** 

Nomenclature	Ca_v_2.1	Ca_v_2.2	Ca_v_2.3
HGNC, UniProt	*CACNA1A*, O00555	*CACNA1B*, Q00975	*CACNA1E*, Q15878
Gating inhibitors	–	NP118809 (pIC_50_ 7) [−80mV] [1391] – Rat	–
Selective gating inhibitors	*ω*-agatoxin IVA (Antagonist) (pIC_50_ 7–8.7) [−100mV – −90mV] [122, 825] – Rat, *ω*-agatoxin IVB (Antagonist) (pK_d_ 8.5) [−80mV] [10] – Rat	–	SNX482 (Antagonist) (pIC_50_ 7.5–8) [physiological voltage] [867]
Channel blockers	–	–	Ni^2+^ (Antagonist) (pIC_50_ 4.6) [−90mV] [1322]
Sub/family-selective channel blockers	*ω*-conotoxin MVIIC (Antagonist) (pIC_50_ 8.2–9.2) Concentration range: 2× 10^−6^M-5×10^−6^M [physiological voltage] [688] – Rat	*ω*-conotoxin GVIA (Antagonist) (pIC_50_ 10.4) [−80mV] [688] – Rat, *ω*-conotoxin MVIIC (Antagonist) (pIC_50_ 6.1–8.5) [−80mV] [464, 688, 797] – Rat	–
Functional Characteristics	P/Q-type calcium current: High voltage-activated, moderate voltage-dependent inactivation	N-type calcium current: High voltage-activated, moderate voltage-dependent inactivation	R-type calcium current: Moderate voltage-activated, fast voltage-dependent inactivation
Comments	All three Ca_v_2.x types directly contribute towards triggering neurotransmitter release at fast synapses in the mammalian nervous system. In many cell types, P- and Q-current components cannot be adequately separated and many researchers in the field have therefore adopted the terminology’P/Q-type’ current when referring to either component. Both of these physiologically defined current types are conducted by alternative forms of Ca_v_2.1. Ziconotide (a synthetic peptide equivalent to *ω*-conotoxin MVIIA) has been approved for the treatment of chronic pain [1321].	–	–

**Table:** 

Nomenclature	Ca_v_3.1	Ca_v_3.2	Ca_v_3.3
HGNC, UniProt	*CACNA1G*, O43497	*CACNA1H*, O95180	*CACNA1I*, Q9P0X4
Gating inhibitors	kurtoxin (Antagonist) (pIC_50_ 73–7.8) [−90mV] [205, 1106] – Rat	kurtoxin (Antagonist) (pIC_50_ 73–7.6) [−90mV] [205, 1106] – Rat	–
Channel blockers	Z944 (Pore blocker) (pIC_50_ 7.3) [−80mV] [1227], TTA-A2 (Pore blocker) (pIC_50_ 7) [−75mV] [360], mibefradil (Antagonist) (pic_50_ 6–6.6) [−110mV – −100mV] [783], Ni^2+^ (Antagonist) (pIC_50_ 3.6–3.8) [*voltage dependent* −90mV] [669] – Rat	TTA-A2 (Pore blocker) (pIC_50_ 8) [−75mV] [360], mibefradil (Pore blocker) (pIC_50_ 5.9–7.2) [−110mV – −80mV] [783], Z944 (Pore blocker) (pIC_50_ 6.8) [−75mV] [1227], and derivatives pimozide (Pore blocker) (pIC_5_g 6.8) [575], efonidipine (Pore blocker) (pIC_50_ 6.4) [674], Ni^2+^ (Pore blocker) (pIC_50_ 4.9–5.2) [*voltage dependent* −90mV] [669]	TTA-A2 (Pore blocker) (pIC_50_ 7.5) [−75mV] [360], Z944 (Pore blocker) (pIC_50_ 7) [−75mV] [1227], mibefradil (Antagonist) (pIC_50_ 5.8) [−110mV] [783], Ni^2+^ (Antagonist) (pIC_50_ 3.7–4.1) [*voltage dependent* −90mV] [669] – Rat
Functional Characteristics	T-type calcium current: Low voltage-activated, fast voltage-dependent inactivation	T-type calcium current: Low voltage-activated, fast voltage-dependent inactivation	T-type calcium current: Low voltage-activated, moderate voltage-dependent inactivation

## Voltage-gated proton channel (H_V_1)


$$\text{Ion channels}\to \text{Voltage-gated ion channels}\to \text{Voltage-gated proton channels}\;(H_{V}1)$$

### Overview:

The voltage-gated proton channel (provisionally denoted H_v_1) is a putative 4TM proton-selective channel gated by membrane depolarization and which is sensitive to the transmembrane pH gradient [155, 251, 252, 1013, 1065]. The structure of H_v_1 is homologous to the voltage sensing domain (VSD) of the superfamily of voltage-gated ion channels (*i.e.* segments S1 to S4) and contains no discernable pore region [1013, 1065]. Proton flux through Hv1 is instead most likely mediated by a hydrogen-bonded chain [253, 855] formed in a crevice of the protein when the voltage-sensing S4 helix moves in response to a change in transmembrane potential [1012, 1329]. Proton selective conduction requires an aspartate residue at the center of the pore [176, 850, 1127]. Both selectivity and conduction may result from obligatory protonation by each conducted proton [254, 304]. H_v_1 expresses largely as a dimer mediated by intracellular C-terminal coiled-coil interactions [694] but individual promoters nonetheless support gated H^+^ flux *via* separate conduction pathways [619, 673, 964, 1214]. Within dimeric structures, the two protomers do not function independently, but display co-operative interactions during gating resulting in increased voltage sensitivity, but slower activation, of the dimeric, *versus* monomeric, complexes [393, 1215]. The otopetrin proteins appear to form proton-selective ion channels and to date 3 subtypes have been identified in eukaryotes; otopetrin 1 [1011, 1230], otopetrin 2 [732] and otopetrin 3 [498].

**Table:** 

Nomenclature	H_v_1
HGNC, UniProt	*HVCN1*, Q96D96
Channel blockers	Zn^2+^ (pIC_50_ ~5.7–6.3), Cd^2+^ (pIC_50_ ~5)
Functional Characteristics	Activated by membrane depolarization mediating macroscopic currents with time-, voltage- and pH-dependence; outwardly rectifying; voltage dependent kinetics with relatively slow current activation sensitive to extracellular pH and temperature, relatively fast deactivation; voltage threshold for current activation determined by pH gradient (ΔpH = pH_o_-pH_i_) across the membrane

### Comments:

The voltage threshold (V_thr_) for activation of Hv1 is not fixed but is set by the pH gradient across the membrane such that V_thr_ is positive to the Nernst potential for H^+^, which ensures that only outwardly directed flux of H^+^ occurs under physiological conditions [155, 199, 251, 252]. Phosphorylation of H_v_1 within the N-terminal domain by PKC enhances the gating of the channel [255, 256, 848]. Tabulated IC_50_ values for Zn^2+^ and Cd^2+^ are for heterologously expressed human and mouse H_v_1 [1013, 1065]. Zn^2+^ is not a conventional pore blocker, but is coordinated by two, or more, external protonation sites involving histamine residues [1013]. Zn^2+^ binding may occur at the dimer interface between pairs of histamine residues from both monomers where it may interfere with channel opening [849]. Mouse knockout studies [319, 836, 1014] support the view that H_v_1 participates in both charge compensation and pH regulation in granulocytes during the respiratory burst of NA-DPH oxidase-dependent reactive oxygen species production that assists in the clearance of bacterial pathogens [257, 450, 569, 1081]. Additional physiological functions of H_v_1 are reviewed by [155].

## Voltage-gated sodium channels (Na_V_)


$$\text{Ion channels}\to \text{Voltage-gated ion channels}\to \text{Voltage-gated sodium channels}\;({\text{Na}}_{V})$$

### Overview:

Sodium channels are voltage-gated sodium-selective ion channels present in the membrane of most excitable cells. Sodium channels comprise of one pore-forming *α* subunit, which may be associated with either one or two *β* subunits [516]. *α*-Subunits consist of four homologous domains (I-IV), each containing six transmembrane segments (S1-S6) and a pore-forming loop. The positively charged fourth transmembrane segment (S4) acts as a voltage sensor and is involved in channel gating. The crystal structure of the bacterial NavAb channel has revealed a number of novel structural features compared to earlier potassium channel structures including a short selectivity filter with ion selectivity determined by interactions with glutamate side chains [944]. Interestingly, the pore region is penetrated by fatty acyl chains that extend into the central cavity which may allow the entry of small, hydrophobic pore-blocking drugs [944]. Auxiliary *β*1, *β*2, *β*3 and *β*4 subunits consist of a large extracellular N-terminal domain, a single transmembrane segment and a shorter cytoplasmic domain.

The nomenclature for sodium channels was proposed by Goldin *et al*., (2000) [390] and approved by the NC-IUPHAR Subcommittee on sodium channels (Catterall *et al*., 2005, [161]).

**Table:** 

Nomenclature	Na_v_1.1	Na_v_1.2	Na_v_1.3	Na_v_1.4
HGNC, UniProt	*SCN1A*, P35498	*SCN2A*, Q99250	*SCN3A*, Q9NY46	*SCN4A*, P35499
Sub/family-selective activators	batrachotoxin, veratridine	batrachotoxin (Agonist) (pK_d_ 9.1) [physiological voltage] [710] – Rat, veratridine (Partial agonist) (pK_d_ 5.2) [physiological voltage] [162] – Rat	batrachotoxin, veratridine	batrachotoxin (Full agonist) Concentration range: 5×10^−6^M [−100mV] [1291] – Rat, veratridine (Partial agonist) Concentration range: 2×10^−4^M [−100mV] [1291] – Rat
Channel blockers	tetrodotoxin (Pore blocker) (pK_d_ 8) [−100mV] [1126]-Rat	–	–	–
Sub/family-selective channel blockers	Hm1a [918] – Rat, saxitoxin (Pore blocker)	saxitoxin (Pore blocker) (pIC_50_ 8.8) [−120mV] [128] – Rat, tetrodotoxin (Pore blocker) (pIC_50_ 8) [−120mV] [128] – Rat, lacosamide (Antagonist) (pIC_50_ 4.5) [−80mV] [2] – Rat	tetrodotoxin (Pore blocker) (pIC_50_ 8.4) [194], saxitoxin (Pore blocker)	saxitoxin (Pore blocker) (pIC_50_ 8.4) [−100mV] [957] – Rat, tetrodotoxin (Pore blocker) (pIC_50_ 7.6) [−120mV] [167], *μ*-conotoxin GII-IA (Pore blocker) (pIC_50_ 5.9) [−100mV] [167]
Functional Characteristics	Activation V_0.5_ = −20 mV. Fast inactivation (*τ* = 0.7 ms for peak sodium current).	Activation V_0.5_ = −24 mV. Fast inactivation (*τ* = 0.8 ms for peak sodium current).	Activation V_0.5_ = −24 mV. Fast inactivation (0.8 ms)	Activation V_0 5_ = −30 mV. Fast inactivation (0.6 ms)

**Table:** 

Nomenclature	Na_v_1.5	Na_v_1.6	Na_v_1.7	Na_v_1.8	Na_v_1.9
HGNC, UniProt	*SCN5A*, Q14524	*SCN8A*, Q9UQD0	*SCN9A*, Q15858	*SCN10A*, Q9Y5Y9	*SCN11A*, Q9UI33
Sub/family-selective activators	batrachotoxin (Full agonist) (pK_d_ 7.6) [physiological voltage] [1096] – Rat, veratridine (Partial agonist) (pIC_50_ 6.3) [−30mV] [1279] – Rat	batrachotoxin, veratridine	batrachotoxin, veratridine	–	–
Sub/family-selective channel blockers	tetrodotoxin (Pore blocker) (pK_d_ 5.8) [−80mV] [224, 1394]-Rat	tetrodotoxin (Pore blocker) (pIC_50_ 9) [−130mV] [277] — Rat, saxitoxin (Pore blocker)	tetrodotoxin (Pore blocker) (pIC_50_ 7.6) [−100mV] [612], saxitoxin (Pore blocker) (pIC_50_ 6.2) [1272]	tetrodotoxin (Pore blocker) (pIC_50_ 4.2) [−60mV] [17]-Rat	tetrodotoxin (Pore block-er) (pIC_50_ 4.4) [−120mV] [228] – Rat
Selective channel blockers	–	–	–	PF-01247324 (Pore blocker) (pIC_50_ 6.7) [*voltage dependent]* [945]	–
Functional Characteristics	Activation V_0.5_ = −26 mV. Fast inactivation (*τ* = 1 ms for peak sodium current).	Activation V_0.5_ = −29 mV. Fast inactivation (1 ms)	Activation V_0.5_ = −27 mV. Fast inactivation (0.5 ms)	Activation V_0.5_ = −16 mV. Inactivation (6 ms)	Activation V_0.5_ = −32 mV. Slow inactivation (16 ms)

### Comments:

Sodium channels are also blocked by local anaesthetic agents, antiarrythmic drugs and antiepileptic drugs. In general, these drugs are not highly selective among channel subtypes. There are two clear functional fingerprints for distinguishing different subtypes. These are sensitivity to tetrodotoxin (Na_V_1.5, Na_V_1.8 and Na_V_1.9 are much less sensitive to block) and rate of fast inactivation (Na_V_1.8 and particularly Na_V_1.9 inactivate more slowly). All sodium channels also have a slow inactivation process that is engaged during long depolarizations (>100 ms) or repetitive trains of stimuli. All sodium channel subtypes are blocked by intracellular QX-314.

### Comments on Voltage-gated ion channels:

The voltage-dependent anion channels (VDACs) plays a key role in regulating metabolic and energetic flux across the outer mitochondrial membrane. It is involved in the transport of ATP, ADP, pyruvate, malate, and other metabolites, and thus communicates extensively with enzymes from metabolic pathways. They are a class of porin ion channel located on the outer mitochondrial membrane. VDAC1, VDAC2 and VDAC3 are involved in the regulation of apoptosis, cell metabolism, mitochondrial apoptosis, and spermatogenesis [759, 1105].

The calcium homeostasis modulator (CALHM) ion channels are apparently voltage- and extracellular Ca^2+^-gated, and constitute a novel ion channel family that is widely expressed in the brain and taste buds throughout vertebrates and in sensory neurons and body wall muscles in *C. elegans*. In humans, the CALHM family encompasses six paralogs, some of which function as non-selective channels that are permeable to large substances such as ATP. CALHM channels are thought to play important roles in neuronal excitability, neurotransmission of tastes, and muscle cell function. The voltage- and extracellular Ca^2+^-dependent gating mechanisms, structural features that define the gate and ion permeation pathway and additional physiological roles, remain to be discovered [752].

## Other ion channels


Ion channels→Other ion channels

### Overview:

A number of ion channels in the human genome do not fit readily into the classification of either ligand-gated or voltage-gated ion channels. These are identified below.

## Aquaporins


Ion channels→Other ion channels→Aquaporins

### Overview:

Aquaporins and aquaglyceroporins are membrane channels that allow the permeation of water and certain other small solutes across the cell membrane, or in the case of AQP6, AQP11 and AQP12A, intracellular membranes, such as vesicles and the endoplasmic reticulum membrane [638]. Since the isolation and cloning of the first aquaporin (AQP1) [989], 12 additional mammalian members of the family have been identified, although little is known about the functional properties of one of these (*AQP12A;* Q8IXF9) and it is thus not tabulated. The other 12 aquaporins can be broadly divided into three families: orthodox aquaporins (AQP0,−1,−2,−4,−5, −6 and −8) permeable mainly to water, but for some additional solutes [243]; aquaglyceroporins (AQP3,−7–9 and−10), additionally permeable to glycerol and for some isoforms urea [604], and superaquaporins (AQP11 and 12) located within cells [513]. Some aquaporins also conduct ammonia and/or H_2_O_2_ giving rise to the terms’ammoniaporins’ (’aquaammoniaporins’) and ’peroxiporins’, respectively. Aquaporins are impermeable to protons and other inorganic and organic cations, with the possible exception of AQP1, although this is controversial [604]. One or more members of this family of proteins have been found to be expressed in almost all tissues of the body [reviewed in Yang (2017) [1357]]. AQPs are involved in numerous processes that include systemic water homeostasis, adipocyte metabolism, brain oedema, cell migration and fluid secretion by epithelia. Loss of function mutations of some human AQPs, or their disruption by autoantibodies further underscore their importance [reviewed by Verkman *et al*. (2014) [1245], Kitchen *et al*. (2105) [604]].

Functional AQPs exist as homotetramers that are the water conducting units wherein individual AQP subunits (each a protomer) have six TM helices and two half helices that constitute a seventh’pseudotransmembrane domain’ that surrounds a narrow water conducting channel [638]. In addition to the four pores contributed by the protomers, an additional hydrophobic pore exists within the center of the complex [638] that may mediate the transport through AQP1. Although numerous small molecule inhibitors of aquaporins, particularly APQ1, have been reported primarily from *Xenopus* oocyte swelling assays, the activity of most has subsequently been disputed upon retesting using assays of water transport that are less prone to various artifacts [331] and they are therefore excluded from the tables [see Tradtrantip *et al*. (2017) [1221] for a review].

**Table:** 

Nomenclature	AQPO	AQP1	AQP2	AQP3
HGNC, UniProt	*MIP*, P30301	*AQP1*, P29972	*AQP2*, P41181	*AQP3*, Q92482
Endogenous activator	AQP0 is gated by calmodulin [604]	cGMP (see comment)	–	–
Permeability	water (rat single channel permeability 0.25 x 10^−14^cm^3^s”^1^) (Rat) [1359]	water (rat single channel permeability 6.0 x 10^−14^cm^3^s^−1^), ammonia, H_2_O_2_ [383, 1359]	water (rat single channel permeability 3.3 x 10^−14^cm^3^s^−1^) [751]	water (rat single channel permeability 2.1 x 10^−14^cm^3^s^1^), glycerol, ammonia, H_2_O_2_ [102, 383, 1359]
Inhibitors	Hg^2+^	Ag^+^, Hg^2+^, pCMBS	Hg^2+^	Auphen (pIC_50_ 6.1) [785], Audien (pIC_50_ 4.8) [785], Hg^2+^
Channel blockers	–	5-Hydroxymethyl-2-furfural (Inhibition) (pIC_50_ 6.4) [202]	–	–
Comments	–	Permeability to H_2_O_2_ has been demonstrated for rat, but not human, AQP1 [102]. Numerous small molecule inhibitors of AQP1 have been proposed, but re-evaluation indicates that they have no significant effect upon water permeability at concentrations in excess of their originally reported IC_50_ values [377]. A fifth pore located at the central axis of the tetrameric complex has, controversially, been described as a cation conductance activated by cGMP and phosphorylation by protein kinases A and C. Evidence in support and against this proposal is discussed in detail by Kitchen *et al*. (2015) [604].	–	AQP3 is also inhibited by acid pH: permeability to urea is controversial [604].

**Table:** 

Nomenclature	AQP4	AQP5
HGNC, UniProt	*AQP4*, P55087	*AQP5*, P55064
Permeability	water (rat single channel permeability 24 x 10^−14^cm^3^s^−1^ [1359]	water (rat single channel permeability 5.0 x 10^−14^cm^3^s^−1^), H_2_O_2_ [561]
Inhibitors	–	Hg^2+^
Comments	AQP4 is inhibited by PKC activation (although this is probably due to phosphorylation-dependent protein localisation rather than inhibition of the channel *per se*), but not by HgCl_2_. AQP4 is predicted to be permeable to NO [1299]. Recent work suggests that the membrane trafficking of AQP4 could be an alternative target to pore-blockers [605].	AQP5 may conduct CO_2_ [383].

**Table:** 

Nomenclature	AQP6	AQP7	AQP8	AQP9	AQP10
HGNC, UniProt	*AQP6*, Q13520	*AQP7*, O14520	*AQP8*, O94778	*AQP9*, O43315	*AQP10*, Q96PS8
Permeability	water (zero, or very low basal, permeability is enhanced by low pH and in mouse and rat by Hg^2+^), glycerol, ammonia, urea, anions [383, 477, 604, 1007]	water (high), glycerol, ammonia, urea [383, 511]	water (mouse single channel permeability 8.2 x 10^−14^cm^3^s^−1^), ammonia, H_2_O_2_ [102, 383, 604, 751]	water (low), glycerol, ammonia, urea, H_2_O_2_, monocarboxylates [383, 476, 1007, 1306]	water (low), glycerol, urea [512]
Activators	Hg^2+^	–	–	–	–
Inhibitors	–	Auphen (Effective at 15 *μ*M), Hg^2+^	Hg^2+^	Hg^2+^, phloretin	Hg^2+^
Comments	AQP6 is an intracellular channel that localises to acid secreting intercalated cells of the renal collecting ducts. Notably, AQP6 is activated by Hg^2+^ and by low pH and is unusually permeable to anions (with the permeability sequence NO_3_-> I^−^≫Br^−^>Cl^−^≫Fl^−^) as well as water, both through the monomeric pore [604, 1007].	AQP7 also transports silicon [377],	Permeability to urea is controversial, but might be explained by differences between mouse and human caused by a pore-lining amino acid residue that differs between species [604].	AQP9 may transport silicon [377].	It is not known if AQP10 is permeable to ammonia. Permeability to silicon has been described [377].

## Chloride channels


Ion channels→Other ion channels→Chloride channels

### Overview:

Chloride channels are a functionally and structurally diverse group of anion selective channels involved in processes including the regulation of the excitability of neurones, skeletal, cardiac and smooth muscle, cell volume regulation, transepithelial salt transport, the acidification of internal and extracellular compartments, the cell cycle and apoptosis (reviewed in [309]). Excluding the transmittergated GABA_A_ and glycine receptors (see separate tables), well characterised chloride channels can be classified as certain members of the voltage-sensitive ClC subfamily, calcium-activated channels, high (maxi) conductance channels, the cystic fibrosis transmembrane conductance regulator (CFTR) and volume regulated channels [1246]. No official recommendation exists regarding the classification of chloride channels. Functional chloride channels that have been cloned from, or characterised within, mammalian tissues are listed with the exception of several classes of intracellular channels (*e.g.* CLIC) that are reviewed by in [317].

## ClC family


Ion channels→Other ion channels→Chloride channels→CIC family

### Overview:

The mammalian ClC family (reviewed in [8, 186, 309, 312, 535]) contains 9 members that fall, on the basis of sequence homology, into three groups; ClC-1, ClC-2, hClC-Ka (rClC-K1) and hClC-Kb (rClC-K2); ClC-3 to ClC-5, and ClC-6 and −7. ClC-1 and ClC-2 are plasma membrane chloride channels. ClC-Ka and ClC-Kb are also plasma membrane channels (largely expressed in the kidney and inner ear) when associated with barttin (*BSND*, Q8WZ55), a 320 amino acid 2TM pro-tein [332]. The localisation of the remaining members of the ClC family is likely to be predominantly intracellular *in vivo*, although they may traffic to the plasma membrane in overexpression systems. Numerous recent reports indicate that ClC-4, ClC-5, ClC-6 and ClC-7 (and by inference ClC-3) function as Cl^−^/H^+^ antiporters (secondary active transport), rather than classical Cl^−^ channels [399, 679, 865, 969, 1072]; reviewed in [8, 994]). It has recently been reported that the activity of ClC-5 as a Cl^−^/H^+^ exchanger is important for renal endocytosis [895]. Alternative splicing increases the structural diversity within the ClC family. The crystal structure of two bacterial ClC proteins has been described [313] and a eukaryotic ClC transporter (Cm-CLC) has recently been described at 3.5 Å resolution [346]. Each ClC subunit, with a complex topology of 18 intramembrane segments, contributes a single pore to a dimeric ‘double-barrelled’ ClC channel that contains two independently gated pores, confirming the predictions of previous functional and structural investigations (reviewed in [186, 312, 535, 994]). As found for ClC-4, ClC-5, ClC-6 and ClC-7, the prokaryotic ClC homologue (ClC-ec1) and CmCLC function as H^+^/Cl antiporters, rather than as ion channels [7, 346]. The generation of monomers from dimeric ClC-ec1 has firmly established that each ClC subunit is a functional unit for transport and that cross-subunit interaction is not required for Cl^−^/H^+^ exchange in ClC transporters [1026].

**Table:** 

Nomenclature	ClC-1	ClC-2
HGNC, UniProt	*CLCN1*, P35523	*CLCN2*, P51788
Endogenous activators	–	arachidonic acid
Activators	–	lubiprostone, omeprazole
Channel blockers	9-anthroic acid, S-(−)CPB, S-(−)CPP, Cd2+, Zn^2+^, fenofibric acid, niflumic acid	GaTx2 (pK_d_ 10.8) [*voltage dependent* −100mV], Cd^2+^, NPPB, Zn^2+^, diphenylamine-2-carboxylic acid
Functional Characteristics	*γ* = 1-1.5 pS; voltage-activated (depolarization) (by fast gating of single protopores and a slower common gate allowing both pores to open simultaneously); inwardly rectifying; incomplete deactivation upon repolarization, ATP binding to cytoplasmic cystathionine *β*-synthase related (CBS) domains inhibits ClC-1 (by closure of the common gate), depending on its redox status	*γ* = 2-3 pS; voltage-activated by membrane hyperpolarization by fast protopore and slow cooperative gating; channels only open negative to E_Cl_ resulting in steady-state inward rectification; voltage dependence modulated by permeant anions; activated by cell swelling, PKA, and weak extracellular acidosis; potentiated by SGK1; inhibited by phosphorylation by p34(cdc2)/cyclin B; cell surface expression and activity increased by association with Hsp90
Comments	ClC-1 is constitutively active	ClC-2 is also activated by amidation

**Table:** 

Nomenclature	ClC-Ka	ClC-Kb	ClC-3	ClC-4
HGNC, UniProt	*CLCNKA*, P51800	*CLCNKB*, P51801	*CLCN3*, P51790	*CLCN4*, P51793
Activators	niflumic acid (pEC_50_ 3–5)	niflumic acid (pEC_50_ 3–5)	–	–
Channel blockers	3-phenyl-CPP, DIDS, niflumic acid	3-phenyl-CPP, DIDS	phloretin (pIC_50_ 4.5)	Zn^2+^ (pIC_50_ 4.3) [919], Cd^2+^ (pIC_50_ 4.2) [919]
Functional Characteristics	*γ* = 26 pS; linear current-voltage relationship except at very negative potentials; no time dependence; inhibited by extracellular protons (p*K* = 7.1); potentiated by extracellular Ca^2+^	Bidirectional rectification; no time dependence; inhibited by extracellular protons; potentiated by extracellular Ca^2+^	Cl^−^/H^+^ antiporter [792]; pronounced outward rectification; slow activation, fast deactivation; activity enhanced by CaM kinase II; inhibited by intracellular Ins(3,4,5,6)P4 and extracellular acidosis	Cl^−^/H^+^ antiporter (2Chl^−^:H^+^) [22, 969, 1072]; extreme outward rectification; voltage-dependent gating with midpoint of activation at +73 mV [913]; rapid activation and deactivation; inhibited by extracellular acidosis; non-hydrolytic nucleotide binding required for full activity
Comments	ClC-Ka is constitutively active (when co-expressed with barttin), and can be blocked by benzofuran derivatives	ClC-Kb is constitutively active (when co-expressed with barttin), and can be blocked by benzofuran derivatives	insensitive to the channel blockers DIDS, NPPB and tamoxifen (10 *μ*M)	–

**Table:** 

Nomenclature	ClC-5	ClC-6	ClC-7
HGNC, UniProt	*CLCN5*, P51795	*CLCN6*, P51797	*CLCN7*, P51798
Channel blockers	–	DIDS (pIC_50_ 3)	DIDS (pIC_50_ 4.4) [1085], NS5818 (pIC_50_ 4.3) [1085], NPPB (pIC_50_ 3.8) [1085]
Functional Characteristics	Cl^−^/H^+^ anti porter (2Cl^−^:1H^+^) [969, 1072, 1121, 1428]; extreme outward rectification; voltage-dependent gating with midpoint of activation of 116.0 mV; rapid activation and deactivation; potentiated and inhibited by intracellular and extracellular acidosis, respectively; ATP binding to cytoplasmic cystathionine *β*-synthase related (CBS) domains activates ClC-5	Cl^−^/H^+^ antiporter (2Cl^−^:1H^+^) [865]; outward rectification, rapid activation and deactivation	Cl^−^/H^+^ anti porter (2Cl^−^:1H^+^) [399, 679, 1085]; strong outward rectification; voltage-dependent gating with a threshold more positive than ~ + 20 mV; very slow activation and deactivation
Comments	Insensitive to the channel blockers DIDS (1 mM), diphenylamine-2-carboxylic acid (1 mM), 9-anthroic acid (2 mM), NPPB (0.5 mM) and niflumic acid (1 mM)	–	active when co-expressed with Ostm1

### Comments:

ClC channels display the permeability sequence Cl^−^ > Br^−^ > I^−^ (at physiological pH). ClC-1 has significant opening probability at resting membrane potential, accounting for 75% of the membrane conductance at rest in skeletal muscle, and is important for stabilization of the membrane potential. S-(−)CPP, 9-anthroic acid and niflumic acid act intracellularly and exhibit a strongly voltage-dependent block with strong inhibition at negative voltages and relief of block at depolarized potentials ([698] and reviewed in [993]). Inhibition of ClC-2 by the peptide GaTx2, from *Leiurus quinquestriatus herbareus* venom, is likely to occur through inhibition of channel gating, rather than direct open channel blockade [1204]. Although ClC-2 can be activated by cell swelling, it does not correspond to the VRAC channel (see below). Alternative potential physiological functions for ClC-2 are reviewed in [980]. Functional expression of human ClC-Ka and ClC-Kb requires the presence of barttin [332, 1079] reviewed in [337]. The properties of ClC-Ka/barttin and ClC-Kb/barttin tabulated are those observed in mammalian expression systems: in oocytes the channels display time- and voltage-dependent gating. The rodent homologue (ClC-K1) of ClC-Ka demonstrates limited expression as a homomer, but its function is enhanced by barttin which increases both channel opening probablility in the physiological range of potentials [332, 352, 1079] reviewed in [337]). ClC-Ka is approximately 5 to 6-fold more sensitive to block by 3-phenyl-CPP and DIDS than ClC-Kb, while newly synthesized benzofuran derivatives showed the same blocking affinity (<10 *μ*M) on both CLC-K isoforms [699]. The biophysical and pharmacological properties of ClC-3, and the relationship of the protein to the endogenous volume-regulated anion channel(s) VRAC [23, 412] are controversial and further complicated by the possibility that ClC-3 may function as both a Cl^−^/H^+^ exchanger and an ion channel [23, 969, 1295]. The functional properties tabulated are those most consistent with the close structural relationship between ClC-3, ClC-4 and ClC-5. Activation of heterologously expressed ClC-3 by cell swelling in response to hypotonic solutions is disputed, as are many other aspects of its regulation. Dependent upon the predominant extracellular anion (*e.g.* SCN^−^ versus Cl^−^), ClC-4 can operate in two transport modes: a slippage mode in which behaves as an ion channel and an exchanger mode in which unitary transport rate is 10-fold lower [22]. Similar findings have been made for ClC-5 [1392]. ClC-7 associates with a *β* subunit, Ostm1, which increases the stability of the former [658] and is essential for its function [679].

## CFTR


Ion channels→Other ion channels→Chloride channels→CFTR

### Overview:

CFTR, a 12TM, ABC transporter-type protein, is a cAMP-regulated epithelial cell membrane Cl^−^ channel involved in normal fluid transport across various epithelia. Of the 1700 mutations identified in CFTR, the most common is the deletion mutant ΔF508 (a class 2 mutation) which results in impaired trafficking of CFTR and reduces its incorporation into the plasma membrane causing cystic fibrosis (reviewed in [230]). Channels carrying the ΔF508 mutation that do traffic to the plasma membrane demonstrate gating defects. Thus, pharmacological restoration of the function of the ΔF508 mutant would require a compound that embodies’corrector’ (*i.e.* facilitates folding andtrafficking to the cell surface) and ’potentiator’ (*i.e.* promotes opening of channels at the cell surface) activities [230]. In addition to acting as an anion channel *per se*, CFTR may act as a regulator of several other conductances including inhibition of the epithelial Na channel (ENaC), calcium activated chloride channels (CaCC) and volume regulated anion channel (VRAC), activation of the outwardly rectifying chloride channel (ORCC), and enhancement of the sulphonylurea sensitivity of the renal outer medullary potassium channel (ROMK2), (reviewed in [878]). CFTR also regulates TRPV4, which provides the Ca^2+^ signal for regulatory volume decrease in airway epithelia [42]. The activities of CFTR and the chloride-bicarbonate exchangers SLC26A3 (DRA) and SLC26A6 (PAT1) are mutually enhanced by a physical association between the regulatory (R) domain of CFTR and the STAS domain of the SCL26 transporters, an effect facilitated by PKA-mediated phosphorylation of the R domain of CFTR [616].

**Table:** 

Nomenclature	CFTR
HGNC, UniProt	*CFTR*, P13569
Activators	felodipine (Potentiation) (pK_I_ 8.4) [949], CBIQ (Potentiation), NS004 (Potentiation), UCCF-029(Potentiation), UCCF-339 (Potentiation), UCCF-853 (Potentiation), apigenin (Potentiation), capsaicin (Potentiation), genistein (Potentiation), ivacaftor (Potentiation), nimodipine(Potentiation), phenylglycine-01 (Potentiation), sulfonamide-01 (Potentiation)
Selective inhibitors	crofelemer (pIC_50_ 5.2) [1222]
Channel blockers	glibenclamide (pK_i_ 4.7) [1100], intracellular CFTR_inh_-172 (intracellular application prolongs mean closed time), GaTx1, extracellular GlyH-101
Functional Characteristics	*γ* = 6-10 pS; permeability sequence = Br ≥ Cl^−^ > I^−^ > F^−^, (P_I_/P_Cl_ = 0.1-0.85); slight outward rectification; phosphorylation necessary for activation by ATP binding at binding nucleotide binding domains (NBD)1 and 2; positively regulated by PKC and PKGII (tissue specific); regulated by several interacting proteins including syntaxin 1A, Munc18 and PDZ domain proteins such as NHERF (EBP50) and CAP70
Comments	UCCF-339, UCCF-029, apigenin and genistein are examples of flavones. UCCF-853 and NS004 are examples of benzimidazolones. CBIQ is an example of a benzoquinoline. Felodipine and nimodipine are examples of 1,4-dihydropyridines. Phenylglycine-01 is an example of a phenylglycine. Sulfonamide-01 is an example of a sulfonamide. Malonic acid hydrazide conjugates are also CFTR channel blockers (see Verkman and Galietta, 2009 [1246])

### Comments:

In addition to the agents listed in the table, the novel small molecule, ataluren, induces translational read through of nonsense mutations in CFTR (reviewed in [1118]). Corrector compounds that aid the folding of DF508CFTR to increase the amount of protein expressed and potentially delivered to the cell surface include VX-532 (which is also a potentiator), VRT-325, KM11060, Corr-3a and Corr-4a see [1246] for details and structures of Corr-3a and Corr-4a). Inhibition of CFTR by intracellular application of the peptide GaTx, from *Leiurus quinquestriatus herbareus* venom, occurs preferentially for the closed state of the channel [366]. CFTR contains two cytoplasmic nucleotide binding domains (NBDs) that bind ATP. A single open-closing cycle is hypothesised to involve, in sequence: binding of ATP at the N-terminal NBD1, ATP binding to the C-terminal NBD2 leading to the formation of an intramolecular NBD1-NBD2 dimer associated with the open state, and subsequent ATP hydrolysis at NBD2 facilitating dissociation of the dimer and channel closing, and the initiation of a new gating cycle [24, 842]. Phosphorylation by PKA at sites within a cytoplasmic regulatory (R) domain facilitates the interaction of the two NBD domains. PKC (and PKGII within intestinal epithelial cells via guanylin-stimulated cyclic GMP formation) positively regulate CFTR activity.

## Calcium activated chloride channel (CaCC)


Ion channels→Other ion channels→Chloride channels→Calcium activated chloride channel (CaCC)

### Overview:

Chloride channels activated by intracellular calcium (CaCC) are widely expressed in excitable and non-excitable cells where they perform diverse functions [439]. The molecular nature of CaCC has been uncertain with both *CLCA, TWEETY* and *BEST* genes having been considered as likely candidates [309, 440, 729]. It is now accepted that CLCA expression products are unlikely to form channels *per se* and probably function as cell adhesion proteins, or are secreted [939]. Similarly, *TWEETY* gene products do not recapictulate the properties of endogenous CaCC. The bestrophins encoded by genes *BEST1-4* have a topology more consistent with ion channels [440] and form chloride channels that are activated by physiological concentrations of Ca^2+^, but whether such activation is direct is not known [440]. However, currents generated by bestrophin over-expression do not resemble native CaCC currents. The evidence for and against bestrophin proteins forming CaCC is critically reviewed by Duran *et al*. [309]. Recently, a new gene family, TMEM16 (anoctamin) consisting of 10 members (TMEM16A-K; anoctamin 1-10) has been identified and there is firm evidence that some of these members form chloride channels [308, 649]. TMEM16A (anoctamin 1; Ano 1) produces Ca^2+^-activated Cl^−^ currents with kinetics similar to native CaCC currents recorded from different cell types [157, 1028, 1082, 1364]. Knockdown of TMEM16A greatly reduces currents mediated by calcium-activated chloride channels in submandibular gland cells [1364] and smooth muscle cells from pulmonary artery [777]. In TMEM16A^−/−^ mice secretion of Ca^2+^-dependent Cl^−^secretion by several epithelia is reduced [920, 1028]. Alternative splicing regulates the voltage- and Ca^2+^- dependence of TMEM16A and such processing may be tissue-specific manner and thus contribute to functional diversity [349]. There are also reports that TMEM16B (anoctamin 2; Ano 2) supports CaCC activity (*e.g.*[971]) and in TMEM16B^−/−^ mice Ca-activated Cl^−^ currents in the main olfactory epithelium (MOE) and in the vomeronasal organ are virtually absent [103].

**Table:** 

Nomenclature	CaCC
HGNC, UniProt	*ANO1*, Q5XXA6
Endogenous activators	intracellular Ca^2+^
Inhibitors	MONNA (pIC_50_ 7.1) [1228]
Selective inhibitors	Ani9 (pIC_50_ 7) [1090], crofelemer (pIC_50_ 5.2) [1222]
Endogenous channel blockers	Ins(3,4,5,6)P_4_
Channel blockers	9-anthroic acid, CaCCinh-A01 [246], DCDPC, DIDS, NPPB, SITS, flufenamic acid, fluoxetine, mibefradil, niflumic acid, tannic acid
Functional Characteristics	*γ* = 0.5-5 pS; permeability sequence, SCN^−^ > NO_3_^->^ I^−^ > Br^->^ Cl^−^ > F^−^; relative permeability of SCN^−^:Cl^−^ ~8. I-:Cl^−^ ~3, aspartate: Cl^−^ −0.15, outward rectification (decreased by increasing [Ca^2+^]_i_); sensitivity to activation by [Ca^2+^]_i_ decreased at hyperpolarized potentials; slow activation at positive potentials (accelerated by increasing [Ca^2+^]_i_); rapid deactivation at negative potentials, deactivation kinetics modulated by anions binding to an external site; modulated by redox status
Comments	A CaCC (TMEM16A) potentiator compound (ETD002, undisclosed structure; acquired by Roche from Enterprise Therapeutics) has entered Phase 1 clinical evaluation as a novel approach that has potential to provide benefit to all patients with cystic fibrosis (mentioned in [975]). Up-regulating chloride transport *via* CaCC is proposed to mitigate the effect of loss of chloride transport *via* CFTR in CF. See Enterprise Therapeutics’ reports of CaCC potentiator ETX001 for more detailed background information [235, 236].

### Comments:

Blockade of I^Cl(Ca)^ by niflumic acid, DIDS and 9-anthroic acid is voltage-dependent whereas block by NPPB is voltage-independent [439]. Extracellular niflumic acid; DCDPC and 9-anthroic acid (but not DIDS) exert a complex effect upon I^Cl(Ca)^ in vascular smooth muscle, enhancing and inhibiting inwardly and outwardly directed currents in a manner dependent upon [Ca^2+^]^i^ (see [664] for summary). Considerable crossover in pharmacology with large conductance Ca^2+^-activated K^+^ channels also exists (see [400] for overview). Two novel compounds, CaCCinh-A01 and CaCCinh-B01 have been identified as blockers of calcium-activated chloride channels in T84 human intestinal epithelial cells [246]). Significantly, other novel compounds totally block currents mediated by TMEM116A, but have only a modest effect upon total current mediated by CaCC native to T84 cells or human bronchial epithelial cells, suggesting that TMEM16A is not the predominant CaCC in such cells [860]. CaMKII modulates CaCC in a tissue dependent manner (reviewed by [439, 664]). CaMKII inhibitors block activation of I^Cl(Ca)^ in T84 cells but have no effect in parotid acinar cells. In tracheal and arterial smooth muscle cells, but not portal vein myocytes, inhibition of CaMKII reduces inactivation of I^Cl(Ca)^. Intracellular Ins(3,4,5,6)P_4_ may act as an endogenous negative regulator of CaCC channels activated by Ca^2+^, or CaMKII. Smooth muscle CaCC are also regulated positively by Ca^2+^-dependent phosphatase, calcineurin (see [664] for summary).

## Maxi chloride channel


Ion channels→Other ion channels→Chloride channels→Maxi chloride channel

### Overview:

Maxi Cl^−^ channels are high conductance, anion selective, channels initially characterised in skeletal muscle and subsequently found in many cell types including neurones, glia, cardiac muscle, lymphocytes, secreting and absorbing epithelia, macula densa cells of the kidney and human placenta syncytiotrophoblasts [1050]. The physiological significance of the maxi Cl^−^ channel is uncertain, but roles in cell volume regulation and apoptosis have been claimed. Evidence suggests a role for maxi Cl^−^ channels as a conductive pathway in the swelling-induced release of ATP from mouse mammary C127i cells that may be important for autocrine and paracrine signalling by purines [310, 1049]. A similar channel mediates ATP release from macula densa cells within the thick ascending of the loop of Henle in response to changes in luminal NaCl concentration [93]. A family of human high conductance Cl^−^ channels (TTYH1-3) that resemble Maxi Cl^−^ channels has been cloned [1173], but alternatively, Maxi Cl^−^ channels have also been suggested to correspond to the voltage-dependent anion channel, VDAC, expressed at the plasma membrane [55, 903].

**Table:** 

Nomenclature	Maxi Cl-
Activators	cytosolic GTP*γ*S, extracellular chlorpromazine, extracellular tamoxifen, extracellular toremifene, extracellular triflupromazine
Endogenous channel blockers	intracellular arachidonic acid
Channel blockers	DIDS (pIC_50_ 4.4) [1085], extracellular Zn^2+^ (pIC_50_ 4.3) [919], NPPB (pIC_50_ 3.8) [1085], extracellular Gd^3+^, SITS, diphenylamine-2-carboxylic acid
Functional Characteristics	*γ* = 280-430 pS (main state); permeability sequence, I > Br > Cl > F > gluconate (P_Cl_P_Cl_ = ~1.5); ATP is a voltage dependent permeant blocker of single channel activity (P_ATP_/P_Cl_ = 0.08-0.1); channel activity increased by patch-excision; channel opening probability (at steady-state) maximal within approximately ± 20 mV of 0 mV, opening probability decreased at more negative and (commonly) positive potentials yielding a bell-shaped curve; channel conductance and opening probability regulated by annexin 6
Comments	Maxi Cl^−^ is also activated by G protein-coupled receptors and cell swelling. Tamoxifen and toremifene are examples of triphenylethylene anti-oestrogens

### Comments:

Differing ionic conditions may contribute to variable estimates of *γ* reported in the literature. Inhibition by arachidonic acid (and cis-unsaturated fatty acids) is voltage-independent, occurs at an intracellular site, and involves both channel shut down (K_*d*_ = 4-5 *μ*M) and a reduction of *γ* (K_*d*_ = 13-14 *μ*M). Blockade of channel activity by SITS, DIDS, Gd^3+^ and arachidonic acid is paralleled by decreased swelling-induced release of ATP [310, 1049]. Channel activation by anti-oestrogens in whole cell recordings requires the presence of intracellular nucleotides and is prevented by pre-treatment with 17*β*-estradiol, bucladesine, or intracellular dialysis with GDP*β*S [275]. Activation by tamoxifen is suppressed by low concentrations of okadaic acid, suggesting that a dephosphorylation event by protein phosphatase PP2A occurs in the activation pathway [275]. In contrast, 17*β*-estradiol and tamoxifen appear to directly inhibit the maxi Cl^−^ channel of human placenta reconstituted into giant liposomes and recorded in excised patches [1024].

## Volume regulated chloride channels (VRAC)


Ion channels→Other ion channels→Chloride channels→Volume regulated chloride channels (VRAC)

### Overview:

Volume regulated chloride channels (also termed VSOAC, volume-sensitive organic osmolyte/anion channel; VRC, volume regulated channel and VSOR, volume expansion-sensing outwardly rectifying anion channel) participate in regulatory volume decrease (RVD) in response to cell swelling. VRAC may also be important for several other processes including the regulation of membrane excitability, transcellular Cl^−^ transport, angiogenesis, cell proliferation, necrosis, apoptosis, glutamate release from astrocytes, insulin (*INS*, P01308) release from pancreatic *β* cells and resistance to the anti-cancer drug, cisplatin (reviewed by [96, 844, 878, 905]). VRAC may not be a single entity, but may instead represent a number of different channels that are expressed to a variable extent in different tissues and are differentially activated by cell swelling. In addition to ClC-3 expression products (see above) several former VRAC candidates including *MDR1* (ABCB1 P-glycoprotein), Icln, Band 3 anion exchanger and phospholemman are also no longer considered likely to fulfil this function (see reviews [878, 1064]).

**Table:** 

Nomenclature	VRAC
Activators	GTP*γ*S
Endogenous channel blockers	intracellular Mg^2+^, arachidonic acid
Channel blockers	1,9-dideoxyforskolin, 9-anthroic acid, DCPIB, DIDS, IAA-94, NPPB, NS3728, carbenoxolone, clomiphene, diBA-(5)-C4, gossypol, mefloquine, mibefradil, nafoxidine, nordihydroguiaretic acid, quinidine, quinine, tamoxifen
Functional Characteristics	*γ* = 10-20 pS (negative potentials), 50-90 pS (positive potentials); permeability sequence SCN > I > NO_3_^−^ >Br^−^ > Cl^−^ > F^−^ > gluconate; outward rectification due to voltage dependence of *γ*; inactivates at positive potentials in many, but not all, cell types; time dependent inactivation at positive potentials; intracellular ionic strength modulates sensitivity to cell swelling and rate of channel activation; rate of swelling-induced activation is modulated by intracellular ATP concentration; ATP dependence is independent of hydrolysis and modulated by rate of cell swelling; inhibited by increased intracellular free Mg^2+^ concentration; swelling induced activation of several intracellular signalling cascades may be permissive of, but not essential to, the activation of VRAC including: the Rho-Rho kinase-MLCK; Ras-Raf-MEK-ERK; PIK3-NOX-H_2_O_2_ and Src-PLC*γ*-Ca^2+^ pathways; regulation by PKC*α* required for optimal activity; cholesterol depletion enhances activity; activated by direct stretch of *β*1 -integrin
Comments	VRAC is also activated by cell swelling and low intracellular ionic strength. VRAC is also blocked by chromones, extracellular nucleotides and nucleoside analogues

### Comments:

In addition to conducting monovalent anions, in many cell types the activation of VRAC by a hypotonic stimulus can allow the efflux of organic osmolytes such as amino acids and polyols that may contribute to RVD.

### Comments on Chloride channels: Other chloride channels

In addition to some intracellular chloride channels that are not considered here, plasma membrane channels other than those listed have been functionally described. Many cells and tissues contain outwardly rectifying chloride channels (ORCC) that may correspond to VRAC active under isotonic conditions. A cyclic AMP-activated Cl^−^ channel that does not correspond to CFTR has been described in intestinal Paneth cells [1229]. A Cl channel activated by cyclic GMP with a dependence on raised intracellular Ca^2+^ has been recorded in various vascular smooth muscle cells types, which has a pharmacology and biophysical characteristics very different from the ’conventional’ CaCC [789, 976]. It has been proposed that bestrophin-3 (*BEST3*, Q8N1M1) is an essential component of the cyclic GMP-activated channel [790]. A proton-activated, outwardly rectifying anion channel has also been described [655].

The chloride intracellular channel proteins (CLICs) are non-canonical ion channels with six homologs, distinct from most ion channels in that they have both soluble and integral membrane forms. The physiological role of CLICs appears to be maintenance of intracellular membranes, which is associated with tubulogenesis but may involve other substructures [418].

## Connexins and Pannexins


Ion channels→Other ion channels→Connexins and Pannexins

### Overview:

Gap junctions are essential for many physiological processes including cardiac and smooth muscle contraction, regulation of neuronal excitability and epithelial electrolyte transport [138, 222, 334]. Gap junction channels allow the passive diffusion of molecules of up to 1,000 Daltons which can include nutrients, metabolites and second messengers (such as IP_3_) as well as cations and anions. 21 connexin genes and 3 pannexin genes which are structurally related to the invertebrate innexin genes, code forgap junction proteins in humans. Each connexin gap junction comprises 2 hemichannels or ’connexons’ which are themselves formed from 6 connexin molecules. The various connexins have been observed to combine into both homomeric and heteromeric combinations, each of which may exhibit different functional properties. It is also suggested that individual hemichannels formed by a number of different connexins might be functional in at least some cells [456]. Connexins have a common topology, with four *α*-helical transmembrane domains, two extracellular loops, a cytoplasmic loop, and N- and C-termini located on the cytoplasmic membrane face. In mice, the most abundant connexins in electrical synapses in the brain seem to be Cx36, Cx45 and Cx57 [1132]. Mutations in connexin genes are associated with the occurrence of a number of pathologies, such as peripheral neuropathies, cardiovascular diseases and hereditary deafness. The pannexin genes Px1 and Px2 are widely expressed in the mammalian brain [1256]. Like the connexins, at least some of the pannexins can form hemichannels [138, 955].

**Table:** 

Nomenclature	Cx23	Cx25	Cx26	Cx30	Cx30.2	Cx30.3	Cx31
HGNC, UniProt	*GJE1*, A6NN92	*GJB7*, Q6PEY0	*GJB2*, P29033	*GJB6*, O95452	*GJC3*, Q8NFK1	*GJB4*, Q9NTQ9	*GJB3*, O75712
Endogenous inhibitors	extracellular Ca^2+^ (blocked by raising external Ca^2+^)						
Inhibitors	carbenoxolone, flufenamic acid, octanol						

**Table:** 

Nomenclature	Cx31.1	Cx31.9	Cx32	Cx36	Cx37	Cx40	Cx40.1
HGNC, UniProt	*GJB5*, O95377	*GJD3*, Q8N144	*GJB7*, P08034	*GJD2*, Q9UKL4	*GJA4*, P35212	*GJAS*, P36382	*GJD4*, Q96KN9
Endogenous inhibitors	extracellular Ca^2+^ (blocked by raising external Ca^2+^)						
Inhibitors	carbenoxolone, flufenamic acid, octanol						

**Table:** 

Nomenclature	Cx43	Cx45	Cx46	Cx47	Cx50	Cx59	Cx62
HGNC, UniProt	*GJA1*, P17302	*GJC1*, P36383	*GJA3*, Q9Y6H8	*GJC2*, Q5T442	*GJA8*, P48165	*GJA9*, P57773	*GJA10*, Q969M2
Endogenous inhibitors	extracellular Ca^2+^ (blocked by raising external Ca^2+^)						
Inhibitors	carbenoxolone, flufenamic acid, octanol						

**Table:** 

Nomenclature	Px1	Px2	Px3
HGNC, UniProt	*PANX1*, Q96RD7	*PANX2*, Q96RD6	*PANX3*, Q96QZ0
Inhibitors	carbenoxolone, flufenamic acid (little block by flufenamic acid)	carbenoxolone, flufenamic acid (little block by flufenamic acid)	carbenoxolone, flufenamic acid (little block by flufenamic acid)
Comments	Electrophysiological studies demonstrate that endogenously expressed hPx1 forms intercellular channels with distinct voltage-dependent properties [925]. Channel function is unaffected by raising external Ca^2+^.	Unaffected by raising external Ca^2+^	Unaffected by raising external Ca^2+^

### Comments:

Connexins are most commonly named according to their molecular weights, so, for example, Cx23 is the connexin protein of 23 kDa. This can cause confusion when comparing between species- for example, the mouse connexin Cx57 is orthologous to the human connexin Cx62. No natural toxin or specific inhibitor of junctional channels has been identified yet however two compounds often used experimentally to block connexins are carbenoxolone and flufenamic acid [1056]. At least some pannexin hemichannels are more sensitive to carbenoxolone than connexins but much less sensitive to flufenamic acid [137]. It has been suggested that 2-aminoethoxydiphenyl borate (2-APB) may be a more effective blocker of some connexin channel subtypes (Cx26, Cx30, Cx36, Cx40, Cx45, Cx50) compared to others (Cx32, Cx43, Cx46, [56]).

## Piezo channels


Ion channels→Other ion channels→Piezo channels

### Overview:

Piezo proteins are the pore-forming subunits of trimeric mechanosensitive ion channels that open in response to mechanical stimuli such as shear stress and membrane stretch, allowing positively charged ions, including calcium, to flow into the cell. Piezo orthologs have thus far been identified in numerous eukaryotes. Most vertebrates have two channel isoforms, Piezo1 and Piezo2. Across species, Piezos are very large proteins (2521 and 2752 amino acids for human Piezo1 and human Piezo2, respectively) with numerous (>14) predicted TM domains per subunit and, strikingly, no homology to other known proteins [1332]. Piezo channels play a critical role in sensory neuron transduction [847, 1395]

**Table:** 

Nomenclature	Piezo1	Piezo2
HGNC, UniProt	*PIEZO1*, Q92508	*PIEZO2*, Q9H5I5
Selective activators	Yodal (pEC_50_ 4.6) [1176], Jedi2 (pEC_50_ 3.8) [1297] – Mouse, Jedi1 [1297] – Mouse	–
Inhibitors	Dooku1 (pIC_50_ 5.8) [333]	–
Functional Characteristics	Mechano-activated	Mechano-activated

### Comments:

Yoda1 is a Piezo1 channel activator [333, 1290].

## Sodium leak channel, non-selective (Na_Vi_)


$$\text{Ion channels}\to \text{Other ion channels}\to \text{Sodium leak channel},\;\text{non-selective}\;({\text{Na}}_{\text{Vi}})$$

### Overview:

The sodium leak channel, non selective (**NC-IUPHAR tentatively recommends the nomenclature Na_Vi_2.1, W.A. Catterall, personal communication**) is structurally a member of the family of voltage-gated sodium channel family (Na_v_1.1-Na_v_1.9) [668, 1379]. In contrast to the latter, Na_Vi_2.1, is voltage-insensitive (denoted in the subscript ‘vi’ in the tentative nomenclature) and possesses distinctive ion selectivity and pharmacological properties. Na_Vi_2.1, which is insensitive to tetrodotoxin (10 *μ*M), has been proposed to mediate the tetrodotoxin-resistant and voltage-insensitive Na^+^ leak current (I_L_-Na) observed in many types of neurone [737]. However, whether Na_Vi_2.1 is constitutively active has been challenged [1175]. Na_Vi_2.1 is widely distributed within the central nervous system and is also expressed in the heart and pancreas specifically, in rodents, within the islets of Langerhans [668, 737]. Na_Vi_2.1 has been proposed to be a core effector for the action of inhibitory G proteins [968].

**Table:** 

Nomenclature	Na_vi_2.1
HGNC, UniProt	*NALCN*, Q8IZF0
Activators	Constitutively active (Lu *et al*., 2007), or activated downstream of Src family tyrosine kinases (SFKs) (Lu *et al*.,2009; Swayne *et al*., 2009); positively modulated by decreased extracellular Ca^2+^ concentration (Lu *et al*., 2010) [737, 738, 739, 1175]
Channel blockers	Gd^3+^ (pIC_50_ 5.6), Cd^2+^ (pIC_50_ 3.8), Co^2+^ (pIC_50_ 3.6), verapamil (pIC_50_ 3.4)
Functional Characteristics	*γ* = 27 pS (by fluctuation analysis), *P*_Na_/*P*_Cs_ = 1.3, *P*_K_/*P*_Cs_ = 1.2, *P*_Ca_/*P*_Cs_ = 0.5, linear current voltage-relationship, voltage-independent and non-inactivating

### Comments:

In native and recombinant expression systems Na_Vi_2.1 can be activated by stimulation of NK_1_ (in hippocampal neurones), neurotensin (in ventral tegmental area neurones) and M3 muscarinic acetylcholine receptors (in MIN6 pancreatic *β*-cells) and in a manner that is independent of signalling through G proteins [738, 1175]. Pharmacological and molecular biological evidence indicates such modulation to occur though a pathway that involves the activation of Src family tyrosine kinases. It is suggested that Na_Vi_2.1 exists as a macromolecular complex with M3 receptors [1175] and peptide receptors [738], in the latter instance in association with the protein UNC-80, which recruits Src to the channel complex [738, 1282]. By contrast, stimulation of Na_vi_2.1 by decreased extracellular Ca^2+^ concentration is G protein dependent and involves a Ca^2+^-sensing G protein-coupled receptor and UNC80 which links Na_vi_2.1 to the protein UNC79 in the same complex [739]. Na_vi_2.1 null mutant mice have severe disturbances in respiratory rhythm and die within 24 hours of birth [737]. Na_vi_2.1 heterozygous knockout mice display increased serum sodium concentrations in comparison to wildtype littermates and a role for the channel in osmoregulation has been postulated [1115].

## Orai channels


Ion channels→Other ion channels→Store-operated ion channels→Orai channels

### Overview:

Orai channels are pore forming proteins which underlie calcium release-activated calcium (CRAC) channels. When calcium is lost from the endoplasmic reticulum, they form complexes with STIM (stromal interaction molecule) proteins to trigger calcium entry following store depletion [689].

**Table:** 

Nomenclature	Orai1	Orai2	Orai3
HGNC, UniProt	*ORAI1*, Q96D31	*ORAI2*, Q96SN7	*ORAI3*, Q9BRQ5
Comments	*ORAI1* is the gene that encodes the prototypical CRAC store-operated Ca^2+^ entry (SOCE) channel. CRAC channels are activated by depletion of Ca^2+^ in the endoplasmic reticulum that results from antigen-induced activation of a range of immunoreceptors (including TCRs, BCRs, Fc*γ* and Fc*ϵ* receptors, chemokine GPCRs and some innate pattern-recognition receptors).	–	–

### Comments:

The pathophysiological effect of functional CRAC channel deficiency can be caused by loss-of-function mutations in *ORAI1* or STIM molecules. Such CRAC channelopathies are characterised by impaired immune cell function and have been identified as an underlying cause of primary immunodeficiency with predominant features that resemble severe combined immunodeficiency disease.

ORAI1 and ORAI2 proteins form heteromeric complexes that constitute the pore of Ca^2+^ release-activated Ca^2+^ (CRAC) channels. Mice with double *Orai1/Orai2* knockout have severely impaired T cell function.
